# Comprehensive Discrimination of Amomi Fructus From Different Origins Using UHPLC‐Q‐Orbitrap MS, HS–GC–MS/MS, NMR and MIR Technologies Based On Data Fusion Strategies

**DOI:** 10.1002/ansa.70029

**Published:** 2025-07-27

**Authors:** Yuxin Zhang, Yihang Li, Ze Li, Zhonglian Zhang, Yue Zhang, Biying Chen, Lixia Zhang, Meifang Song, Miaomiao Jiang

**Affiliations:** ^1^ Department of Pharmaceutical, Tianjin Baodi People's Hospital Tianjin Baodi Hospital Tianjin P. R. China; ^2^ State Key Laboratory of Component‐Based Chinese Medicine Tianjin Key Laboratory of TCM Chemistry and Analysis Haihe Laboratory of Modern Chinese Medicine Tianjin University of Traditional Chinese Medicine Tianjin P. R. China; ^3^ Yunnan Key Laboratory of Southern Medicine Utilization, Yunnan Branch of Institute of Medicinal Plant Development, Chinese Academy of Medical Sciences Peking Union Medical College Jinghong P. R. China; ^4^ Department of Pharmaceutical The Second Affiliated Hospital of Guangxi Medical University Nanning Guangxi P. R. China

**Keywords:** Amomi Fructus, data fusion, HS–GC–MS/MS, middle‐infrared (MIR), nuclear magnetic resonance (NMR), ultra‐high‐performance liquid chromatography system coupled with quadrupole‐Orbitrap mass spectrometer (UHPLC‐Q‐Orbitrap MS)

## Abstract

Amomi Fructus (SR) is an important edible herb widely used as a spice and traditional Chinese medicine. To comprehensively solve the serious practical problems of origins and species confusion in SR, the systematic characterization methods were established by liquid chromatography–mass spectrometer, gas chromatography–mass spectrometer, nuclear magnetic resonance and infrared spectroscopy. A total of 286 compounds and functional group information were detected. The classification of SR from different origins was performed by data fusion models built using random forest (RF) and other algorithms. A mid‐level data fusion model (an RF model established after combining the features selected by RF and RF–RF) performed the best classification. Then 27 differential compounds (including flavonoids, polyphenols and terpenoids) and their functional group information were screened for external verification and could significantly improve the groups’ separation effect just by simple principal component analysis. A more comprehensive and accurate means of analysis was found.

## 1 Introduction

Amomi Fructus (Sharen, SR) is a widely used edible herb and traditional Chinese medicine (TCM), which is not only widely used as a type of condiment and spice but also often used clinically in the treatment of gastrointestinal diseases. There are several plant resources for this edible herb, including *Amomum villosum* Lour. (Yangchunsha, YCS), *A. villosum* Lour. var. xanthioides T. L. Wu et Senjen (Lvqiaosha, LQS) and *Amomum longiligulare* T.L.Wu (Hainansha, HNS) [1]. According to literature [2, 3, 4, 5, 6, 7, 8], SR contains different kinds of volatile oils, flavonoids and polysaccharides. It is widely used in daily life such as soup, tea and porridge. As natural herbs, the quality of SR is closely related to their origins and species. The habitat area of SR has been recorded in many southern parts of China, including Yunnan, Guangxi and Guangdong provinces. It also can be found in the market of Southeast Asia, such as Myanmar, Laos and other countries [3]. The samples collected in this study covered almost all the major producing areas of SR in the world, including Yunnan (YN), Guangxi (GX), Guangdong (GD), Fujian (FJ) and Myanmar (MD), and the varieties included YCS and LQS. As we all know, the composition of TCM is too complex, and only one or two technologies are not enough to profile the differences between different species. Due to the similar morphology of SR in different sources, it is impossible to accurately identify them even with the latest DNA barcoding technology, which is good at authenticating closely related species [3], and the high content of SR chemical components and main active ingredients in different regions has increased the workload and difficulty of quality assessment. In recent years, the research on SR has been mainly based on gas chromatography–mass spectrometry (GC–MS) and electronic nose technology with little about distinguishing different sources, and the sources of SR supplied in the market are complex with uneven quality [9, 10, 11]. Therefore, to control its quality effectively, it is necessary to use different techniques to detect SRs from different sources and make comprehensive analysis and differentiation through data fusion.

GC–MS has a good advantage for the detection of volatile substances, and the GC–MS databases for substance characterization are relatively reliable. After equipping with a headspace sampler (HS), volatile compounds in the samples can be volatilized by heating, and the sample pretreatment steps were further simplified. On the contrary, liquid chromatography–mass spectrometry (LC–MS) is quite suitable for the analysis of non‐volatile metabolites with high boiling points and the separation and identification of weak‐polar compounds after equipping with a reverse‐phase chromatography column. Both LC–MS and GC–MS could combine the rapid separation ability of chromatography with the precise qualitative and quantitative capacities of MS for characteristic ions, and both of them have the advantages of high sensitivity, strong specificity and accurate and reliable analysis results. Nuclear magnetic resonance (NMR) techniques generally include hydrogen nuclear magnetic resonance spectroscopy (^1^H‐NMR) and carbon‐13 nuclear magnetic resonance spectroscopy (^13^C‐NMR) with many advantages, including fast analysis speed, high efficiency, no damage to the sample, simple sample pretreatment, simultaneous qualitative and quantitative analysis and wide detective range, which is quite suitable for analysis of higher polar compounds. Infrared (IR) technology is a universal method that investigates the generation, propagation, transformation, measurement and application of infrared radiation, which could be further divided into near‐infrared (NIR), middle‐infrared (MIR) and far‐infrared (FIR) according to the wavelength range. Many important chemicals have specific absorption and emission lines in the MIR band. MIR technology can provide functional group information of compounds in SR to assist in identification [12, 13, 14]. These techniques have been widely used in the analysis of food, medicinal materials and biological samples in recent years [15, 16, 17, 18, 19]. To conduct a comprehensive characterization of various types of compounds in SR, it is necessary to combine these technical means to analyse SR.

Data fusion is a process of integrating data blocks from multiple sources or sensors into a single comprehensive model. Fused information from different detection sources could exploit the characteristics of samples more comprehensively and provide the potential to obtain more accurate classification. Data fusion can be implemented using chemometric techniques, which require the collected data to have a relatively high quality and can be realized at three levels, designated as low‐, mid‐ and high‐level fusion. Low‐level data fusion (LLDF) integrates multiple data sources by concatenating data blocks of different natures, taking into account all the variables collected in the different blocks. Mid‐level (MLDF) or high‐level (HLDF) data fusion is a feature‐level fusion involving variable screening. Before the fusion process, a variable selection technique was used to extract important variables from each data source individually as features. These features (i.e., characteristic variables) can then be used for classification and prediction. Both LLDF and MLDF combine data sources at the data level, whereas HLDF is a decision‐level fusion that develops separate models for each available data block and combines their responses to produce the final fusion response [20, 21, 22]. In recent years, data fusion has been widely used in the quality assessment of food and herbs [23, 24, 25, 26, 27]. To comprehensively explore the various compounds contained in SR samples for more accurate classification according to different origins and species, it is necessary to integrate the data of above‐mentioned four technologies and formulate corresponding data fusion strategies.

To achieve data fusion, principal component analysis (PCA), partial least squares discrimination analysis (PLS‐DA), support vector machine (SVM), *k* nearest neighbour (*k*NN), neural network (NN), decision tree (DT) and random forest (RF) algorithms are popularly used to achieve model establishment. PCA is a common unsupervised data analysis method, which is often used for dimensionality reduction of high‐dimensional data and can be used to extract the main feature components of data [28]. PLS‐DA is a supervised multivariate statistical analysis method for discriminant analysis, which is suitable for analysing data with small differences and avoiding data with large sample sizes dominating models [29]. SVM is a linear supervised machine learning method to deal with non‐linear data sets for classification and regression problems. For classification, the original data matrix is mapped to a new higher dimensional space using a kernel function, including linear, polynomial (poly), radial basis function (RBF) and sigmoid kernel function [28]. *k*NN is a data exploit classification algorithm that classifies samples by measuring the Euclidean distance among different eigenvalues. Each sample can be represented by its nearest *k* neighbours [30]. NN is a mathematical model that imitates the structure and function of biological NNs and is mainly composed of the input layer, hidden layer and output layer [31]. DT is a basic classification and regression method, which generalizes a set of classification rules from a given training set and builds a DT model that can classify instances correctly [32]. RF is an ensemble of tree‐structured algorithms for both classification and regression practices, which combine the theory of bootstrap aggregating, random split selection and random subspace by Breiman. It is an extension of the Bagging algorithm (a parallel ensemble learning method), and its base learner is DT [33]. In recent years, the algorithms mentioned above are often applied for data fusion analysis, covering most data types [34, 35, 36]. Among them, the principle and classification effect of PLS‐DA is similar to orthogonal partial least squares discriminant analysis (OPLS‐DA), and the PLS‐DA model was selected for analysis in this study. In addition, to verify the quality of the model and enhance the credibility of the model, it is necessary to use part of the data for external verification [35, 36, 37, 38, 39].

In the present study, an effective strategy was proposed for comprehensive analysis and differentiation of SRs, which was based on the technologies, including an ultra‐high‐performance liquid chromatography system coupled with quadrupole‐Orbitrap mass spectrometer (UHPLC‐Q‐Orbitrap MS), to detect non‐volatile compounds, a gas chromatography/triple quadrupole mass spectrometry system coupled with headspace sampler (HS–GC–MS/MS), to analyse volatile compounds, a 600 MHz NMR spectrometer, to supplement the results of UHPLC‐Q‐Orbitrap MS, and a Fourier transform MIR spectroscopy, to make a fast detection, and data fusion algorithms, to integrate data sets and obtain more accurate classification.

**FIGURE 1:**
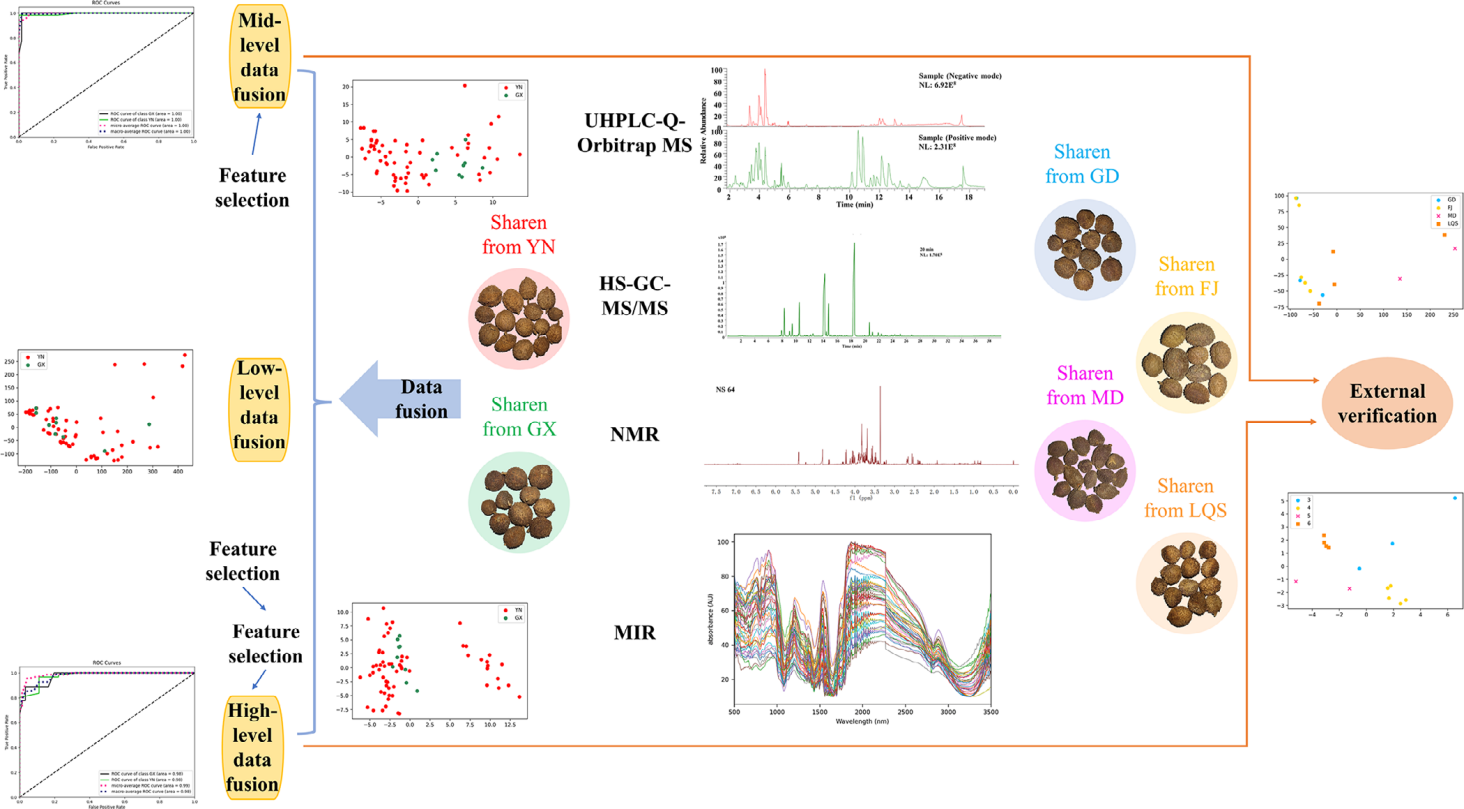
Schematic flowchart for the comprehensive discrimination of Amomi Fructus from different origins using UHPLC‐Q‐Orbitrap MS, HS–GC–MS/MS, NMR and MIR technologies based on data fusion strategies. HS–GC–MS/MS, gas chromatography/triple quadrupole mass spectrometry system coupled with headspace sampler; LQS, Lvqiaosha; MIR, middle‐infrared; NMR, nuclear magnetic resonance; UHPLC‐Q‐Orbitrap MS, ultra‐high‐performance liquid chromatography system coupled with quadrupole‐Orbitrap mass spectrometer.

The general schematic flowchart is displayed in Figure 1. First, the multi‐component characterization of SRs was performed by UHPLC‐Q‐Orbitrap MS, HS–GC–MS/MS, NMR and MIR. Second, the classification of SRs from different origins and species was performed by LLDF, MLDF and HLDF models, including PCA, PLS‐DA, SVM, *k*NN, NN, DT and RF. Third, the model with the best classification effect was selected, and characteristic variables were obtained for external verification. To the best of our knowledge, this was the first study to report comprehensive multi‐components characterization of SRs by UHPLC‐Q‐Orbitrap MS, HS–GC–MS/MS, NMR and MIR technologies to classify SRs from different origins and species by data fusion models, which provided a good basis for further quality control (QC) of SRs.

## 2 Materials and Methods

### 2.1 Reagents and Solutions

Fifty‐two reference compounds, including sixteen organic acids (1, β‐d‐glucopyranuronic acid; 2, glutaric acid; 3, citric acid; 4, shikimic acid; 5, 3‐amino‐4‐hydroxybenzoic acid; 6, 3‐hydroxybutyric acid; 7, cinnamic acid; 8, 4‐hydroxybenzoic acid; 9, sinapic acid; 10, salicylic acid; 11, adipic acid; 12, *p*‐coumaric acid; 13, caffeic acid; 14, vanillic acid; 15, ferulic acid; 16, linoleic acid), twelve amino acids (17, l‐histidine; 18, γ‐aminobutyric acid; 19, arginine; 20, d‐proline; 21, l‐threonine; 22, phenylalanine; 23, glutamic acid; 24, l‐tyrosine; 25, norleucine; 26, d‐tryptophan; 27, l‐valine; 28, l‐leucine), one alkaloids (29, berberine), six flavonoids (30, quercetin‐7‐*O*‐β‐d‐glucoside; 31, kaempferol; 32, taxifolin; 33, rutin; 34, isoquercitrin; 35, luteolin; 36, quercetin), two coumarins (37, scopoletin; 38, coumarin), five polyphenols (39, chlorogenic acid; 40, catechin; 41, 3,4‐dihydroxybenzaldehyde; 42, vanillin; 43, genistein), three terpenoids (44, camphor; 45, abscisic acid; 46, caryophyllene oxide) and six other compounds (47, xylitol; 48, vitamin C; 49, pantothenic acid; 50, 3‐hydroxybenzyl alcohol; 51, 4‐hydroxybenzaldehyde; 52, oleamide), were provided by Shanghai Yuanye Biotech. Co. Ltd. (Shanghai, China), National Institute for the Control of Pharmaceutical and Biological Products (Shanghai, China), Sichuan Weikeqi Bio‐Technology Co. Ltd. (Sichuan, China) and Chengdu Must Bio‐Technology Co. Ltd. (Chengdu, China). The experimental reagents were HPLC‐grade acetonitrile, methanol (Fisher, Fair Lawn, NJ, USA), formic acid (ACS, Wilmington, DE, USA; FA), acetic acid (Sigma Aldrich, St. Louis, MO, USA; HOAc), deuterium oxide (Cambridge Isotope Laboratories Inc., USA; D_2_O), 3‐(trimethylsilyl) propionic‐2,2,3,3‐*d*_4_ acid sodium salt (Sigma‐Aldrich Co. LLC., USA; TSP) and potassium hydrogen phosphate anhydrous and monosodium phosphate (Shanghai Macklin Biochemical Technology Co. Ltd.). Disposable IR cards (KBr window, 25 mm × 4 mm) were purchased from Tianjin Tianguang Optical Instrument Co. Ltd. (Tianjin, China). Deionized water was prepared by a Milli‐Q A10 water purification system (Millipore, Bedford, MA, USA).

### 2.2 Sample Preparation

Eighty‐four samples (batch numbers and detailed information shown in Table S1) were, respectively, reduced to powder by a high‐speed multi‐functional crusher (Wuyi Haina Electric Co. Ltd., Zhejiang, China) and kept in the desiccator at ambient temperature.

### 2.3 Sample Measurement

#### 2.3.1 UHPLC‐Q‐Orbitrap MS Analysis

A total of 84 SR samples were, respectively, weighed at 0.5 g and dissolved in 10 mL 50% methanol‐H_2_O (v/v). After vortexing (Vortex mixer XW‐80A, Shanghai Luxi analytical instrument factory, China) for 2 min and extracting in an ultrasonic water bath (Kunshan Ultrasonic Instrument Co. Ltd., China) for 60 min, each liquid was centrifuged (Eppendorf 5424R, Barkhausenweg 1, Hamburg, Germany) at 13,200 *g* for 20 min. Then supernatants (50 mg mL^−1^) were, respectively, filtered by 0.22 µm PTFE syringe filters and analysed by Vanquish UHPLC system coupled with Thermo Fisher Orbitrap Exploris 120 MS (Thermo Fisher Scientific, San Jose, CA, USA). To ensure the UHPLC‐Q‐Orbitrap MS‐based metabolomics data quality, 84 samples of equal amount (0.5 g each) were evenly mixed, and then 0.5 g was taken from the mixed powder to make a pooled QC sample according to the above steps. The QC sample was injected six times at the beginning of the analysis batch and then run one time after every six samples.

As shown in Figures S1–S17 and Table S2, a Waters ACQUITY UHPLC HSS T_3_ column (2.1 mm × 100 mm, 1.8 µm, Waters, Milford, MA) maintained at 35°C was used to achieve chromatographic separation, which was eluted by a binary mobile phase composed of water containing 0.1% FA (A) and acetonitrile (D) running at a flow rate of 0.3 mL min^−1^. The injection volume for each sample of 2 µL was set. A gradient elution programme was as follows: 0–2 min, 5%–20%D; 2–8 min, 20%–45%D; 8–8.5 min, 45%–70%D; 8.5–14.5 min, 70%–90%D; 14.5–15 min, 90%–100%D; 15–15.5 min, 100%D; 15.5–16 min, 100%–5%D; 16–19 min, 5%D.

HESI source parameters were set as follows: spray voltage (SV), −3.5 kV/+3.5 kV; sheath gas pressure, 35 arb (arbitrary unit); auxiliary gas pressure, 10 arb; sweep gas pressure, 0 arb; capillary temperature (CT), 320°C; and aux gas heating temperature (AGHT), 300°C. A full MS/dd‐MS^2^ (TopN) scan approach was applied to achieve a comprehensive characterization. The mass range was set at *m/z* 100–1500, and the resolution was set to 70,000. The normalized collision energy (NCE) was set to 20%, 40% and 60%.

#### 2.3.2 HS–GC–MS/MS Analysis

The SR samples were, respectively, weighed at 0.5 g, added into 20 mL headspace vials, and analysed by Agilent 8890/7000E GC/triple quadrupole MS systems coupled with 7697A GC HS (Agilent Technologies Inc., Santa Clara, USA; HS–GC–MS/MS).

According to the relevant literature [9, 10, 11] and experimental results shown in Figures S18 and S19, the temperature programming and other conditions were set as follows, obtaining the best resolution and response.

Each vial was heated at 100°C for 20 min, and the volatile compounds absorbed were separated by an Agilent HP‐5MS column (250 µm × 30 m, 0.25 µm). Helium was used as the carrier gas with a flow rate of 1.0 mL min^−1^. The column oven temperature programme was set as follows: The initial temperature was set at 50°C (held for 3 min), then increased to 100°C (held for 2 min) at a rate of 6°C min^−1^, increased to 140°C (held for 2 min) at a rate of 6°C min^−1^ and increased to 185°C (held for 3 min) at a rate of 4°C min^−1^. The temperature was subsequently increased to 230°C (held for 2 min) at a rate of 30°C min^−1^. The injector temperature was set at 230°C.

The temperature of the ion source and transfer was both set to 280°C. Additionally, the ionization mode was electron impact (EI) mode, with an electron energy of 70 eV and a mass scan range of 35–500 *m/z*.

#### 2.3.3 NMR Analysis

All the SR samples were, respectively, weighed at 0.5 g and dissolved in 1 mL methanol. After vortexing for 2 min and ultrasonic‐extracting for 30 min, each liquid was centrifuged at 13,200 *g* for 10 min. Then supernatants (500 mg mL^−1^) were, respectively, dried under N_2_ gas using a termovap sample concentrator (NDK200‐1, MIULAB, Zhejiang, China). Phosphate‐buffer saline (PBS) was prepared by dissolving K_2_HPO_4_–NaH_2_PO_4_ (0.1 M) and TSP (0.15 mM) in D_2_O. The dried samples were, respectively, redissolved in 0.5 mL PBS (pH 7.40). After vortexing for 2 min and centrifuged at 13,200 *g* for 5 min, each supernatant was transferred to a 5 mm NMR tube and analysed by a Bruker Ascend 600 MHz NMR spectrometer (Bruker, Germany) equipped with an inverse cryogenic probe (TXI) at 298 K.

Chemical shifts (*δ*) are given in parts per million (ppm). According to the relevant literature [34] and experimental results shown in Figures S20 and S21, the NMR conditions were set as follows. The NMR data for metabolomics were all acquired using a standard one‐dimensional nuclear Overhauser effect spectroscopy pulse sequence with water suppression over a spectral width of 16 ppm, and the water signal was suppressed at 4.90 ppm. Bruker sequences, including noesygppr1d and c13cpd, were used for ^1^H NMR and ^13^C NMR with 64 and 1024 frequency domain scans, respectively, acquired with 64 k data points and 2 s relaxation delay. Data acquisition was automatically performed using Icon‐NMR on Topspin 3.5 (Bruker, BioSpin, Germany).

#### 2.3.4 MIR Analysis

SR samples were, respectively, weighed at 0.5 g and dissolved in 1 mL methanol. After vortexing for 2 min and ultrasonic‐extracting for 30 min, each liquid was centrifuged at 13,200 *g* for 10 min. Then supernatants (500 mg mL^−1^) were analysed by Varian 640 IR Fourier transform infrared spectroscopy (Thermo Fisher Scientific, Bremen, Germany; MIR).

The MIR spectrometer was first turned on and warmed up for half an hour. Each spectrum was an average of 16 scans with a resolution of 4 cm^−1^ across the wavenumbers from 4000 to 400 cm^−1^ at ambient temperature. The spectra were recorded as reference absorbance values in air.

### 2.4 Data Pre‐Processing

The number of chromatographic peaks extracted by SIEVE 2.2 software (Thermo Fisher Scientific) to optimize the analysis conditions for UHPLC‐Q‐Orbitrap MS and the raw data obtained was performed with the Xcalibur 4.0 software (Thermo Fisher Scientific). After data acquisition, data processing was performed with Compound Discoverer 3.1 (Thermo Fisher Scientific). The metabolites were identified through reference standards (in‐house database) and online databases (mzCloud and PubChem).

The volatile compounds detected by HS–GC–MS/MS were identified by searching the NIST mass spectral library (matching score >80%) and referring to relevant literature.

NMR spectra were preprocessed with MestReNova software (MestReNova v 14.0.1, 2018, Mestrelab Research, Santiago de Compostela, Spain). For all spectra, automatic phase and baseline corrections were performed before the normalization and spectrum alignment, and the region buckets with a width of 0.002 ppm were divided from normalized ^1^H spectrum ranging from 0.4 to 12.0 ppm (exclude residual water *δ*_H_ 4.8–5.0 ppm) with chemical shift referenced to the TSP signal (*δ*_H_ 0.00 ppm) [34, 40].

Focusing on MIR data, according to the optimization results in Figures S22 and S23, the raw spectra were smoothed using a Savitzky–Golay filter (SG) to reduce the effect of random noise and preprocessed by vector normalization (VN) to correct spectral errors caused by particle scattering. Then several segment signals of processed spectra, from 4000 to 3500, 2400 to 2200 and 500 to 400 cm^−1^, were removed because they were not informative.

### 2.5 Modelling Individual Data Sets

To explore the necessity of data fusion, PCA was, respectively, performed on all 84 sample data sets (including the model set and external validation set) collected by UHPLC‐Q‐Orbitrap MS, HS–GC–MS/MS, NMR and MIR four techniques.

### 2.6 Data Sets Division

A data fusion approach was performed by the UHPLC‐Q‐Orbitrap MS, HS–GC–MS/MS, NMR and MIR data sets, and the *Z*‐score normalization (standardization, SS) process was used on the four independent data matrices to handle potential batch effects and ensure data consistency when integrating measurements from different instruments. Among all 84 samples (Table S1), 70 samples (Nos. 1–70, including two groups from different origins, YN and GX) of individuals from each sampling site were selected as the model data set for the model establishment, and the remaining 14 samples (Nos. 71–84, including four groups from different origins, GD, FJ, MD and LQS) were used as the validation set for external verification. The train_test_split function in Python was used to select the training set (70%) and test set (30%) from the model data set (model data set:test set = 49:21).

### 2.7 Data Fusion Strategies

The PCA, PLS‐DA, SVM, *k*NN, NN, DT and RF models based on individual model data sets were established.

#### 2.7.1 LLDF

In LLDF, data from four sources were directly concatenated without feature selection. PCA, a common unsupervised data analysis method, and PLS‐DA, a supervised multivariate statistical analysis method, were applied for LLDF. Furthermore, machine learning methods, including SVM, *k*NN, NN, DT and RF algorithms, were all applied for the LLDF model establishment. As the optimization results in Tables S3–S5, the RBF was selected as the kernel function of the SVM model. The number of neighbours (*k* value) was set to 3 for the *k*NN model. The hidden layers and their neurons were 500, 200 and 10, respectively, in the NN model. The parameters of the remaining algorithms were all set to default values (e.g., n_components was set to 2 in the PCA and PLS‐DA models, criterion was set to gini in the DT model, and n_estimators was set to 100 in the RF model). The results of modeling with the optimal parameters were shown in Figure S26.

#### 2.7.2 MLDF

In MLDF, feature selection was applied to each data source by dimensionality reduction before merging data sets. In this study, four types of dimensionality reduction methods were used, including PCA, PLS‐DA, DT and RF. According to the results in Figures S24 and S25 and Tables S6 and S7, almost 95% variance ratio explained by different numbers of principal components (PCs) and latent variables (LVs) were selected by PCA and PLS‐DA, and the first 40 important variables were selected by DT and RF. Besides, the RBF was selected as the kernel function of the SVM model. The number of neighbours (*k* value) was set to 3 for the *k*NN model. The hidden layers and their neurons were 500, 200 and 10, respectively, in the NN model. The results of modeling with the optimal parameters were shown in Figure S26.

#### 2.7.3 HLDF

In HLDF, data from four sources were analysed by RF, the corresponding prediction models were constructed separately, and the results from all models were then merged. As shown in Table S8, the first 120 and 40 important variables were successively selected by RF, before the selected features were combined, and the Gini indexes (importance of the features) of these variables from four data sets were multiplied by the same coefficient (0.25), respectively. These variables were merged (160 in total) and sorted according to the calculated Gini indexes, and the top 40 with higher importance were selected for RF modelling. The remaining parameters of RF model were all set to default values. The results of modeling with the optimal parameters were shown in Figure S26.

#### 2.7.4 Model Establishment and Evaluation

All algorithms were implemented in PyCharm Community Edition 2020.3.2 (JetBrains, Czech), a free and open‐source integrated development environment (IDE) in the Python programming language. The Packages used were listed as follows: pandas (version 1.1.5), numpy (version 1.19.5), sciPy (version 1.5.4), sklearn (version 0.0.post1), matplotlib (version 3.3.4), scikit‐learn (version 0.24.2) and scikit‐plot (version 0.3.7).

The performance of the model was assessed by the receiver operating characteristics (ROC) curves and parameters of the area under curve (AUC), out‐of‐bag error (OOB), accuracy train, accuracy test, precision score, *f*1 score and recall score. Values of them close to 1.0 indicate an excellent classification ability [33].

## 3 Results and Discussion

### 3.1 Comprehensive Characterization of Multi‐Components in SR

By UHPLC‐Q‐Orbitrap MS analysis, a total of 171 compounds were detected (including 13 amino acids, 3 polysaccharides, 3 alcohols, 2 glycosides, 65 organic acids, 2 alkaloids, 5 polyphenols, 9 flavonoids, 3 coumarins, 5 terpenoids and 61 other compounds), 52 of which were identified by comparing with reference standards (including *t*_R_, MS and MS/MS fragments), and the others were identified by searching the mzCloud and PubChem database (including MS and MS/MS fragments). See Table S9 for details. Among them, **Comp**. 77 kaempferol displays antimicrobial, anti‐inflammatory, antioxidant, antitumor, cardioprotective, neuroprotective and antidiabetic activities [41, 42, 43], **Comp**. 106 rutin exerts anticancer activity, antioxidant, anti‐inflammatory and immune regulation properties [44, 45, 46] and so forth.

By HS–GC–MS/MS analysis, a total of 78 volatile compounds were detected and identified by searching the NIST 20 (matching score >80%) and referring to relevant literature [3, 4, 6, 9, 10, 11]. The types of compounds detected by GC–MS include 40 terpenoids, 3 aldehydes, 6 alcohols, 1 ester, 2 ketones and 26 other compounds, and more details can be seen in Table S10, which contains many of the signature compounds in SR that have been reported in the literature [2, 3, 4, 5, 6, 7, 8], such as bornyl formate, camphor and borneol.

By NMR analysis, a total of 37 compounds, including 2 amino acids, 5 sugars, 2 alcohols, 14 organic acids, 2 polyphenols, 1 alkaloid, 7 flavonoids, 1 terpenoid, 1 aromatic family compound and 2 other compounds, were detected and identified by searching the mzCloud and PubChem database (including *δ*_H_ and *δ*_C_) and referring to relevant literature [1, 2, 8]. See Table S11 and Figure S27 for details. Among them, compounds such as rutin and quercitrin were also detected by UHPLC‐Q‐Orbitrap MS, and the identification results of compounds by the two technologies were mutually confirmed.

As shown in Figures S22 and S23, the MIR spectra provided some functional group information to assist in the identification of compounds. For example, absorption peaks in the 1700–1800 cm^−1^ range represent *ν*_C═O_, which may be provided by compounds such as carvone with C═O in the structure.

### 3.2 Classification of Individual Data Matrices

**FIGURE 2:**
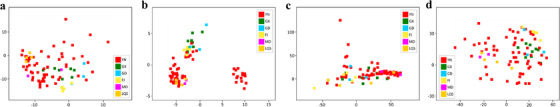
PCA results modelled by UHPLC‐Q‐Orbitrap MS (a), HS–GC–MS/MS (b), NMR (c) and MIR (d) individual data sets (including model set and external validation set). LQS, Lvqiaosha.

The modelling effects of individual data matrices are shown in Figure 2, and there was no separation trend between groups, indicating that the data set constructed by a single analysis technique could not distinguish SR from different origins, so it was necessary to apply data fusion analysis on the collected data sets.

### 3.3 Data Fusion Strategies

**FIGURE 3:**
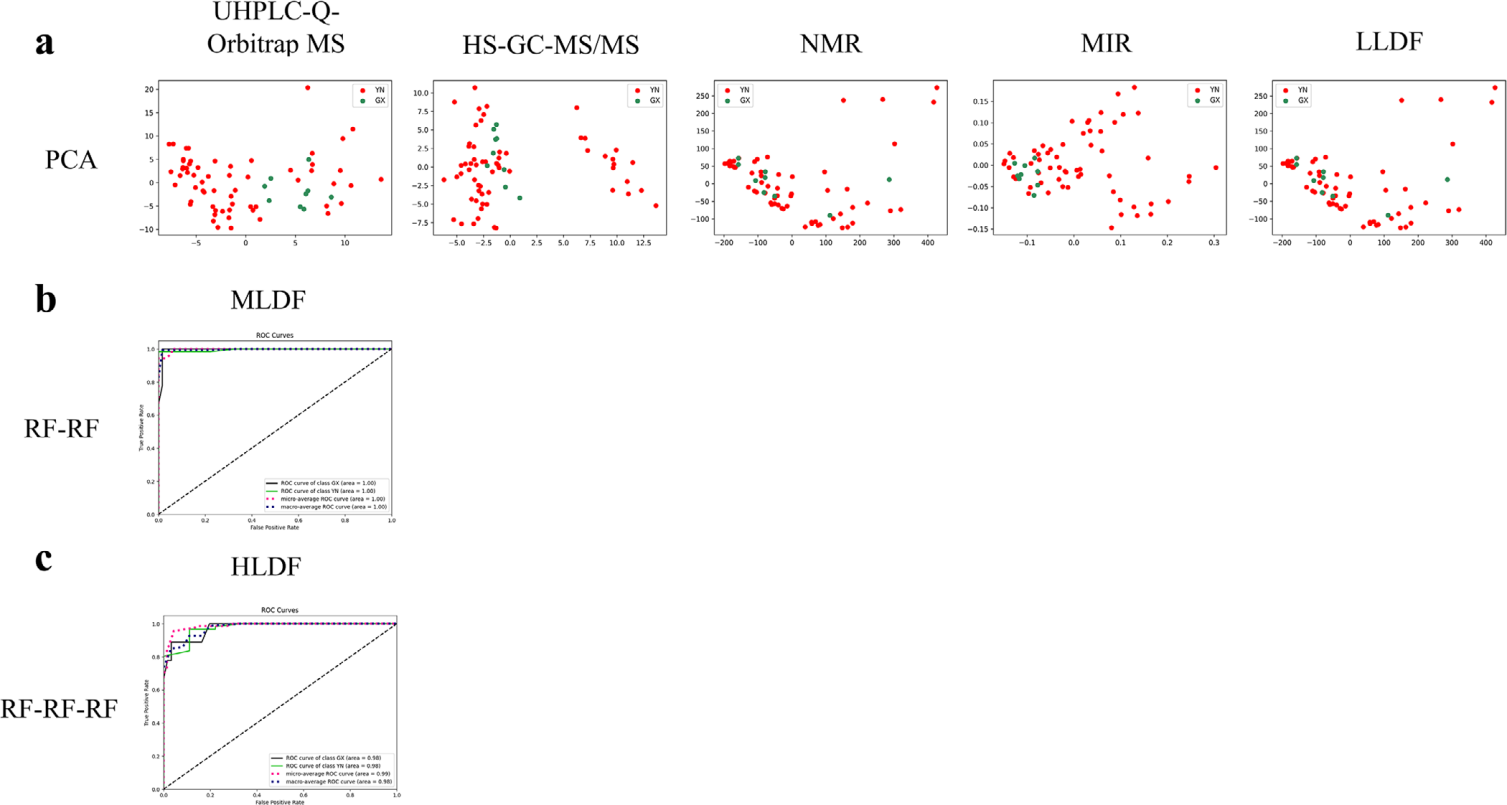
The results of the PCA models (a) established by the single model data sets and LLDF models respectively, (b) modeled using the differential metabolites screened by RF‐RF, (c) established by the differential metabolites screened from RF‐RF‐RF model). HLDF, high‐level data fusion; HS–GC–MS/MS, gas chromatography/triple quadrupole mass spectrometry system coupled with headspace sampler; LLDF, low‐level data fusion; MIR, middle‐infrared; MLDF, mid‐level data fusion; NMR, nuclear magnetic resonance; PCA, principal component analysis; RF, random forest; ROC, receiver operating characteristics; UHPLC‐Q‐Orbitrap MS, ultra‐high‐performance liquid chromatography system coupled with quadrupole‐Orbitrap mass spectrometer.

As shown in Figure 3a, PCA modelling results of the single model data sets showed no significant separation trend between groups. The results of PLS‐DA, SVM, *k*NN, NN, DT and RF models shown in Figure S28 indicate the effects of NN modelled with UHPLC‐Q‐Orbitrap MS and MIR single data set, and *k*NN modelled with MIR single data set were better than others with the AUC values closer to 1.

The modelling effects of LLDF, MLDF and HLDF are shown in Figure 3, Table 1 and Figures S28 and S29.

**TABLE 1:** The results of the low‐level data fusion (LLDF), mid‐level data fusion (MLDF) and high‐level data fusion (HLDF) models.

		OOB	Accuracy train	Accuracy test	Precision score	*f*1 score	Recall score
LLDF	SVM		1.0000	0.8571	0.8571	0.9231	1.0000
	*k*NN		0.8980	0.9524	0.9474	0.9730	1.0000
	NN		1.0000	0.8571	0.8571	0.9231	1.0000
	DT		0.9000	0.8980	0.9524	0.8980	1.0000
	RF	0.9524	0.8571	1.0000	0.9000	0.8571	1.0000
MLDF	PCA–SVM		0.9000	0.9730	1.0000	0.9231	1.0000
	PCA–*k*NN		1.0000	0.9000	0.8980	0.9524	1.0000
	PCA–NN		0.8980	1.0000	0.8571	0.8980	1.0000
	PCA–DT		1.0000	0.9524	0.9730	1.0000	1.0000
	PCA–RF	0.9048	0.9730	1.0000	1.0000	0.9231	1.0000
	PLSDA–SVM		0.9048	0.8571	0.9231	0.9730	1.0000
	PLSDA–*k*NN		1.0000	0.9231	1.0000	0.8980	1.0000
	PLSDA–NN		1.0000	0.8571	0.8571	0.9474	1.0000
	PLSDA–DT		0.9730	1.0000	0.9474	0.9388	1.0000
	PLSDA–RF	0.8571	1.0000	0.8980	1.0000	0.9474	1.0000
	DT–SVM		0.9524	0.9730	0.9000	0.9000	1.0000
	DT–*k*NN		0.9474	1.0000	0.8980	1.0000	1.0000
	DT–NN		1.0000	0.9000	0.9048	0.9231	1.0000
	DT–DT		1.0000	0.9524	0.9474	0.9730	1.0000
	DT–RF	0.9000	1.0000	0.9388	0.8571	0.9000	1.0000
	RF–SVM		0.9000	1.0000	1.0000	0.9524	1.0000
	RF–*k*NN		0.9388	0.9474	0.8571	1.0000	1.0000
	RF–NN		0.9231	0.8571	0.9000	0.8571	1.0000
	RF–DT		1.0000	0.9000	0.9231	0.9730	1.0000
	RF–RF	0.9388	1.0000	0.9048	0.9000	0.9474	1.0000
HLDF	RF–RF–RF	0.8776	1.0000	0.8571	0.8571	0.9231	1.0000

Abbreviations: DT, decision tree; *k*NN, *k* nearest neighbour; NN, neural network; OOB, out‐of‐bag error; PCA, principal component analysis; RF, random forest; SVM, support vector machine.

#### 3.3.1 LLDF

PCA, PLS‐DA, SVM, *k*NN, NN, DT and RF were used to build models separately for the four single data sets and the directly concatenated data set for LLDF. As shown in Figure 3a and Figure S28, PCA and PLS‐DA models offered no clear separation between the two groups. According to Table 1, there was not much difference between these values of OOB, accuracy train, accuracy test, precision score, *f*1 score and recall score obtained by different LLDF models. The values of AUC in SVM and RF models were very low, suggesting their poor modelling results. By contrast, the effects of *k*NN, NN and DT models were just fine with AUC value approaching 1. But on the whole, the effects of LLDF models were poor and could not meet the classification requirements.

#### 3.3.2 MLDF

After selecting the features by PCA, PLS‐DA, DT and RF, PCs, LVs and the first 40 important variables were combined for MLDF modelling using PCA, PLS‐DA, SVM, *k*NN, NN, DT and RF, respectively. As shown in Figure 3b, Table 1 and Figure S29, the parameters of PCA–DT, PCA–RF, PLSDA–*k*NN, PLSDA–NN, PLSDA–RF, DT–*k*NN, DT–NN, DT–RF, RF–*k*NN and RF–RF models were close to 1, and the scatter plot of the PLSDA–PLSDA model showed an obvious separation trend between the two groups, indicating that the models performed well in classification. Similarly, the effects of PCA–*k*NN, PCA–NN, PLSDA–DT, DT–DT, RF–NN and RF–DT models were not bad, whereas the AUC values of PCA–SVM, PLSDA–SVM, DT–SVM and RF–SVM models were less than 0.5, and there was no separation trend between the two groups in the PCA–PCA scatter plot, indicating that the model effects were poor.

#### 3.3.3 HLDF

The results shown in Figure 3c and Table 1 indicated that all parameters of the RF–RF–RF model, including the AUC value, were close to 1, and the model effect was improved, but compared with the MLDF model, RF–RF–RF had a less effective classification than RF–RF, with the slightly lower parameters.

#### 3.3.4 Analysis of the Differential Compounds

To further verify the quality of the model, it was necessary to use part of the data for external verification [35, 36, 37, 38, 39], and the ratio of the modelling data set to the verification data set was 5:1. The sample data sets were divided into 70 and 14 in advance; in this study, the MLDF and HLDF models with better effect were established using 70 data sets, and the YN and GX groups could be significantly separated.

To obtain differential compounds used to distinguish the two groups, RF was chosen as the algorithm used for modelling. After a series of screenings described in Sections 3.3.1–3.3.3, the RF–RF model was selected for further analysis with good classification effects.

**FIGURE 4:**
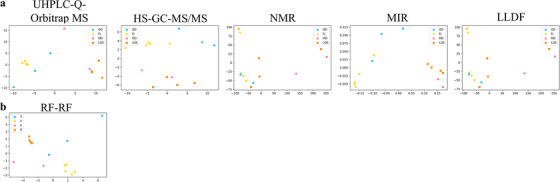
PCA results of classification between GD (3), FJ (4), MD (5) and LQS (6) groups (a), modelled by four single data sets and LLDF, respectively; b, modelled using the differential metabolites screened by RF–RF). HS–GC–MS/MS, gas chromatography/triple quadrupole mass spectrometry system coupled with headspace sampler; LQS, Lvqiaosha; MIR, middle‐infrared; NMR, nuclear magnetic resonance; RF, random forest; UHPLC‐Q‐Orbitrap MS, ultra‐high‐performance liquid chromatography system coupled with quadrupole‐Orbitrap mass spectrometer.

The remaining 14 samples were used for external verification and divided into four groups according to their origins and species (GD, FJ, MD and LQS, as shown in Table S1). Four single data sets were separately analysed by PCA, and the classifications between the four groups were not good. The effect of LLDF by PCA analysis was also poor (as shown in Figure 4a).

The top 38 variables with the highest classification contribution value were screened from the best model (RF–RF) to analyse the remaining 14 data sets during external validation. As shown in Figure 4b, compared with the poor model classification effects of the single data set and LLDF, simple PCA analysis just using 38 selected variables could significantly improve the classification trend of GD, FJ, MD and LQS. The first 38 characteristic variables (including 27 differential metabolites detected by UHPLC‐Q‐Orbitrap MS, HS–GC–MS/MS, NMR and their functional group information obtained by MIR) are shown in Table 2. The MS/MS spectra of the key differential metabolites (24 differential metabolites detected by UHPLC‐Q‐Orbitrap MS and HS–GC–MS/MS) are shown in Figure S30 for verification.

**TABLE 2:** The characteristic variables used to distinguish YN and GX groups screened by RF–RF.

Nos.	Importance (Gini values)	Variable
1	0.066296	*Trans*‐sabinene hydrate (detected by HS–GC–MS/MS, **No**. 16, as shown in Table S4)
2	0.049639	9‐Hpode (detected by UHPLC‐Q‐Orbitrap MS, **Comp**. 148, as shown in Table S3)
3	0.040539	l‐Pyroglutamic acid (UHPLC‐Q‐Orbitrap MS, **Comp**. 29)
4	0.036166	2,3‐Dihydroxy‐3‐methylbutanoic acid (UHPLC‐Q‐Orbitrap MS, **Comp**. 24)
5	0.033329	Pyridostigmine (UHPLC‐Q‐Orbitrap MS, **Comp**. 49)
6	0.033007	γ‐Terpineol (HS–GC–MS/MS, **No**. 31)
7	0.029183	β‐pinene (HS–GC–MS/MS, **No**. 7)
8	0.023957	Benzaldehyde (HS–GC–MS/MS, **No**. 5)
9	0.018061	l‐Histidine (detected by NMR, **No**. 1, *δ*_H_ = 7.89 ppm, as shown in Table S5)
10	0.015950	*ν*_C=O_ (Detected by MIR, wavelength = 1793.474 nm, as shown in Figure S23)
11	0.015780	Quercitrin (NMR, **No**. 37, δ_H_ = 9.37 ppm)
12	0.014592	3‐*O*‐Methyl‐a‐methyldopa (UHPLC‐Q‐Orbitrap MS, **Comp**. 89)
13	0.014428	3‐Amino‐4‐hydroxybenzoic acid (UHPLC‐Q‐Orbitrap MS, **Comp**. 41)
14	0.013638	3,5‐Dihydroxybenzoic acid (UHPLC‐Q‐Orbitrap MS, **Comp**. 101)
15	0.013050	Ylangene (HS–GC–MS/MS, **No**. 41)
16	0.013050	Theophylline (NMR, **No**. 10, *δ*_H_ = 3.36 ppm)
17	0.013050	*β*_C–H_ (MIR, wavelength = 1461.778 nm)
18	0.013000	*ν*_C–O(H)_ (MIR, wavelength = 1180.222 nm)
19	0.012971	d‐Proline (UHPLC‐Q‐Orbitrap MS, **Comp**. 9)
20	0.012741	(*E,E*)‐2,4‐Heptadienal (UHPLC‐Q‐Orbitrap MS, **Comp**. 111)
21	0.012609	*β*_C–H_ (MIR, wavelength = 1488.776 nm)
22	0.012484	Benzene,1‐methyl‐4‐(1‐methylethenyl) (HS–GC–MS/MS, **No**. 27)
23	0.011748	2,3‐Dihydrobenzofuran‐2‐carboxylic acid (UHPLC‐Q‐Orbitrap MS, **Comp**. 107)
24	0.010593	Carvone (UHPLC‐Q‐Orbitrap MS, **Comp**. 131)
25	0.010101	Methoxyeugenol (UHPLC‐Q‐Orbitrap MS, **Comp**. 119)
26	0.009899	d‐2‐Hydroxyglutaric acid (UHPLC‐Q‐Orbitrap MS, **Comp**. 14)
27	0.009792	*γ*_C–H_ (MIR, wavelength = 572.7546 nm)
28	0.009722	*ν*_C=O_ (MIR, wavelength = 1671.981 nm)
29	0.009722	*ν*_C–O(H)_ (MIR, wavelength = 1058.728 nm)
30	0.009706	*ν*_C=C_ (MIR, wavelength = 1654.625 nm)
31	0.009641	Myristicin (UHPLC‐Q‐Orbitrap MS, **Comp**. 134)
32	0.009641	α‐Eleostearic acid (UHPLC‐Q‐Orbitrap MS, **Comp**. 159)
33	0.009348	*ν*_C=C_ (MIR, wavelength = 1648.839 nm)
34	0.009333	7‐Hydroxy‐6‐methyl‐2H‐1‐benzopyran‐2‐one (UHPLC‐Q‐Orbitrap MS, **Comp**. 113)
35	0.009170	Genistein (UHPLC‐Q‐Orbitrap MS, **Comp**. 151)
36	0.008889	*γ*_C–H_ (MIR, wavelength = 773.3152 nm)
37	0.008485	*Trans*‐carveol (HS–GC–MS/MS, **No**. 32)
38	0.008352	Quercitrin (NMR, **No**. 37, *δ*_H_ = 9.76 ppm)

Abbreviations: HS–GC–MS/MS, gas chromatography/triple quadrupole mass spectrometry system coupled with headspace sampler; MIR, middle‐infrared; NMR, nuclear magnetic resonance; UHPLC‐Q‐Orbitrap MS, ultra‐high‐performance liquid chromatography system coupled with quadrupole‐Orbitrap mass spectrometer.

Although the differences between the four groups were obvious, the MD and LQS groups had poor aggregation and large differences within the groups, which may be related to the small number of samples, the difference in harvest times and the unclear specific sources of MD.

### 3.4 Discussion

For SR sample selection, due to the low natural seed setting rate and obvious regional growth conditions, artificial planting has been carried out in YN in recent years to increase its yield. At present, YN has become the main producing area of YCS, and the output accounts for more than 70% of the whole of China with good quality and high medicinal value. In this study, given the low yield of SR in GD and single variety, the sample size was small, and HNS was not included in the research scope with the low component content of TCM and market supply [4, 6].

For UHPLC‐Q‐Orbitrap MS, considering all factors comprehensively (as mentioned in Section 2.3.1), the sample concentration was just 50 mg mL^−1^ and extracted by 50% methanol, but it was inevitable that some compounds similar to quercitrin could not be detected due to its poor solubility in water and low content. However, for NMR, the sample concentration could be as high as 500 mg mL^−1^ extracted by pure methanol. Therefore, in this study, compounds such as isorhamnetin and quercitrin were detected by NMR but not by UHPLC‐Q‐Orbitrap MS. This phenomenon also confirmed the benefits of using the data fusion strategy to comprehensively analyse data from different sources. In addition, several volatile compounds such as carvone, camphor and *p*‐cymene were detected by UHPLC‐Q‐Orbitrap MS, which may be due to their easy solubility in 50% methanol in liquid state and not completely volatilized during extraction. These substances were also detected as volatile compounds in HS–GC–MS/MS, which laterally confirmed the accuracy of the detection results.

Sections 3.2 and 3.3.1 results showed that the model classification effects of both single data set and LLDF were poor, which meant that the expected effects could not be obtained by analysing only the data collected by a single technology or by directly connecting the data collected by four technologies for analysis. Then MLDF and HLDF strategies were attempted. Sections 3.3.2 and 3.3.3 results showed that the model effects of both HLDF and MLDF had been significantly improved, and the effect of MLDF was better than HLDF.

PLS‐DA, as a supervised analysis method, could provide an effective inter‐group separation trend for the modelling data set. However, it was impossible to obtain the differential compounds by PLS‐DA, so this algorithm was not considered for subsequent analysis in this study.

For model selection, the RF model provided characteristic variables simply and directly with a better classification effect than others in this study. Therefore, RF was finally utilized to analyse all the data collected from different platforms with these advantages.

Among the 38 characteristic variables shown in Table 2, 27 differential metabolites were identified by UHPLC‐Q‐Orbitrap MS, HS–GC–MS/MS and NMR techniques, including one alkaloid, two flavonoids, three amino acids, five terpenoids and sixteen other compounds, among which **No**. 3 l‐pyroglutamic acid possesses anxiolytic activity [47], **No**. 6 γ‐terpineol shows antinociceptive effect [48], **No**. 7 β‐pinene has antifungal and antidepressant‐like activity [49, 50], and **No**. 11 quercitrin has anti‐inflammatory, antioxidant and bone protection properties [51, 52], and so forth. In addition, according to Table S11, both **Nos**. 11 and 38 were quercitrin detected by NMR (**No**. 37), and it had peaks at *δ*_H_ = 9.37 and 9.76 ppm at the same time, so the phenomenon of one compound providing two characteristic variables was appeared, which indicates quercitrin may be one of the main compounds that cause the difference in SR from different sources. Meanwhile, the functional group information obtained by MIR technology could support the structure of the compounds, among which the carbonyl group with a wavelength of 1793.474 nm at **No**. 10 may be provided by carvone at **No**. 24, the tertiary alcohol group with a wavelength of 1180.222 nm at **No**. 18 may be provided by γ‐terpineol at **No**. 6, the secondary alcohol group of **No**. 29 wavelength 1058.728 nm may be provided by *trans*‐carveol of **No**. 37, and the remaining C–H bonds and carbonyl groups may be provided by other compounds in Table 2.

In the process of model building, the 38 variables with the greatest contribution value to the obvious separation of YN and GD groups were obtained through continuous training. Only a simple PCA analysis using these 38 variables was performed on the verification set, and the four completely different groups in the verification set could be clearly separated. This meant there was a large degree of overlap in the variables (differential compounds) that caused differences between groups of SRs from different sources. In future studies, through random analysis of differences between any two groups, some variables that cause differences between other groups can be obtained, which greatly reduces the workload of data analysis. For the application of the results, further studies are needed to expand the sample size, collect more samples from different batches, varieties and origins and consider the effect of harvest years and storage duration on metabolites.

## 4 Conclusion

In this study, 84 SR samples from different origins and species were analysed. The various substances contained in SR (a total of 286 compounds) and their functional group information were comprehensively characterized by UHPLC‐Q‐Orbitrap MS, HS–GC–MS/MS, NMR and MIR technologies. Among all 84 samples, 70 samples of individuals from each sampling site (SRs from YN and GX) were selected for the model establishment, and the remaining 14 samples (SRs from GD, FJ, MD and LQS) were used for external verification. After a series of optimizations of the data fusion models, including PCA, PLS‐DA, SVM, *k*NN, NN, DT and RF, the RF–RF model was selected with a better classification performance. After combining the features selected by RF, the RF model was established to distinguish the YN and GX groups, and the 27 differential compounds (including flavonoids, polyphenols and terpenoids) and their functional group information were screened, which could classify the four groups (GD, FJ, MD and LQS) in the validation set at the same time. A more comprehensive and accurate means of analysis was found.

## Author Contributions

**Yuxin Zhang**: writing – original draft, writing – review and editing, methodology, investigation, validation, visualization, data curation, formal analysis. **Yihang Li**: writing – review and editing, supervision. **Ze Li**: writing – review and editing, supervision. **Zhonglian Zhang**: writing – review and editing, resources, supervision, funding acquisition. **Yue Zhang**: writing – review and editing, supervision. **Biying Chen**: writing – review and editing, software, supervision, investigation, methodology. **Lixia Zhang**: writing – review and editing, supervision. **Meifang Song**: writing – review and editing, supervision. **Miaomiao Jiang**: writing – review and editing, conceptualization, funding acquisition, project administration, resources, investigation.

## Conflicts of Interest

The authors declare no conflicts of interest.

## Supporting information




**Supplementary File 1**: ansa70029‐sup‐0001‐SuppMat.docx.

## Data Availability

The authors have nothing to report.
