# Supplementary material for: Comprehensive Discrimination of Amomi Fructus From Different Origins Using UHPLC‐Q‐Orbitrap MS, HS–GC–MS/MS, NMR and MIR Technologies Based On Data Fusion Strategies
Source: Anal Sci Adv. 2025 Jul 27;6(2):e70029. doi: 10.1002/ansa.70029 (PMC12296717; doi:10.1002/ansa.70029)
Supplement: Supplementary file 1 — ansa70029‐sup‐0001‐SuppMat.docx. [file ANSA-6-e70029-s001.docx]

**Supplementary information**

**Comprehensive discrimination of Amomi Fructus from different origins using UHPLC-Q-Orbitrap MS, HS-GC-MS/MS, NMR, and MIR technologies based on data fusion strategies**

Yuxin Zhang^1,#^, Yihang Li^2,#^, Ze Li^1^, Zhonglian Zhang^2^, Yue Zhang^2^, Biying Chen^1^, Lixia Zhang^2^, Meifang Song^2,*^, Miaomiao Jiang^1,*^

^1^ State Key Laboratory of Component-based Chinese Medicine, Tianjin Key Laboratory of TCM Chemistry and Analysis, Haihe Laboratory of Modern Chinese Medicine, Tianjin University of Traditional Chinese Medicine, 10 Poyanghu Road, Jinghai district, Tianjin, 301617, P. R. China

^2^ Yunnan Key Laboratory of Southern Medicine Utilization, Yunnan Branch of Institute of Medicinal Plant Development, Chinese Academy of Medical Sciences, Peking Union Medical College, Jinghong, 666100, China

#: These two authors contributed equally to this work and should be considered as co-first authors.

*: Corresponding authors, E-mail addresses: [songmeifang1981@126.com](mailto:songmeifang1981@126.com) (Meifang Song); [miaomiaojiang@126.com](mailto:miaomiaojiang@126.com) (Miaomiao Jiang)

1S. SR samples information

Eighty-four SR samples were harvested from different origins and authenticated by Professor Zhonglian Zhang from Yunnan Branch of Institute of Medicinal Plant Development, Chinese Academy of Medical Sciences, the batch numbers and detailed information were shown in Table 1S.1.

**Table 1S.1** Batch numbers and detailed information of SR samples

| **No.** | **Batch**  **No.** | **Harvest**  **time** | **Origin** | **Longitude** | **Latitude** | **Variety** | **Abbreviation** |
| --- | --- | --- | --- | --- | --- | --- | --- |
| 1 | XBS1 | 2022 | Guangxi, China | E106°39′57.5″ | N23°3′56.7″ | *Amomum villosum* Lour. | GX |
| 2 | XCS4 | 2019 | Guangxi, China | E107°20′28.6″ | N22°49′6.76″ | *Amomum villosum* Lour. | GX |
| 3 | XCS3 | 2019 | Guangxi, China | E107°18′55.9″ | N21°49′9.27″ | *Amomum villosum* Lour. | GX |
| 4 | XCS6 | 2019 | Guangxi, China | E107°22′51.5″ | N21°48′52.9″ | *Amomum villosum* Lour. | GX |
| 5 | XCS2 | 2019 | Guangxi, China | E107°8′52.6″ | N21°59′46.2″ | *Amomum villosum* Lour. | GX |
| 6 | XCS1 | 2019 | Guangxi, China | E107°8′52.6″ | N21°59′46.2″ | *Amomum villosum* Lour. | GX |
| 7 | XCS5 | 2019 | Guangxi, China | E106°51′27.0″ | N22°56′42.9″ | *Amomum villosum* Lour. | GX |
| 8 | XNS2 | 2019 | Guangxi, China | E107°38′36.3″ | N22°55′21.4″ | *Amomum villosum* Lour. | GX |
| 9 | XNS1 | 2019 | Guangxi, China | E107°53′0.92″ | N23°19′22.6″ | *Amomum villosum* Lour. | GX |
| 10 | YHS1 | 2019 | Yunnan, China | E103°17′28.9″ | N22°59′30.0″ | *Amomum villosum* Lour. | YN |
| 11 | YHS2 | 2019 | Yunnan, China | E103°19′44.1″ | N22°55′12.2″ | *Amomum villosum* Lour. | YN |
| 12 | YHS3 | 2019 | Yunnan, China | E103°13′2.30′′ | N22°50′25.3′′ | *Amomum villosum* Lour. | YN |
| 13 | YHS4 | 2019 | Yunnan, China | E103°13′2.30′′ | N22°50′25.3′′ | *Amomum villosum* Lour. | YN |
| 14 | YHS5 | 2019 | Yunnan, China | E103°17′28.9″ | N22°59′30.0″ | *Amomum villosum* Lour. | YN |
| 15 | YHS6 | 2022 | Yunnan, China | E103°19′44.1′′ | N22°55′12.2′′ | *Amomum villosum* Lour. | YN |
| 16 | YHS7 | 2022 | Yunnan, China | E103°13′8.94′′ | N22°58′20.1′′ | *Amomum villosum* Lour. | YN |
| 17 | YHS8 | 2022 | Yunnan, China | E103°17′28.9″ | N22°59′30.0″ | *Amomum villosum* Lour. | YN |
| 18 | YHS9 | 2022 | Yunnan, China | E103°31′35.9″ | N22°46′15.5″ | *Amomum villosum* Lour. | YN |
| 19 | YHS10 | 2022 | Yunnan, China | E103°19′8.76″ | N22°56′23.4″ | *Amomum villosum* Lour. | YN |
| 20 | YHS11 | 2022 | Yunnan, China | E103°30′44.0″ | N23°4′6.97″ | *Amomum villosum* Lour. | YN |
| 21 | YPS1 | 2022 | Yunnan, China | E102°15′56′′ | N22°34′14.3′′ | *Amomum villosum* Lour. | YN |
| 22 | YPS2 | 2022 | Yunnan, China | E101°99′42.5′′ | N22°48′53.5′′ | *Amomum villosum* Lour. | YN |
| 23 | YPS3 | 2022 | Yunnan, China | E102°07′9.4′′ | N22°33′49.9′′ | *Amomum villosum* Lour. | YN |
| 24 | YPS5 | 2022 | Yunnan, China | E99°38′45.7″ | N22°30′59.6″ | *Amomum villosum* Lour. | YN |
| 25 | YPS6 | 2022 | Yunnan, China | E99°40′35″ | N22°29′35″ | *Amomum villosum* Lour. | YN |
| 26 | YPS4 | 2022 | Yunnan, China | E99°36′23.8″ | N22°39′40.5″ | *Amomum villosum* Lour. | YN |
| 27 | YWS9 | 2022 | Yunnan, China | E105°44′24.4′′ | N23°39′35.2′′ | *Amomum villosum* Lour. | YN |
| 28 | YWS12 | 2022 | Yunnan, China | E104°47′1.25′′ | N23°14′48.3′′ | *Amomum villosum* Lour. | YN |
| 29 | YWS1 | 2019 | Yunnan, China | E103°57′50.0″ | N22°58′2.81″ | *Amomum villosum* Lour. | YN |
| 30 | YWS2 | 2019 | Yunnan, China | E104°15′16.0″ | N23°23′47.3″ | *Amomum villosum* Lour. | YN |
| 31 | YWS3 | 2019 | Yunnan, China | E103°59′23.4″ | N23°0′53.2″ | *Amomum villosum* Lour. | YN |
| 32 | YWS4 | 2022 | Yunnan, China | E103°59′14.8″ | N22°59′27.0″ | *Amomum villosum* Lour. | YN |
| 33 | YWS5 | 2022 | Yunnan, China | E103°59′28.6″ | N22°44′47.3″ | *Amomum villosum* Lour. | YN |
| 34 | YWS6 | 2022 | Yunnan, China | - | - | *Amomum villosum* Lour. | YN |
| 35 | YWS7 | 2022 | Yunnan, China | - | - | *Amomum villosum* Lour. | YN |
| 36 | YXS3 | 2019 | Yunnan, China | E100°46′28.7″ | N21°46′12.8″ | *Amomum villosum* Lour. | YN |
| 37 | YXS4 | 2019 | Yunnan, China | E100°53′32.4″ | N21°48′37.0″ | *Amomum villosum* Lour. | YN |
| 38 | YXS5 | 2019 | Yunnan, China | E100°53′32.0″ | N21°53′47.8″ | *Amomum villosum* Lour. | YN |
| 39 | YXS6 | 2019 | Yunnan, China | E101°0′40.1″ | N22°2′32.7″ | *Amomum villosum* Lour. | YN |
| 40 | YXS7 | 2019 | Yunnan, China | E101°4′5.66″ | N22°7′28.8″ | *Amomum villosum* Lour. | YN |
| 41 | YXS8 | 2019 | Yunnan, China | E100°45′38.5″ | N22°12′13.4″ | *Amomum villosum* Lour. | YN |
| 42 | YXS9 | 2022 | Yunnan, China | E101°0′40.1″ | N22°2′32.7″ | *Amomum villosum* Lour. | YN |
| 43 | YXS10 | 2022 | Yunnan, China | E100°56′50′′ | N21°46′57′′ | *Amomum villosum* Lour. | YN |
| 44 | YXS11 | 2022 | Yunnan, China | E100°42′26′′ | N21°46′21′′ | *Amomum villosum* Lour. | YN |
| 45 | YXS12 | 2022 | Yunnan, China | E100°45′58.3′′ | N21°40′48′′ | *Amomum villosum* Lour. | YN |
| 46 | YXS13 | 2022 | Yunnan, China | E101°4′52.7′′ | N22°5′50.6′′ | *Amomum villosum* Lour. | YN |
| 47 | YXS14 | 2022 | Yunnan, China | E100°52′16.4′′ | N22°20′3.47′′ | *Amomum villosum* Lour. | YN |
| 48 | YXS15 | 2022 | Yunnan, China | E100°53′46.6′′ | N21°50′29.2′′ | *Amomum villosum* Lour. | YN |
| 49 | YXS1 | 2019 | Yunnan, China | E101°20′7.98″ | N21°18′50″ | *Amomum villosum* Lour. | YN |
| 50 | YXS2 | 2019 | Yunnan, China | E101°32′33.3″ | N21°43′30.1″ | *Amomum villosum* Lour. | YN |
| 51 | YXS16 | 2022 | Yunnan, China | E101°46′34.1′′ | N21°48′46.7′′ | *Amomum villosum* Lour. | YN |
| 52 | YXS17 | 2022 | Yunnan, China | E101°22′11.5′′ | N22°4′22.8′′ | *Amomum villosum* Lour. | YN |
| 53 | YXS18 | 2022 | Yunnan, China | E101°32′42.5′′ | N21°17′8.75′′ | *Amomum villosum* Lour. | YN |
| 54 | YXS19 | 2022 | Yunnan, China | E101°42′31.7″ | N21°14′41.2″ | *Amomum villosum* Lour. | YN |
| 55 | YXS20 | 2022 | Yunnan, China | E101°42′31.7″ | N21°14′41.2″ | *Amomum villosum* Lour. | YN |
| 56 | MGWZZ-10 | 2022 | Yunnan, China | - | - | *Amomum villosum* Lour. | YN |
| 57 | MGWZZ-11 | 2022 | Yunnan, China | - | - | *Amomum villosum* Lour. | YN |
| 58 | MGWZZ-12 | 2022 | Yunnan, China | - | - | *Amomum villosum* Lour. | YN |
| 59 | MGWZZ-15 | 2022 | Yunnan, China | - | - | *Amomum villosum* Lour. | YN |
| 60 | MGWZF-18 | 2022 | Yunnan, China | - | - | *Amomum villosum* Lour. | YN |
| 61 | MGWZF-19 | 2022 | Yunnan, China | - | - | *Amomum villosum* Lour. | YN |
| 62 | MGWZF-29 | 2022 | Yunnan, China | - | - | *Amomum villosum* Lour. | YN |
| 63 | MGWZF-30 | 2022 | Yunnan, China | - | - | *Amomum villosum* Lour. | YN |
| 64 | MGWZF-31 | 2022 | Yunnan, China | - | - | *Amomum villosum* Lour. | YN |
| 65 | MGWZF-33 | 2022 | Yunnan, China | - | - | *Amomum villosum* Lour. | YN |
| 66 | MGWZF-35 | 2022 | Yunnan, China | - | - | *Amomum villosum* Lour. | YN |
| 67 | MGWZF-36 | 2022 | Yunnan, China | - | - | *Amomum villosum* Lour. | YN |
| 68 | MGWZF-38 | 2022 | Yunnan, China | - | - | *Amomum villosum* Lour. | YN |
| 69 | CK-1 | 2022 | Yunnan, China | - | - | *Amomum villosum* Lour. | YN |
| 70 | CK-2 | 2022 | Yunnan, China | - | - | *Amomum villosum* Lour. | YN |
| 71 | FZS1 | 2019 | Fujian, China | E117°28′52.2″ | N24°55′0.12″ | *Amomum villosum* Lour. | FJ |
| 72 | FZS5 | 2019 | Fujian, China | E117°30′32.2″ | N24°55′5.62″ | *Amomum villosum* Lour. | FJ |
| 73 | FZS2 | 2019 | Fujian, China | E117°51′55.4″ | N24°40′56.5″ | *Amomum villosum* Lour. | FJ |
| 74 | FZS3 | 2019 | Fujian, China | E117°52′31.1″ | N24°43′24.4″ | *Amomum villosum* Lour. | FJ |
| 75 | FZS4 | 2019 | Fujian, China | E117°53′17.7″ | N24°43′49.5″ | *Amomum villosum* Lour. | FJ |
| 76 | DMS1 | 2022 | Guangdong, China | E111°15′41.7″ | N22°22′38.9″ | *Amomum villosum* Lour. | GD |
| 77 | DYS1 | 2019 | Guangdong, China | E111°34′53.4″ | N22°15′21.6″ | *Amomum villosum* Lour. | GD |
| 78 | DZS1 | 2022 | Guangdong, China | E112°28′20.5″ | N23°48′23.0″ | *Amomum villosum* Lour. | GD |
| 79 | YXM1 | 2022 | Myanmar | E100°59′3.44′′ | N22°0′55.1′′ | SR from Myanmar | MD |
| 80 | YXM2 | 2022 | Myanmar | - | - | SR from Myanmar | MD |
| 81 | YWS8 | 2022 | Yunnan, China | E105°44′24.4′′ | N23°39′35.2′′ | *Amomum villosum* Lour. var. xanthioides T. L. Wu et Senjen (Lvqiaosha) | LQS |
| 82 | YWS10 | 2022 | Yunnan, China | E105°44′24.4′′ | N23°39′35.2′′ | *Amomum villosum* Lour. var. xanthioides T. L. Wu et Senjen (Lvqiaosha) | LQS |
| 83 | YWS11 | 2022 | Yunnan, China | E105°44′24.4′′ | N23°39′35.2′′ | *Amomum villosum* Lour. var. xanthioides T. L. Wu et Senjen (Lvqiaosha) | LQS |
| 84 | YXL1 | 2022 | Yunnan, China | E100°56′04′′ | N22°03′26.7′′ | *Amomum villosum* Lour. var. xanthioides T. L. Wu et Senjen (Lvqiaosha) | LQS |

2S. Condition optimization for UHPLC-Q-Orbitrap MS analysis

To analyze the compounds in SR more comprehensively and provide a basis for more accurate classification, a QC sample was used to optimize the conditions of UHPLC-Q-Orbitrap MS analysis. As shown in Fig 2S.1, to better observe the peak shape, the total ion chromatogram (TIC) was enlarged and subsequent optimization was performed based on TIC from 1.8 min to 19 min.

The extraction solvents of SR were screened to maximize the detection of the chemical composition by comparing five different solvents, including water, 25% methanol-water (*v/v*), 50% methanol-water (*v/v*), 75% methanol-water (*v/v*), and 100% methanol. The comprehensive determination was made by observing the resolution of the major chromatographic peaks in TIC and the number of chromatographic peaks extracted by SIEVE software. As shown in Figs. 2S.2 and 2S.3, the largest number of peaks could be obtained by using 50% methanol-water (*v/v*) with higher resolution and better peak shape. Finally, 50% methanol was selected as the extraction solvent of SR to extract both hydrophobic and hydrophilic compounds.

To extract the compounds contained in SR to a greater extent and avoid the degradation of heat-unstable substances, the extraction time was optimized, as shown in Figs. 2S.4 and 2S.5. The extraction time of 60 min was chosen for the maximum number of peaks.

Considering the high selectivity of reversed-phase chromatography (RPC) separation and the role of retention time (*t_R_*) in structural elucidation, RPC was selected coupled with MS for the analysis of SR. The screening of the chromatographic stationary phase was mainly carried out by comparing seven C_18_-bonding stationary phases with assessing the overall performance in resolution and selectivity (the number of resolved peaks) extracted using SIEVE software (Figs. 2S.6 and 2S.7). The relevant information on reversed-phase columns is shown in Table 2S.1. According to the results, the HSS T_3_ column was finally selected as the chromatographic stationary phase for possessing better peak capacity than others with higher resolution, higher response, and better peak shape.

The chromatographic separation conditions, which included the mobile phase, column temperature, flow rate, and elution gradient, were also optimized.

The pH value of the mobile phase would improve the peak shape and prevent tailing universally. Therefore, a small amount of organic acid was often added to the aqueous phase. For elution, acetonitrile (CH_3_CN) was selected as the organic phase with the advantages of high separation resolution, strong elution ability, strong solubility, low viscosity, and low system background pressure. As shown in Figs. 2S.8-2S.11, when CH_3_CN-0.1% HOAc was used as the mobile phase, although the number of ion chromatographic peaks extracted by SIEVE software was relatively larger, the chromatographic peak shape was poor and the response of the impurity increased. Whereas, when FA was used with a concentration of 0.1% in the aqueous phase, the resolution of chromatographic peaks was higher and the peak shape was better, hence, it was selected as the aqueous phase for further analysis.

To obtain the best resolution and response, the elution gradient was optimized and set as mentioned in “2.3.1”.

The column temperature and the flow rate were compared. As shown in Figs. 2S.12-2S.15, the column temperature was set as 35 °C, and the flow rate was set as 0.30 mL·min^-1^ with the largest number of extracted ion chromatographic peaks and the best peak resolution.

To enable the highly sensitive detection of SR components, key ESI source parameters in the positive ESI mode (covering most subclasses of compounds), mainly including SV (2.0-4.0 kV), CT (280-360 ℃), and AGHT (250-450 °C), were optimized in sequence. The main components of SR, representing different structure subclasses, were selected as quantitative indicators, namely Comp. **52**, cinnamic acid (organic acid); Comp. **70**, D-tryptophan (amino acids); Comp. **122**, berberine (alkaloids); Comp. **78**, kaempferol (flavonoids); Comp. **91**, catechin (polyphenols); Comp. **132**, caryophyllene oxide (terpenoids); and Comp. **56**, pantothenic acid (other compounds). According to the corresponding peak areas of the seven types of components under different source parameter settings (as shown in Fig. 2S.16), the optimal mass spectrometry parameters were determined as mentioned in “2.3.1”.

To provide more available structural information for characterizing compounds of SR, NCE was also optimized in positive mode. NCE could set three different collision energies to cover the collision energy required by most different types of compounds and provide more useful structural information for the qualitative identification of SR. Four normalized collision energies were set for comparison, including 10/20/30%, 10/20/40%, 10/30/50%, and 20/40/60%.

Seven main index components of SR (as mentioned above) were used to observe MS^2^ fragmentation behaviors to identify which best showed the diversity of product ions. All types of compounds produced characteristic fragments at different energies, and the abundance of parent ions decreased with increasing collision energy (Fig. 2S.17). For most index compounds, the best NCE was 20/40/60%.


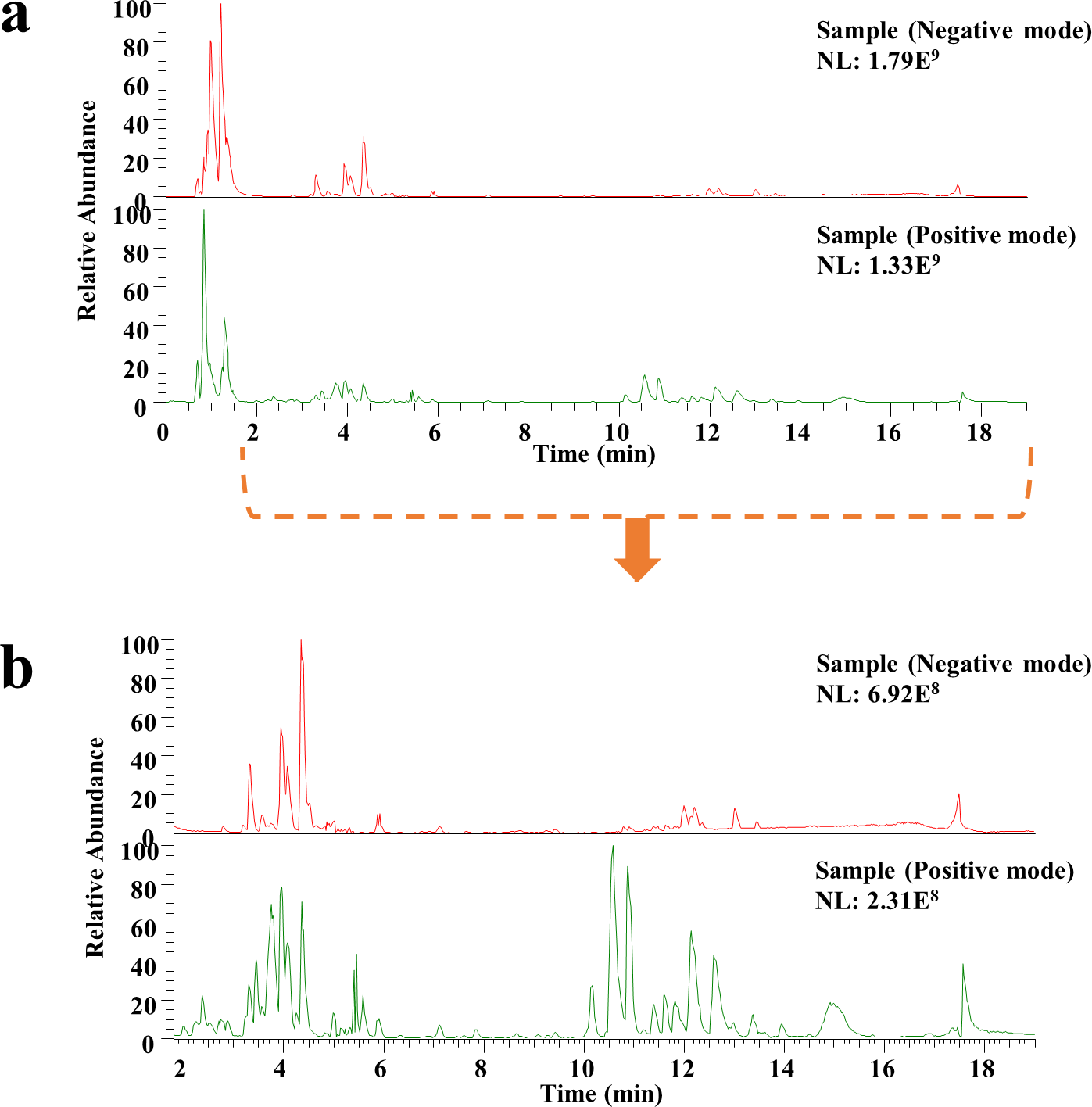


**Fig. 2S.1** Total ion chromatogram of SR sample from 0 to 19 min (a) and from 1.8 to 19 min (b)


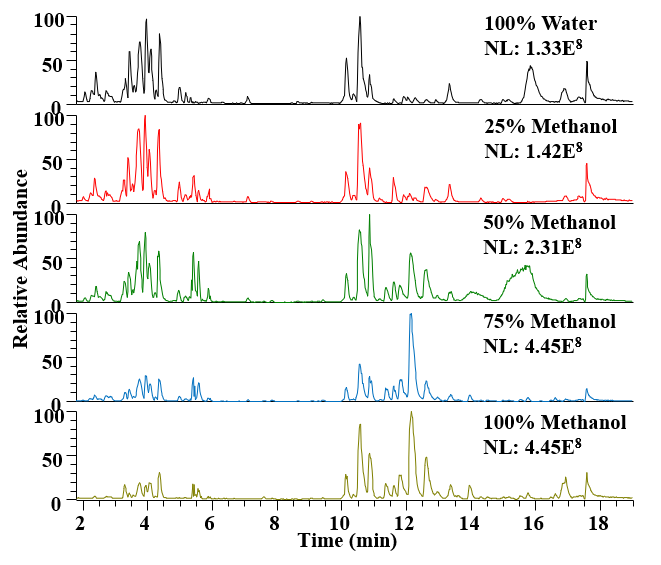


**Fig. 2S.2** Total ion chromatogram of separation of components from SR by five solvents (1.8-19 min)


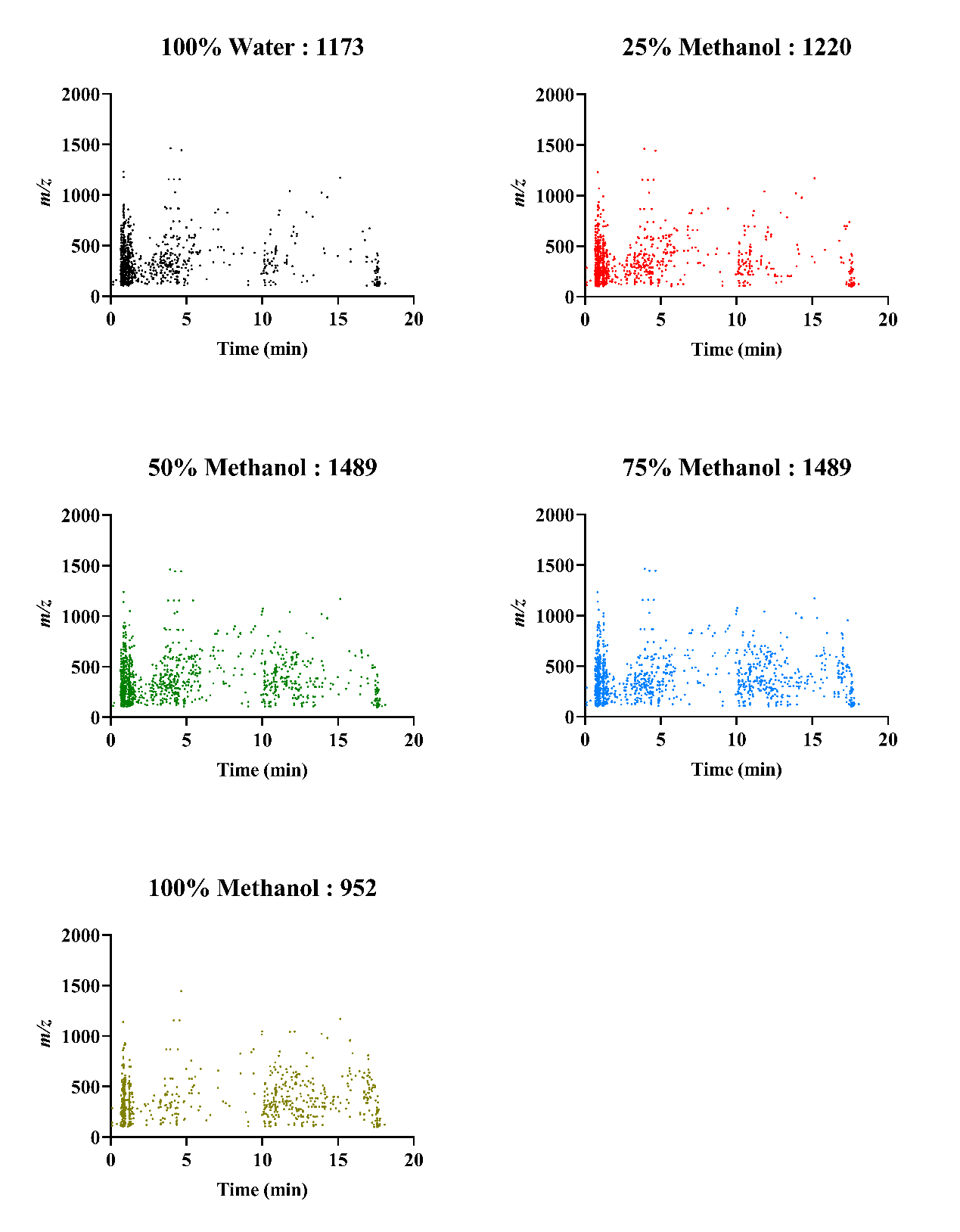


**Fig. 2S.3** Comparison of the number of peaks detected by the separation of five solvents (0-19 min)


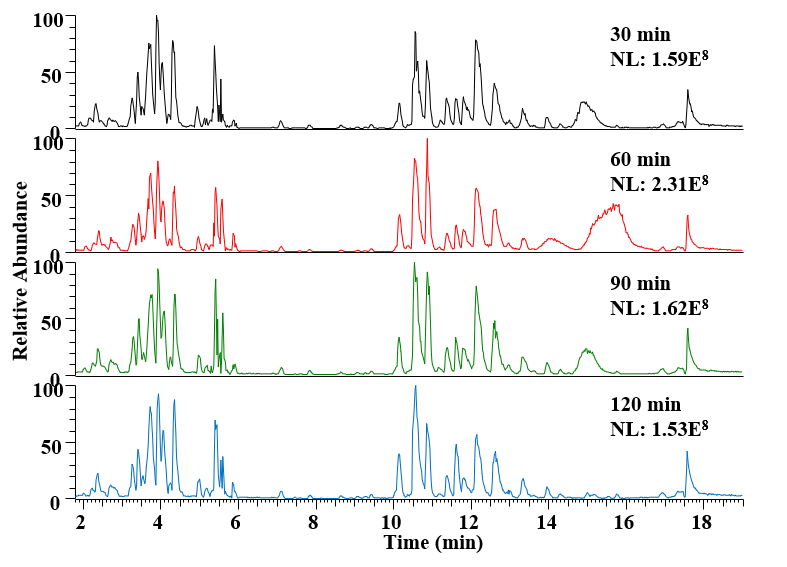


**Fig. 2S.4** Total ion chromatogram of separation of components from SR at four different extraction times (1.8-19 min)


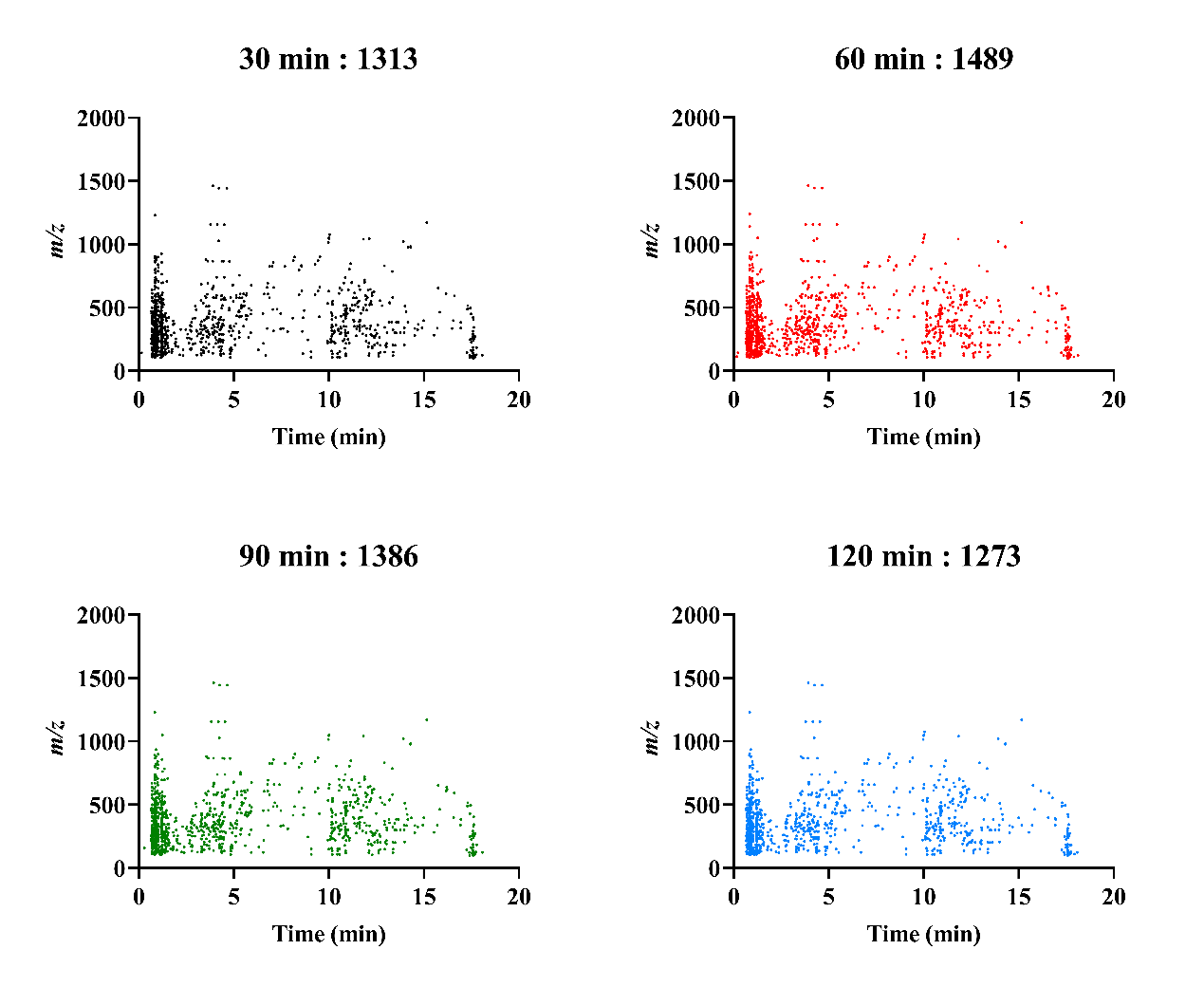


**Fig. 2S.5** Comparison of the number of peaks detected by the separation of four different extraction times (0-19 min)


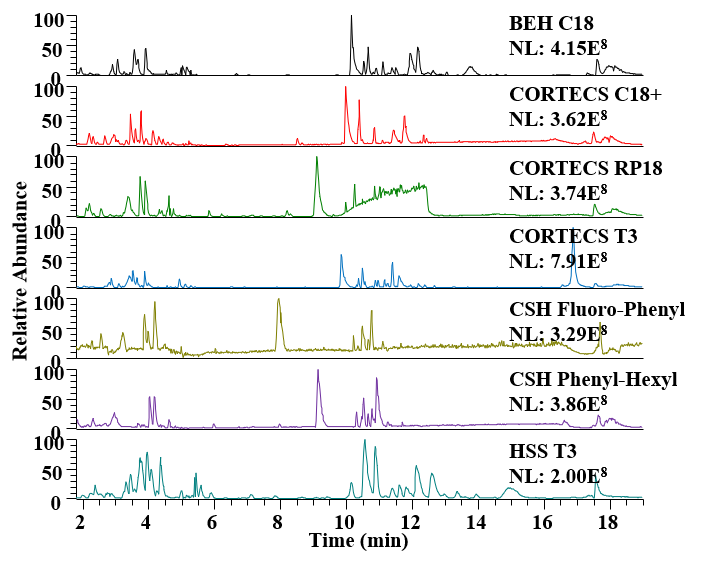


**Fig. 2S.6** Total ion chromatogram of separation of components from SR by seven reversed-phase columns (1.8-19 min)


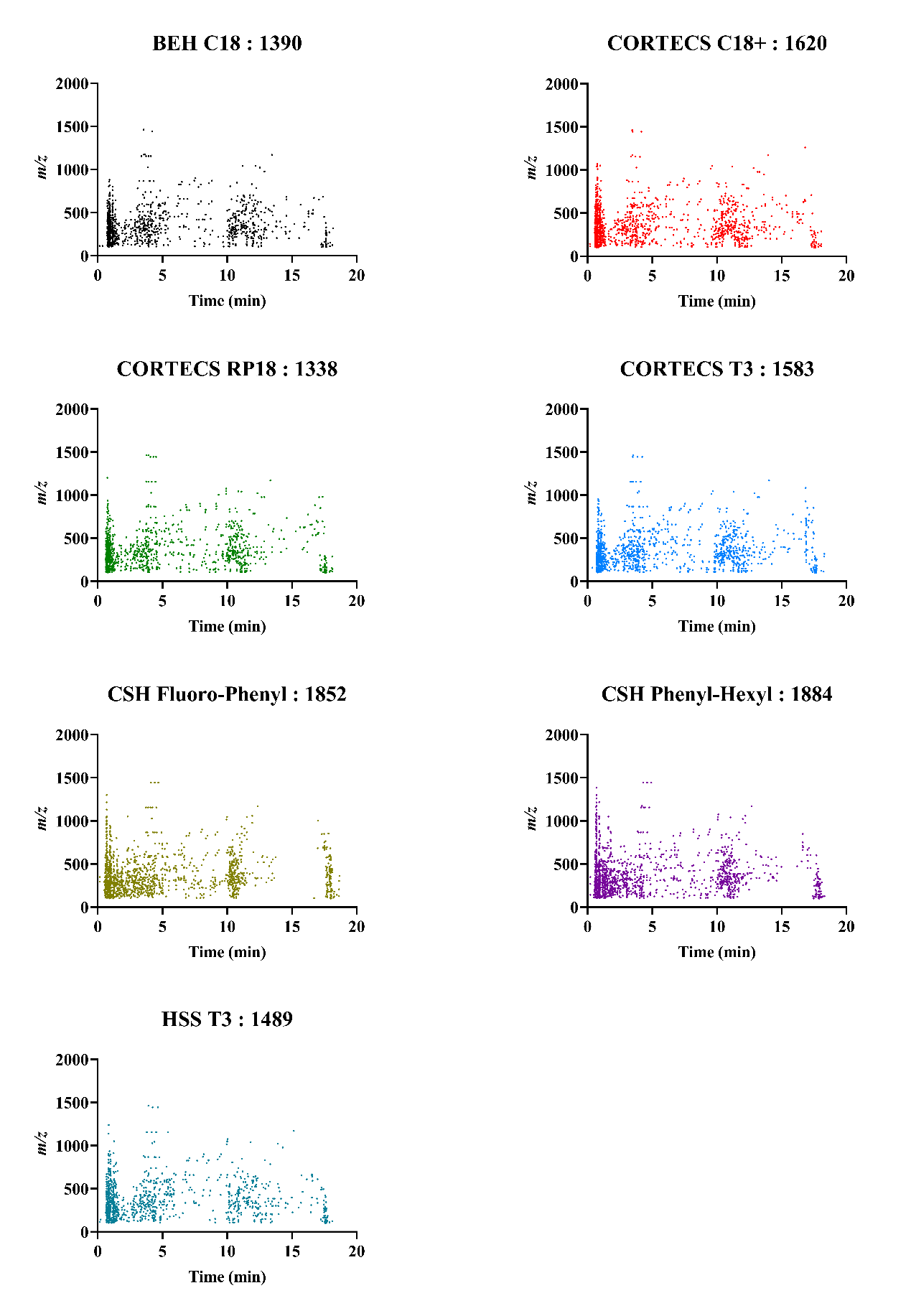


**Fig. 2S.7** Comparison of the number of peaks detected by the separation of seven reversed-phase columns (0-19 min)

**Table 2S.1** Information of seven alternative columns

| Type | Specification / Manufacturer | Property |
| --- | --- | --- |
| BEH C_18_ | 1.7 μm, 2.1×100 mm, Waters | Ethylene bridged hybrid: BEH; suitable for retention of moderate or weakly polar compounds; wide tolerance to pH |
| CORTECS T_3_ | 1.6 μm, 2.1×100 mm, Waters | Compatible with 100% aqueous mobile phases, and provides excellent retention for polar and non-polar analytes. The particle morphology offers high efficnecy and lower backpressure |
| HSS T_3_ | 1.8 μm, 2.1×100 mm, Waters | High-strength silica: HSS; suitable for enhanced retention of polar compounds; compatible with 100% aqueous phase |
| CORTECS C_18+_ | 1.6 μm, 2.1×100 mm, Waters | General purpose, high-efficiency C_18_ columns based on a solid-core particle; designed to deliver excellent peak shape for basic analytes at low pH |
| CORTECS Shield RP_18_ | 1.6 μm, 2.1×100 mm, Waters | Provides complimentary selectivity to C_8_ and C_18_ stationary phases; the embedded carbamate group in the bonded phase ligand provides alternate selectivity; the solid core particle morphology offers high efficiency and low backpressures |
| CSH^TM^ Fluoro Phenyl | 1.7 μm, 2.1×100 mm, Waters | Provides maximum method development freedom, especially when using low pH mobile phases |
| CSH^TM^ Phenyl Hexyl | 1.7 μm, 2.1×100 mm, Waters | Provides alternative analyte selectivity and are a valuable tool for method development |


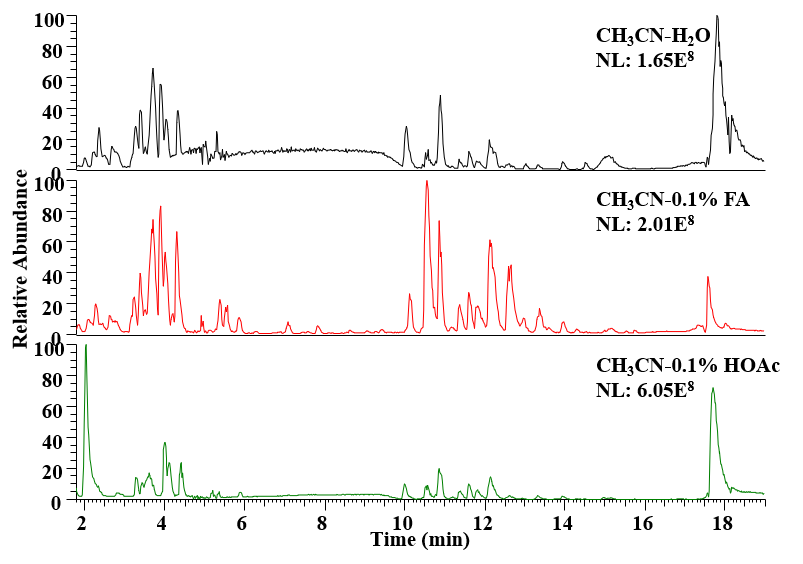


**Fig. 2S.8** Total ion chromatogram of separation of components from SR by different aqueous phase additives (1.8-19 min)


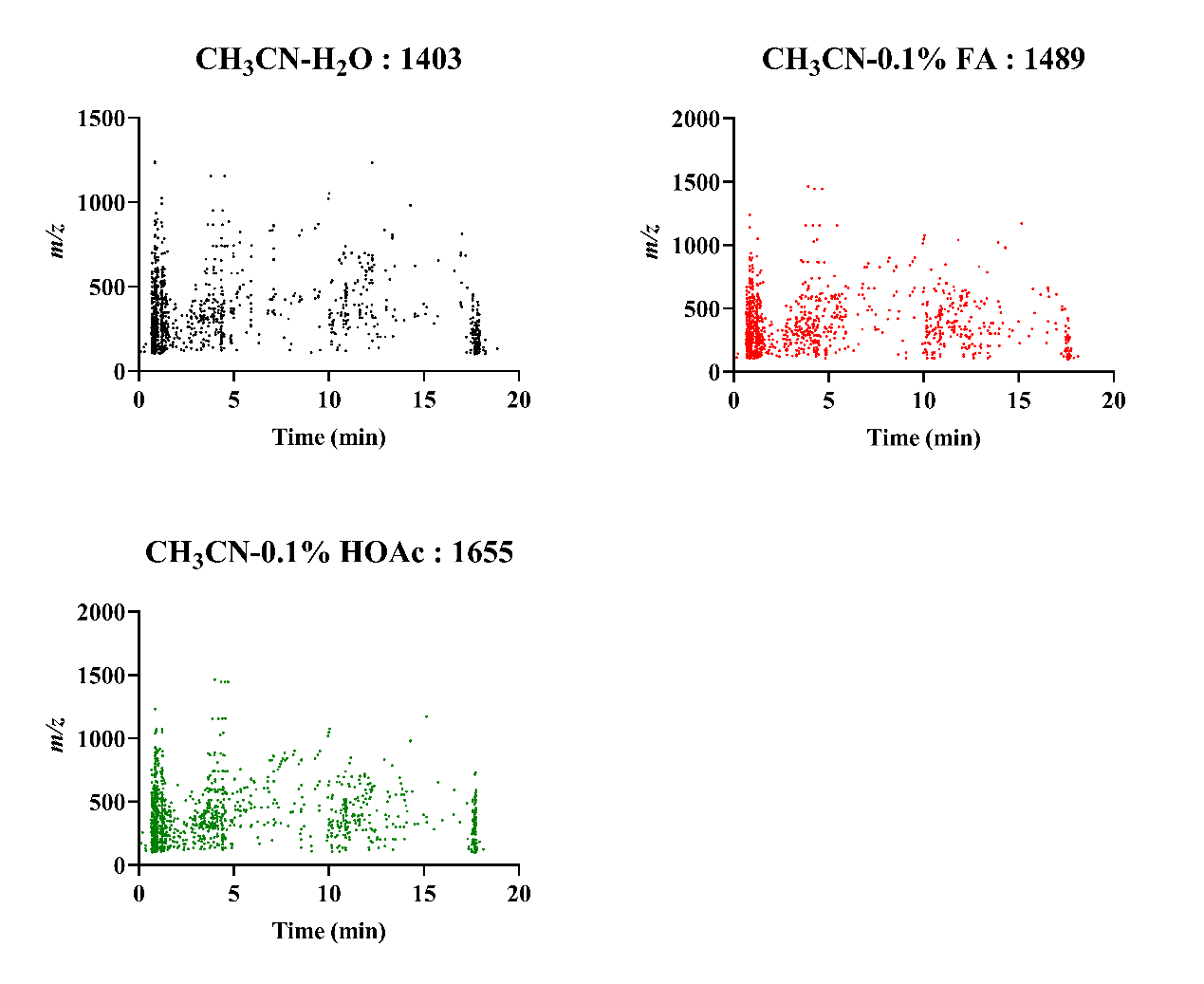


**Fig. 2S.9** Comparison of the number of peaks in ion chromatography with different aqueous phase additives (0-19 min)


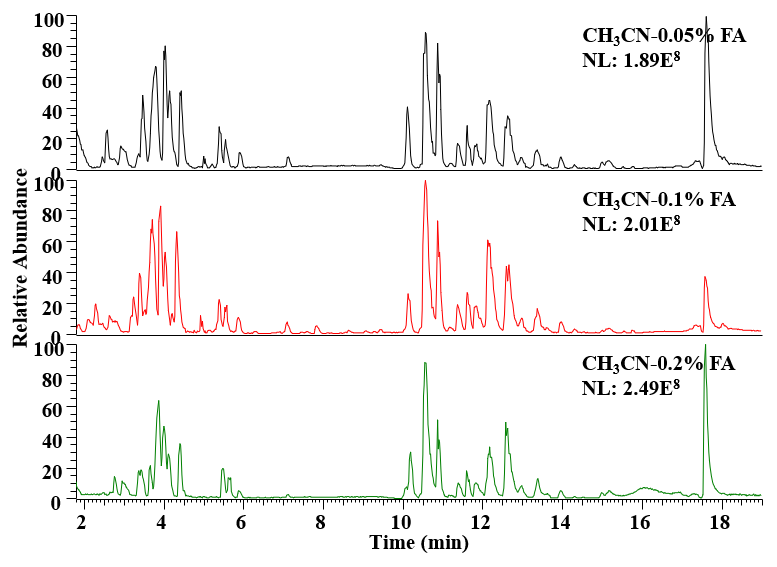


**Fig. 2S.10** Total ion chromatogram of separation of components from SR by different concentrations of formic acid (1.8-19 min)


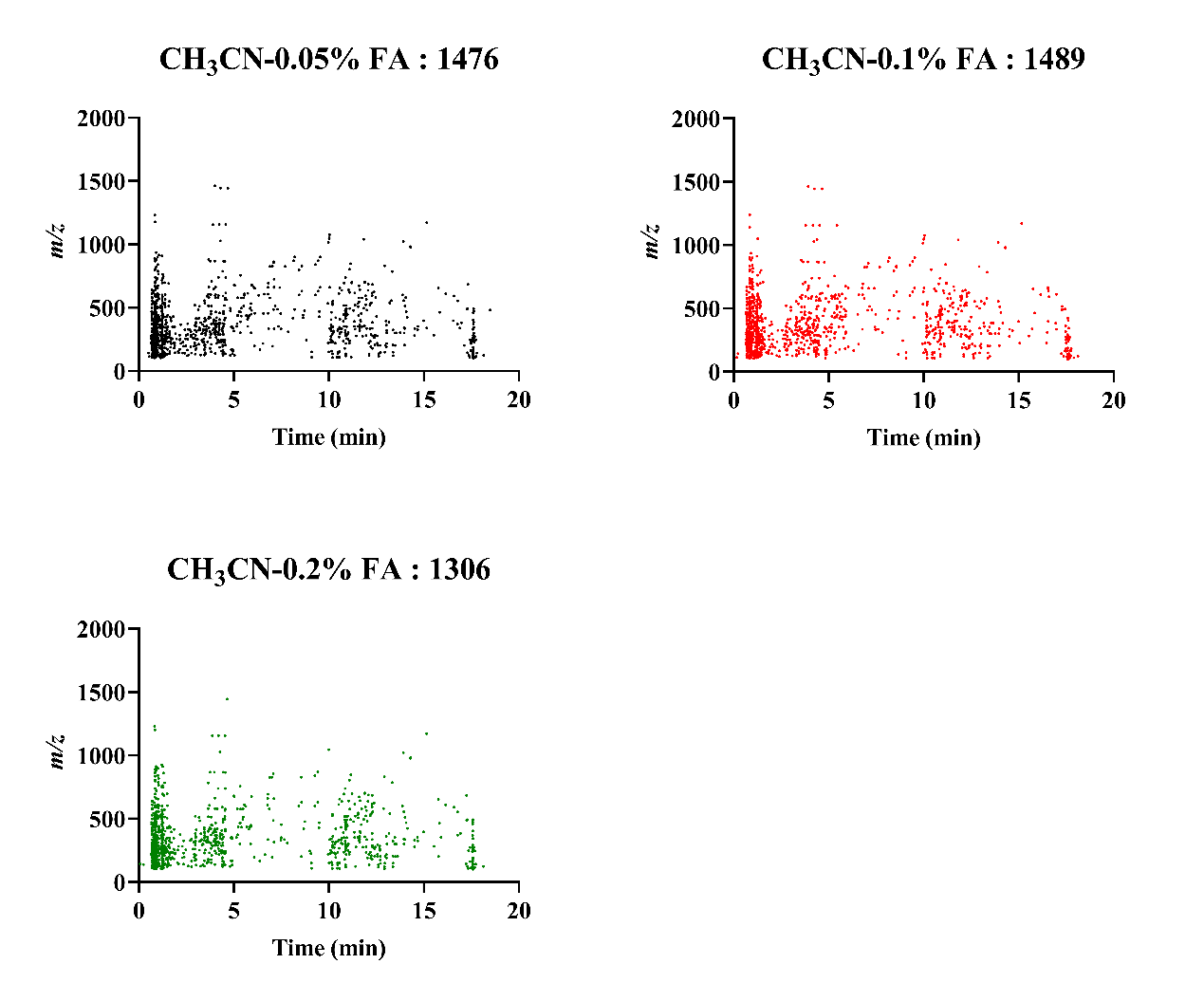


**Fig. 2S.11** Comparison of the number of ion chromatographic peaks with different formic acid concentrations in the aqueous phase (0-19 min)


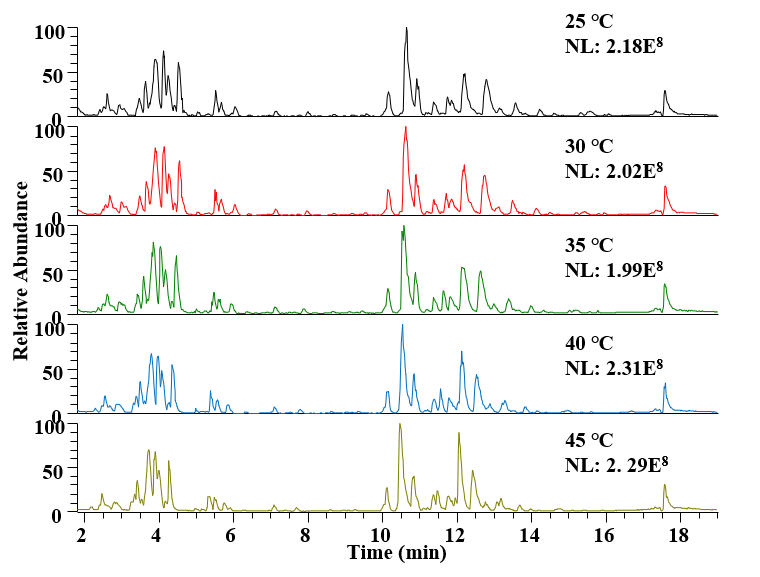


**Fig. 2S.12** Total ion chromatogram of separation of components from SR by different column temperatures (1.8-19 min)


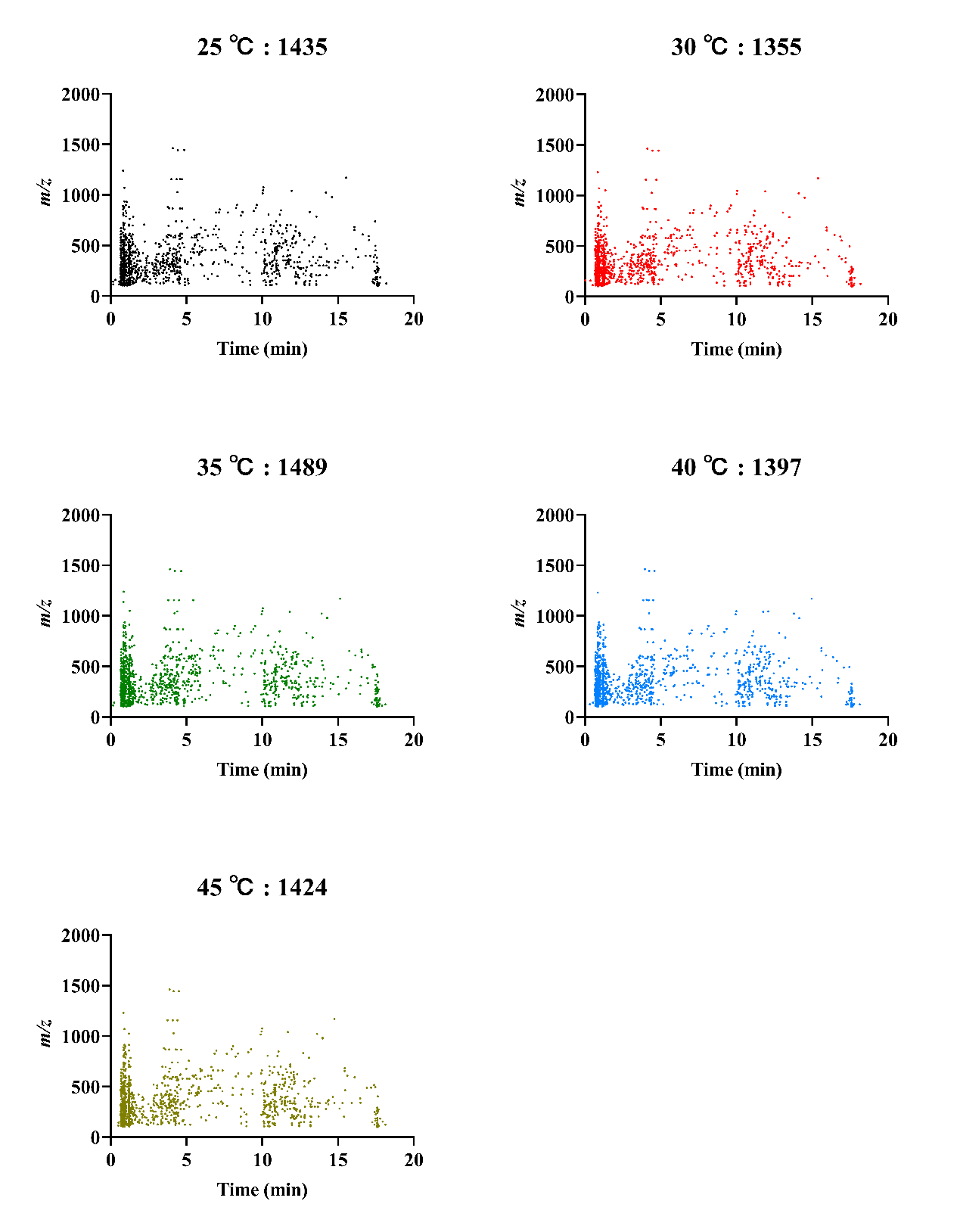


**Fig. 2S.13** Comparison of ion chromatographic peaks at different column temperatures (0-19 min)


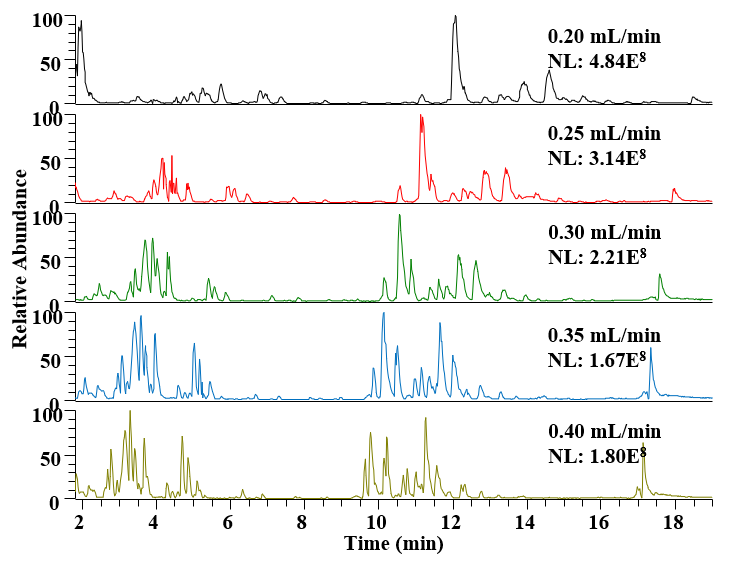


**Fig. 2S.14** Total ion chromatogram of separation of components from SR by different flow rates (1.8-19 min)


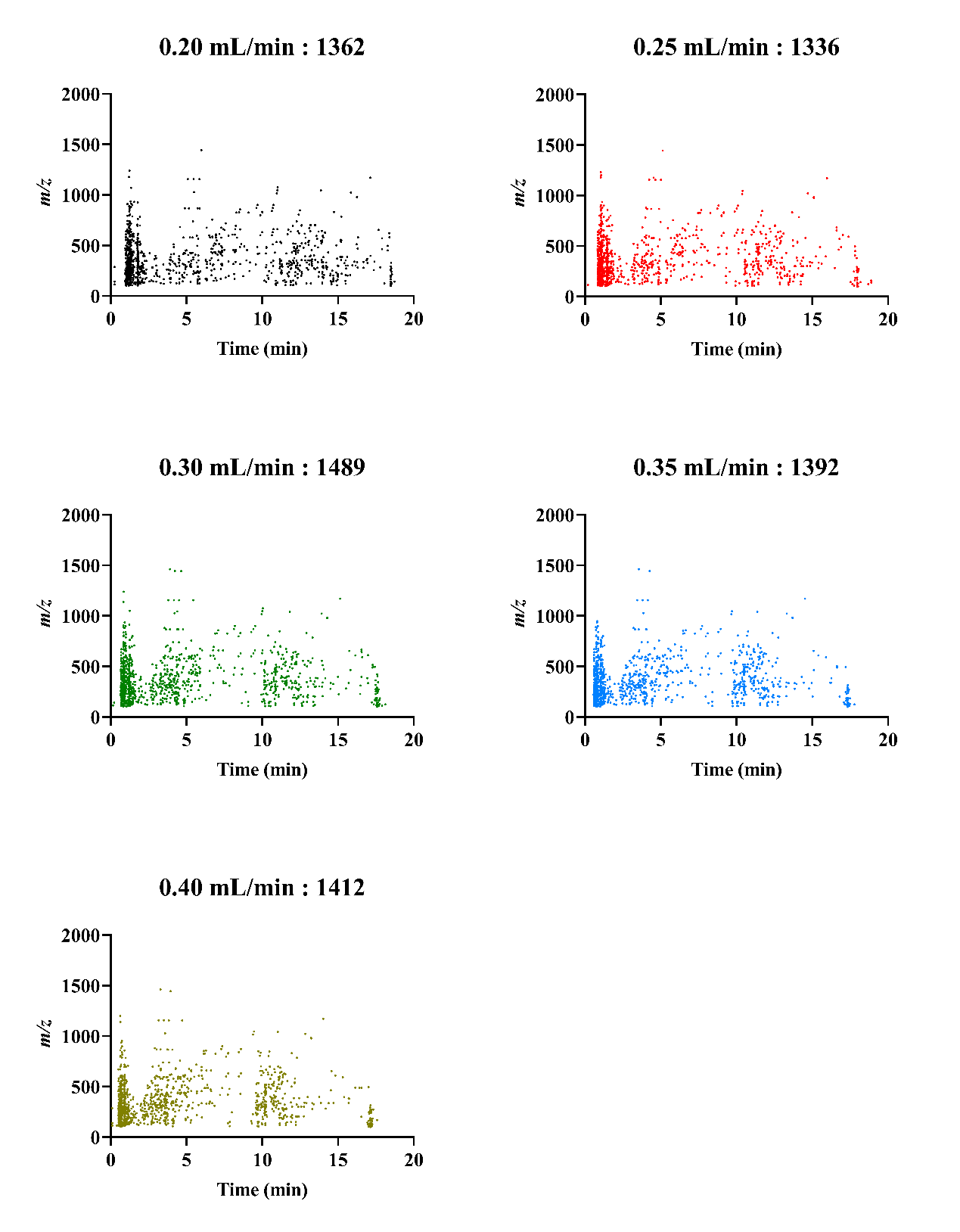


**Fig. 2S.15** Comparison of ion chromatographic peaks at different flow rates (0-19 min)


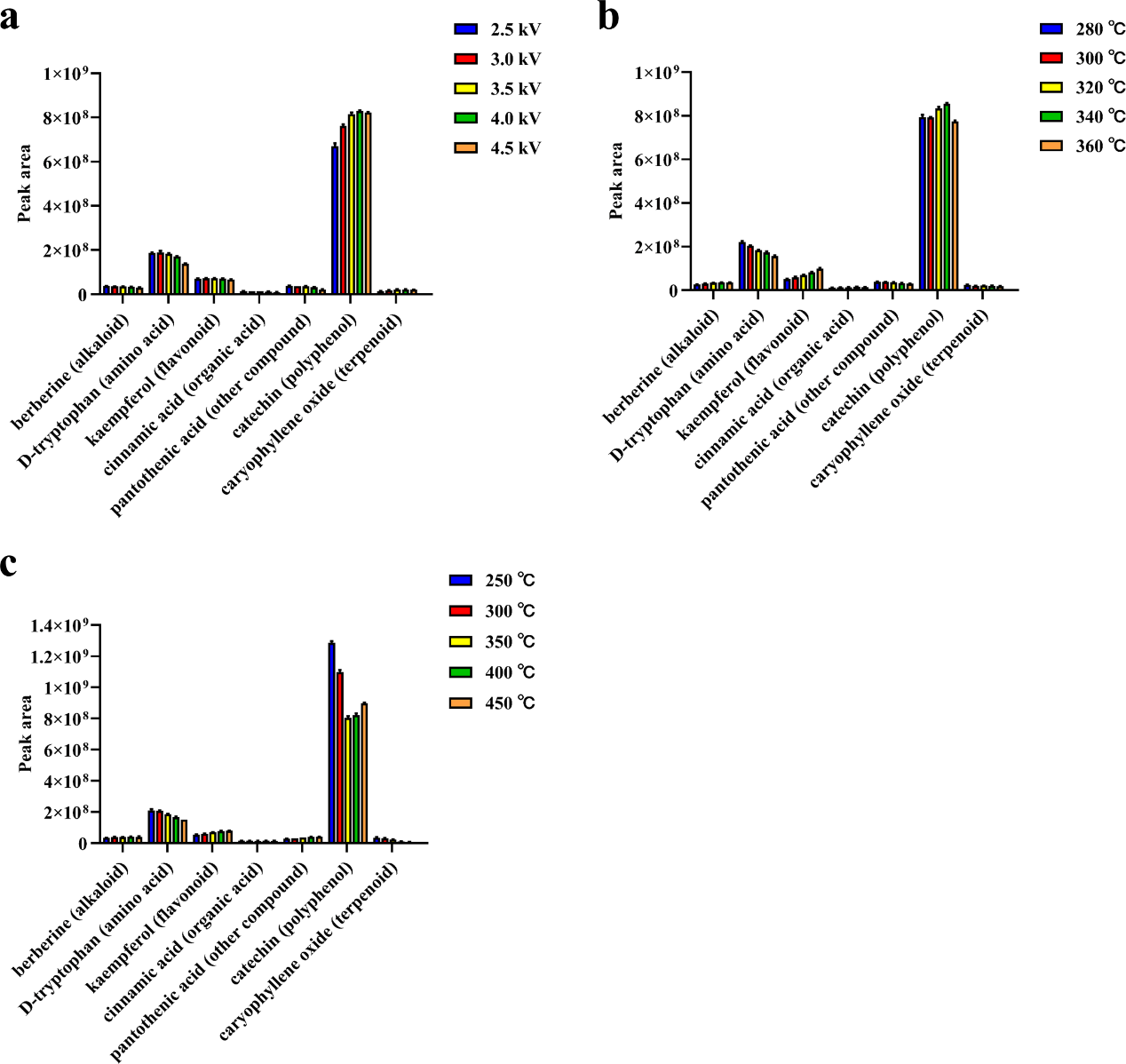


**Fig. 2S.16** Optimization of **a** spray voltage, **b** capillary temperature and **c** aux gas heater temperature in the positive mode of UPLC-MS/MS


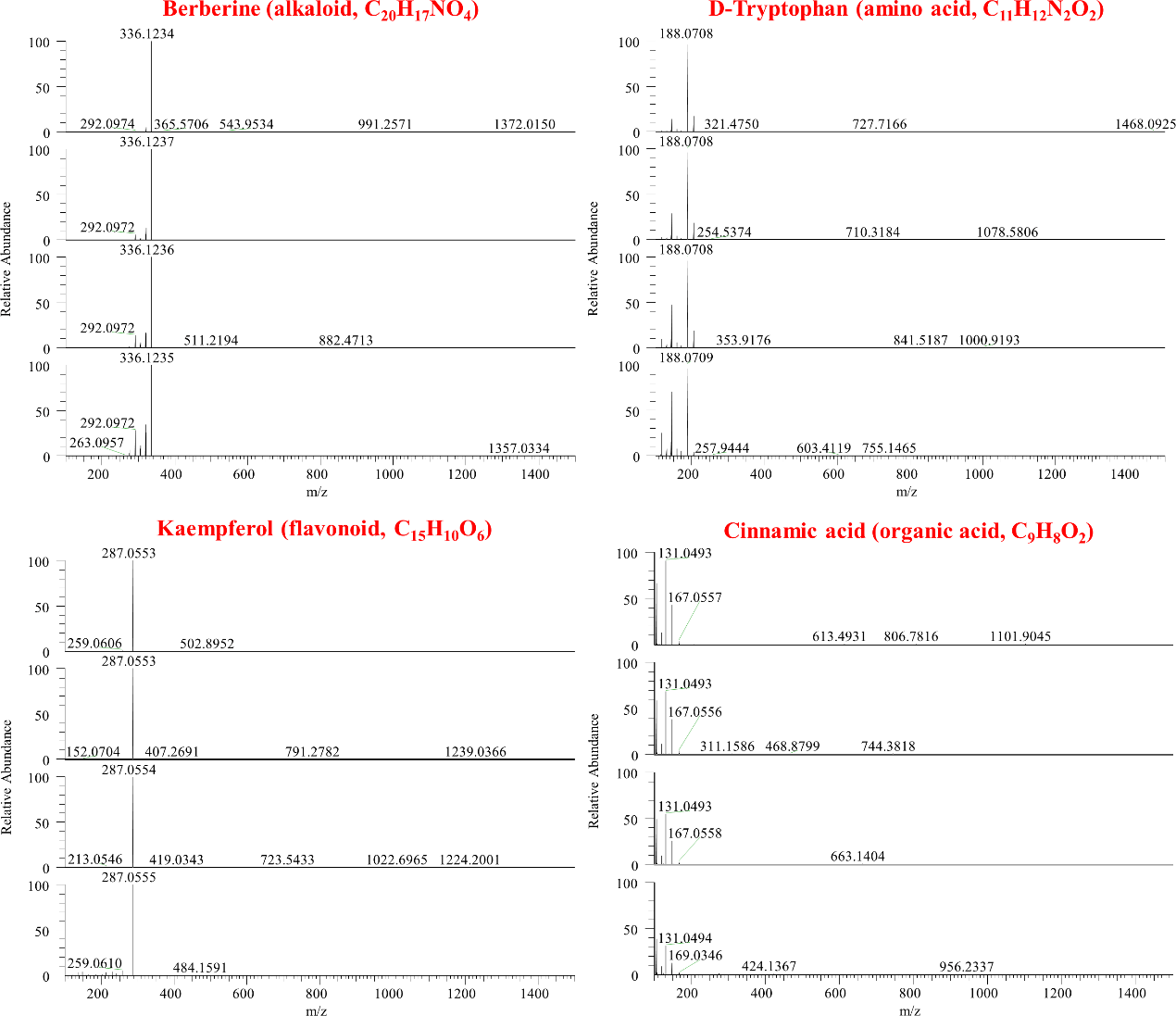


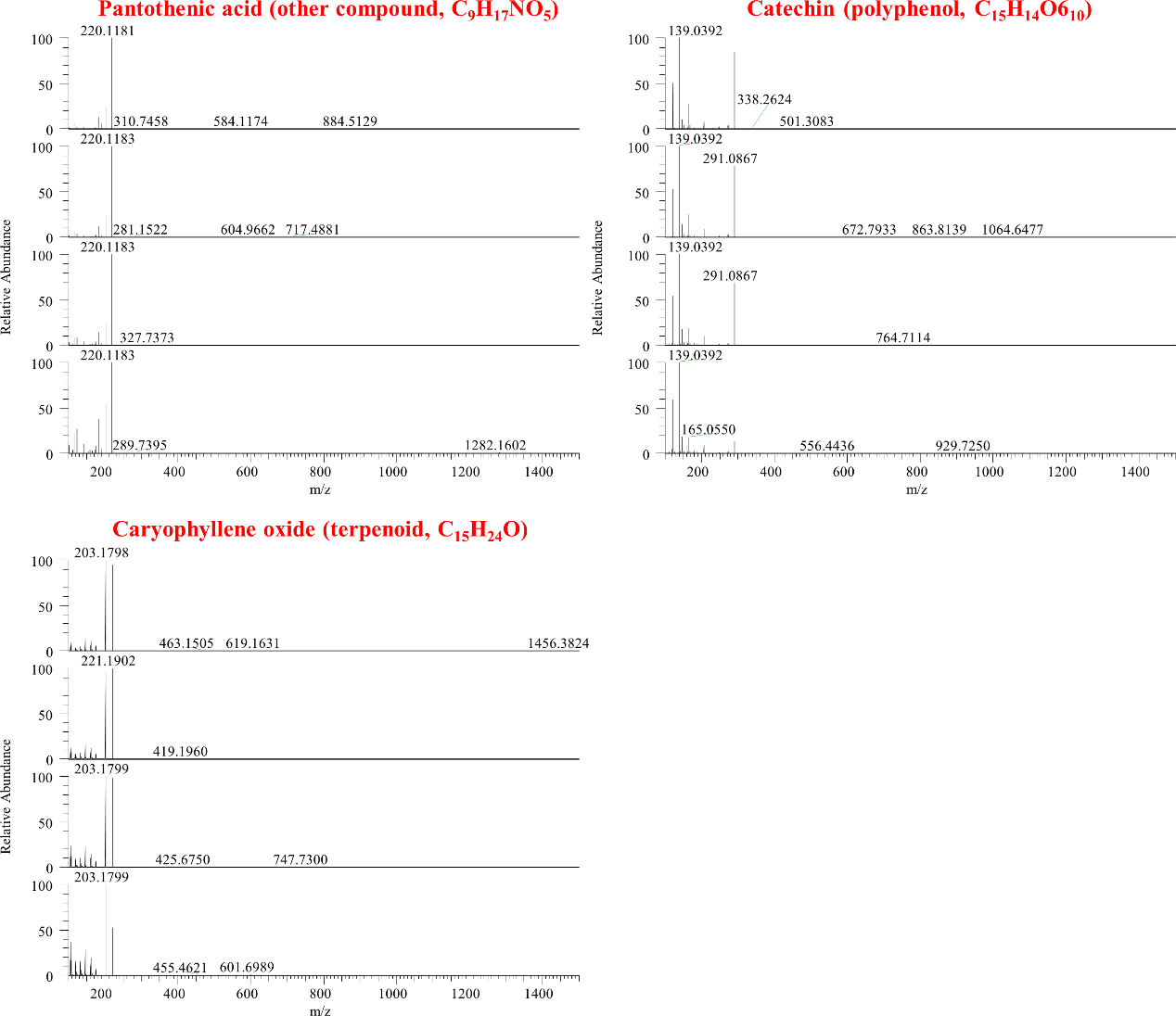


**Fig. 2S.17** Optimization of NCE in the positive mode of UPLC-MS/MS (set four NCEs from top to bottom as 10/20/30%; 10/20/40%; 10/30/50%; 20/40/60%)

3S. Condition optimization for HS-GC-MS/MS analysis

To analyze the compounds in SR more comprehensively and provide a basis for more accurate classification, a QC sample was used to optimize the conditions of HS-GC-MS/MS analysis.

The volatile substances in SR samples were heated in the HS heating chamber to volatilize. The heating temperature and time were optimized to ensure maximum detection and identification, as shown in Figs. 3S.1-3S.2 (see SI). The samples were heated at 100 ℃ for 20 min with the highest response and the largest number of peaks.


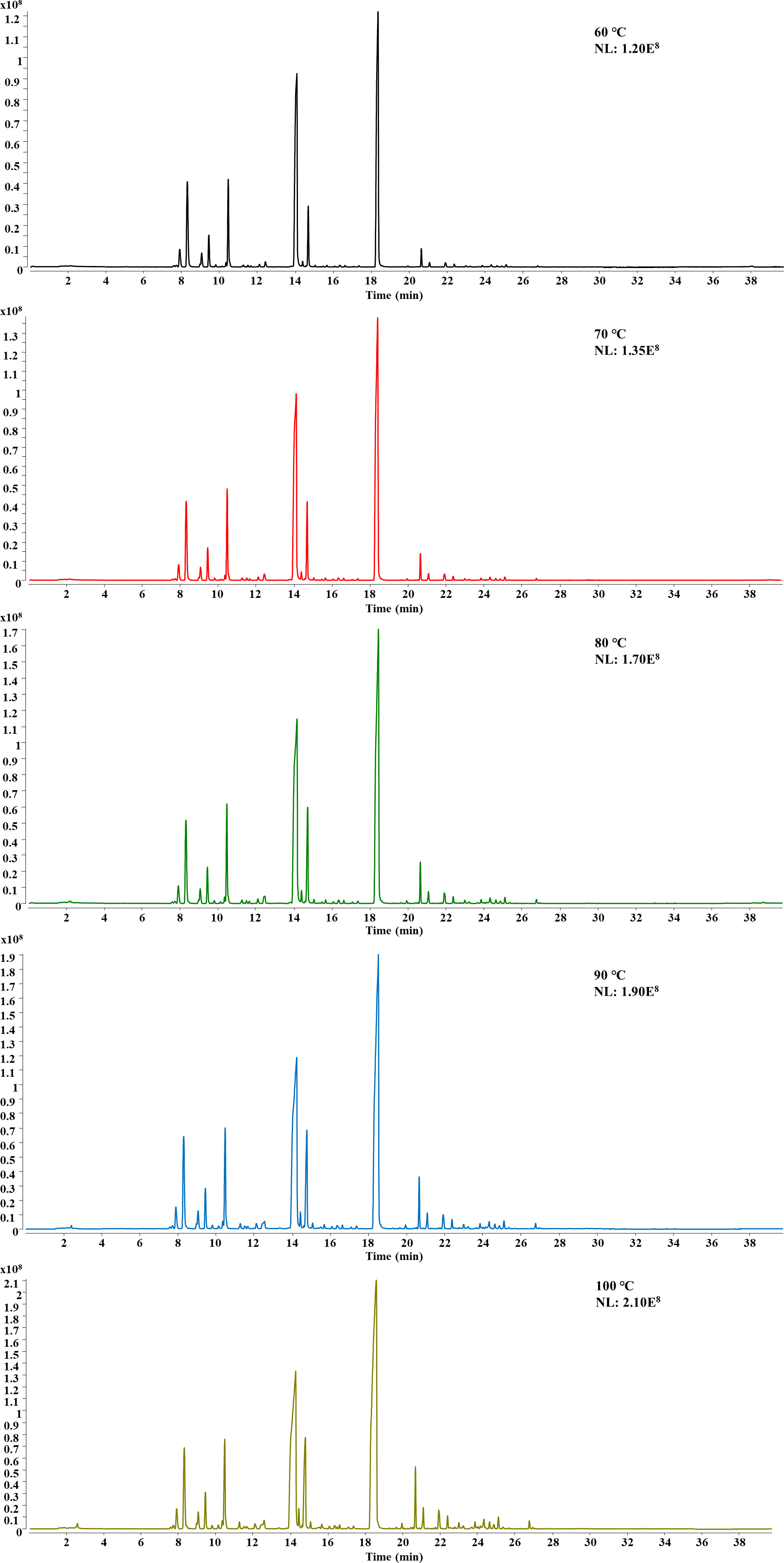


**Fig. 3S.1** Total ion chromatogram of separation of components from SR by different heating temperatures by HS-GC-MS/MS


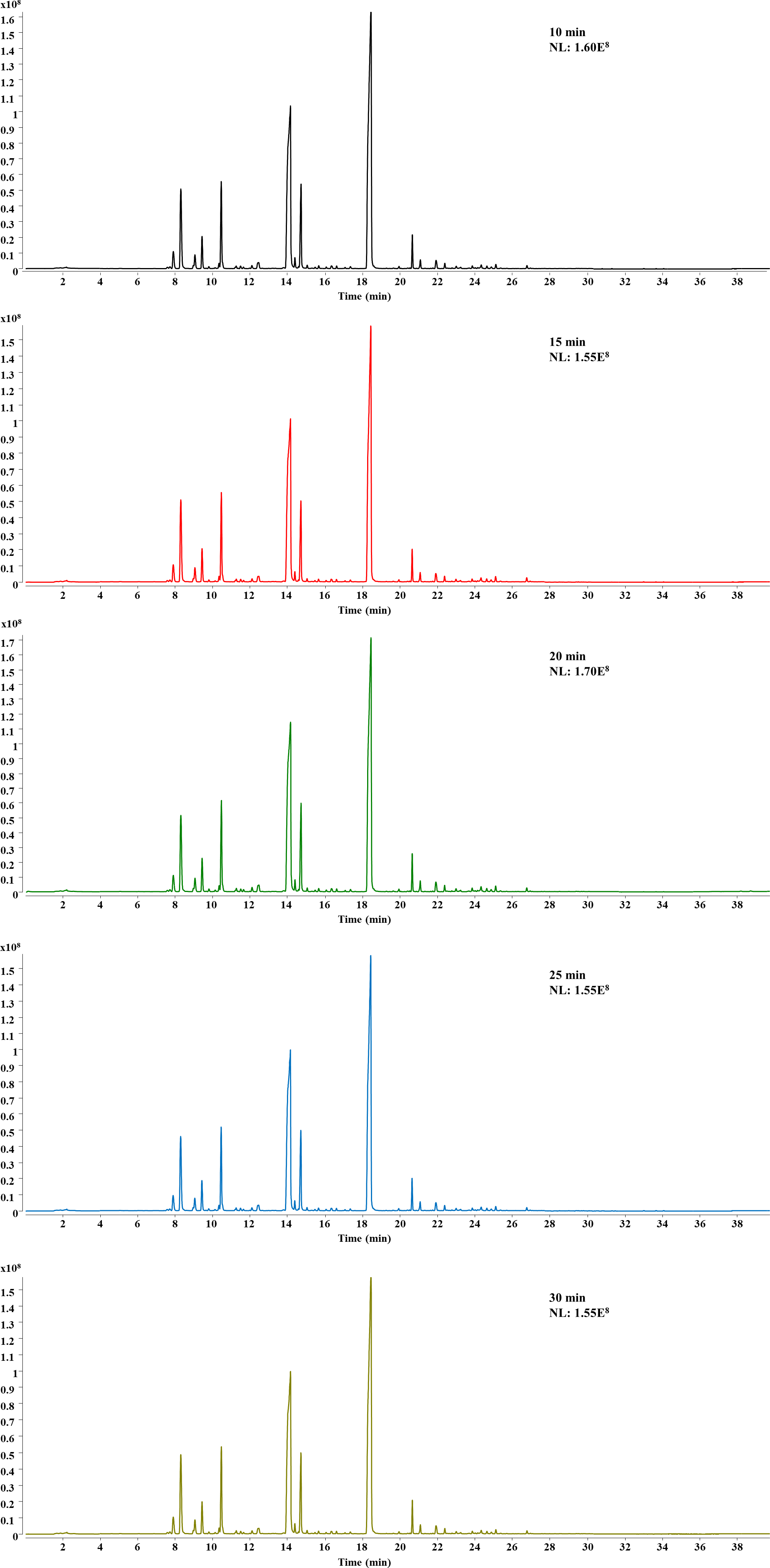


**Fig. 3S.2** Total ion chromatogram of separation of components from SR by different heating times by HS-GC-MS/MS

4S. Condition optimization for NMR analysis

For ^1^H NMR analysis, Bruker sequence, including zgpr, zg30, and noesygppr1d were optimized by using a QC sample to suppress the water signal, and the number of scans (NS) was also optimized to obtain spectra with better response, as shown in Figs. 4S.1-4S.2. The spectrum detected by the noesygppr1d sequence and 64 scans had the best effect on suppressing the water signal with a good response in a short time.


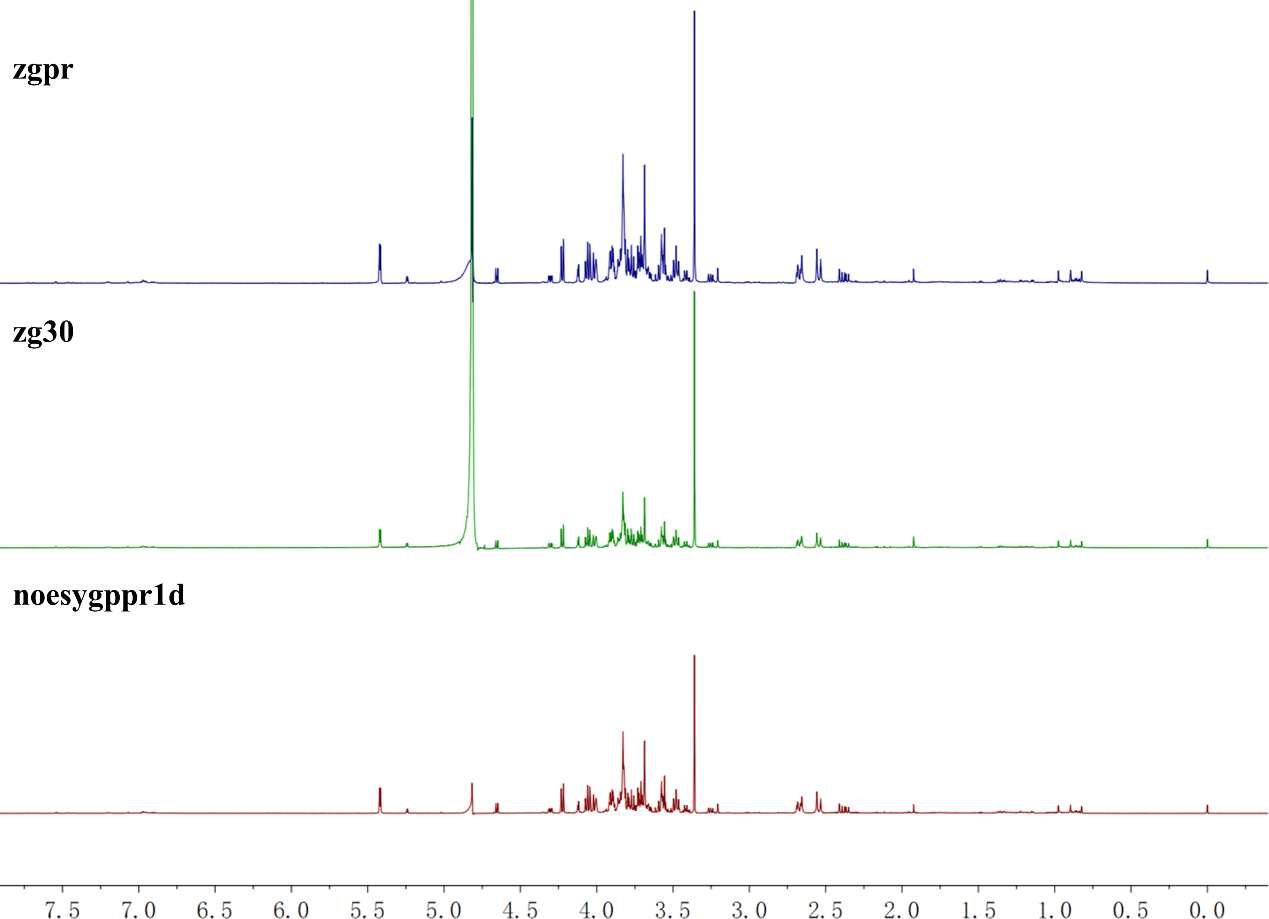


**Fig. 4S.1** Optimization of Bruker sequence of NMR


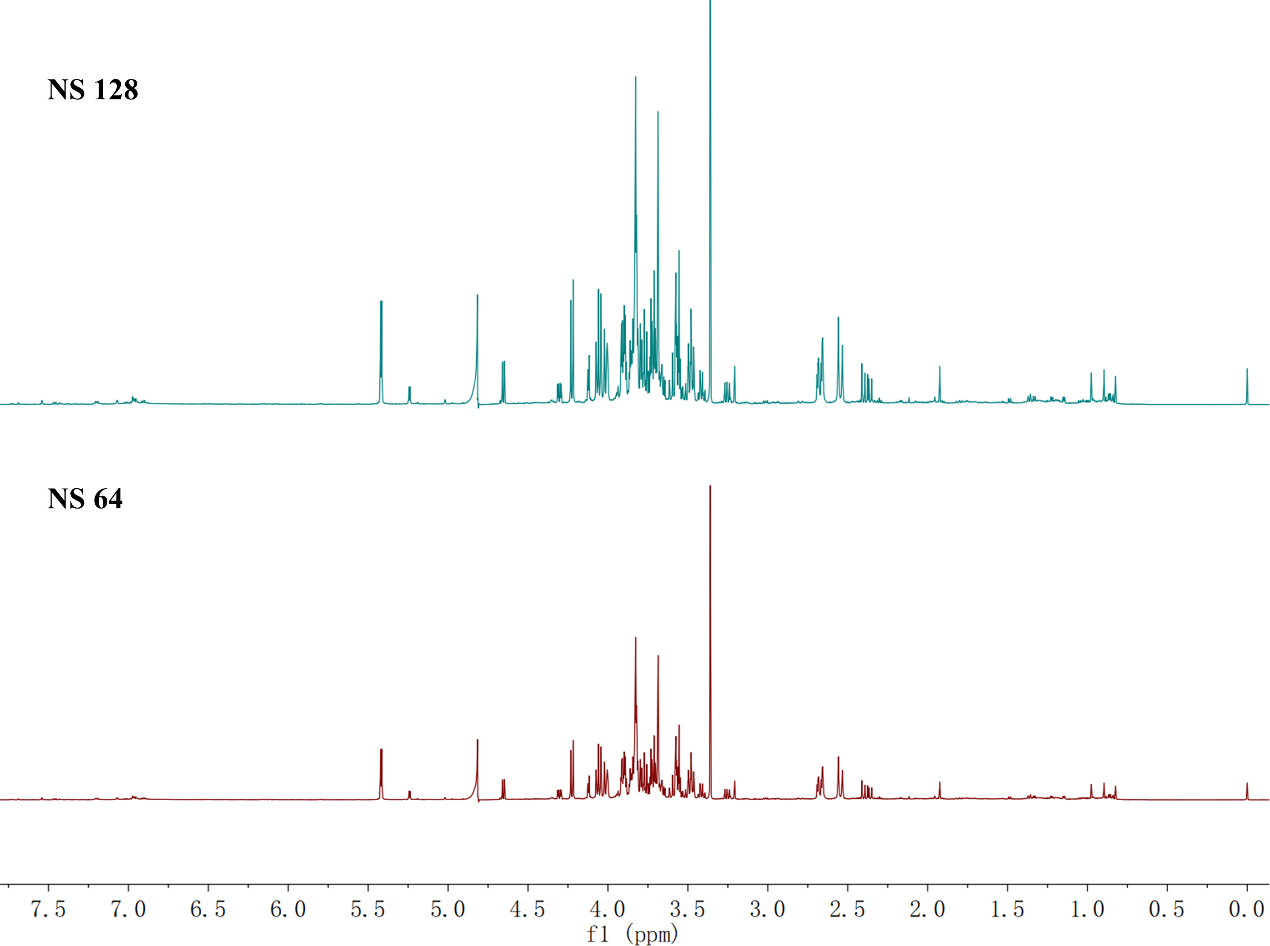


**Fig. 4S.2** Optimization of the number of scans of NMR

5S. Optimization for pretreatment method of MIR spectra

The Python package mentioned in part “2.6.4” of the manuscript was used to optimize the pre-processing methods of MIR spectra, including mean centralization (CT), first-order difference (D1), second-order difference (D2), moving average filtering (MA), max min normalization (MMS), multivariate scattering correction (MSC), SG, standard normal variate transform (SNV), SS, VN, and discrete wavelet transform (wave).

The spectrum of original MIR data was shown in Fig. 5S.1, the preprocessed MIR spectra were shown Fig. 5S.2, which were pretreated by CT, D1, D2, MA, MMS, MSC, SG, SNV, SS, VN, wave, respectively, and successively by SG and D2 (SG-D2), SG and SNV (SG-SNV), SG and VN (SG-VN), SNV and D2 (SNV-D2), SNV and SG (SNV-SG). According to the results, SG and VN pretreatment were finally performed on the MIR spectra in this study, to reduce noise and correct spectral errors caused by particle scattering.


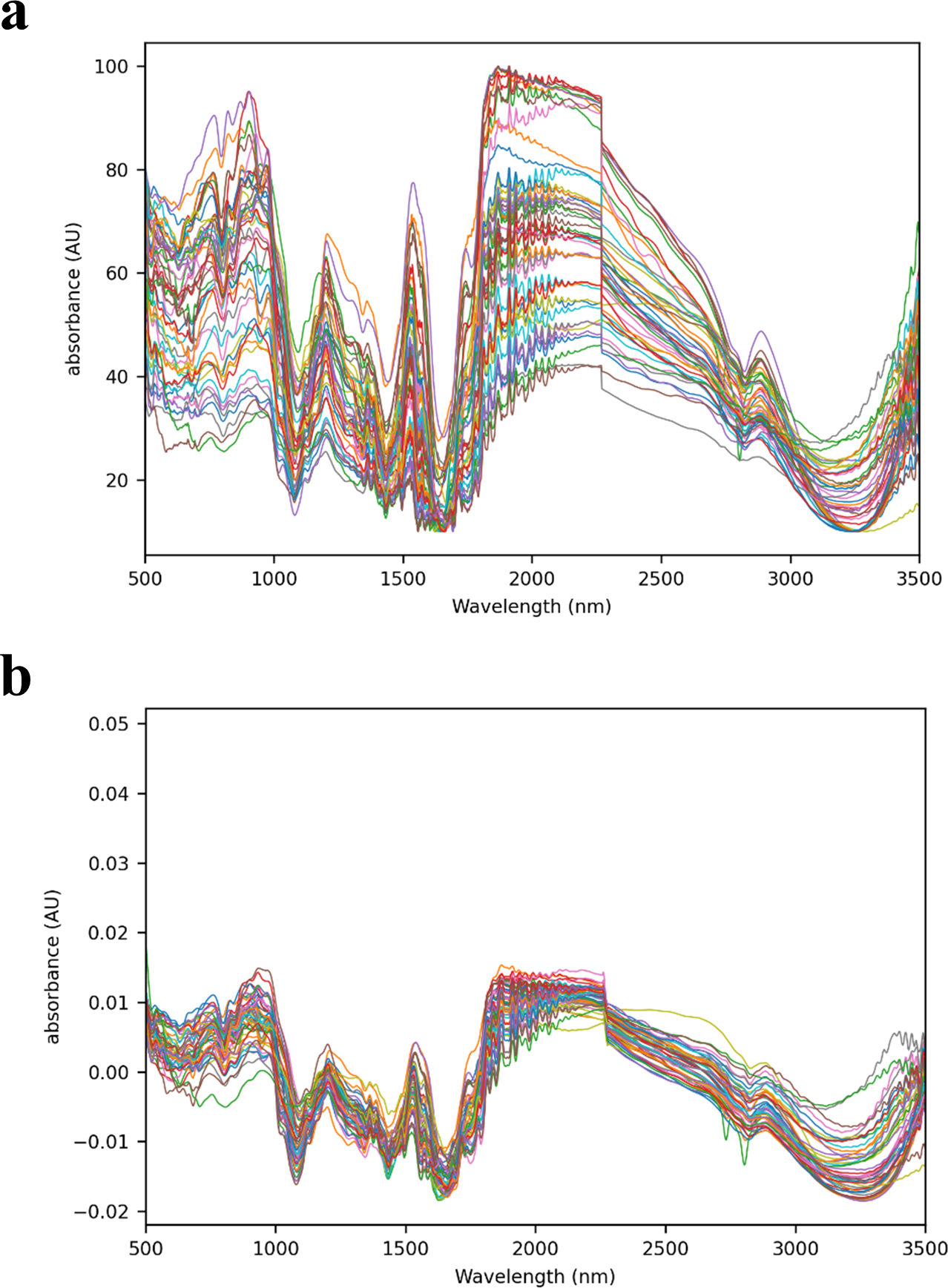


**Fig. 5S.1** Overlayed spectrum of raw MIR data


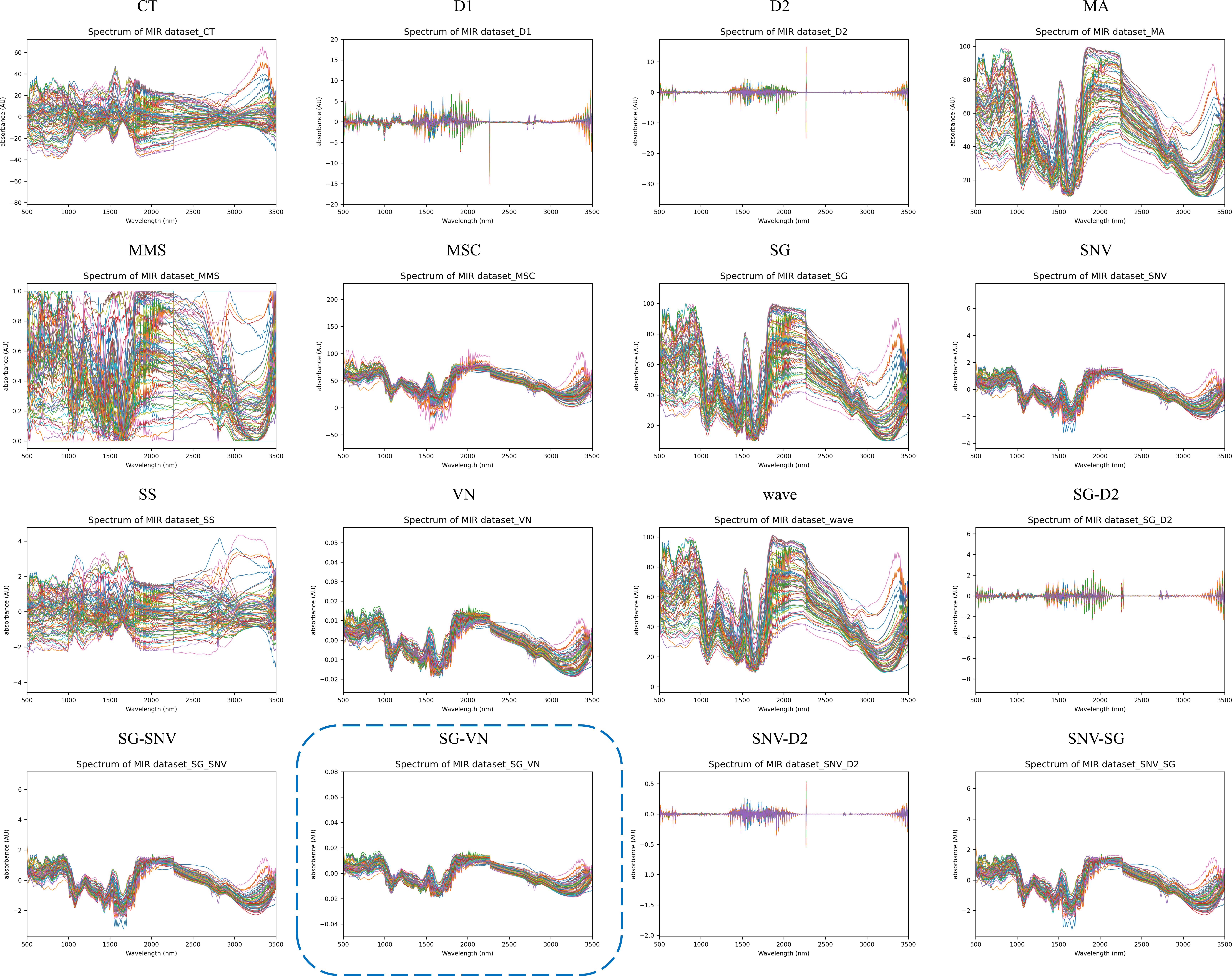


**Fig. 5S.2** Overlayed spectra of preprocessed MIR data

6S. Key parameters optimization for algorithms including PCA, PLS-DA, SVM, *k*NN, NN, DT, and RF

To obtain good model performance, the four datasets detected by UHPLC-Q-Orbitrap MS, HS-GC-MS/MS, NMR, and MIR were directly concatenated to build models for optimizing the key parameters of SVM, *k*NN, and NN algorithms by comparing accuracy train, accuracy test, precision score, f1 score, and recall score. As shown in Tables 6S.1-6S.3, SVM with kernel function of RBF, *k*NN with a *k* value of 3, and NN with 3 hidden layers (neuron numbers: 500, 200, and 10 respectively), showed good performance.

For MLDF, feature selection was applied to each dataset separately by PCA, PLS-DA, DT, and RF before the selected features were combined. The criterions of PCA and PLS-DA models were cumulative explained variance ratio and the criterions of RF and DT models were Gini values (i.e. the important variable with a high contribution value to the model classification effect).

In PCA, the number of PCs selected was determined according to the cumulative explained variance ratio. As shown in Fig. 6S.1, differences in the cumulative explained variance ratio had little effect on the classification results by the PCA model (PCA-PCA). To minimize information loss, a total of almost 95% variance ratio selected was respectively explained by the first thirty-five PCs obtained from UHPLC-Q-Orbitrap MS, the first eighteen PCs from HS-GC-MS/MS, the first ten PCs from NMR, and the first six PCs from MIR.

In PLS-DA, the number of LVs selected was also determined according to the cumulative explained variance ratio. As shown in Fig. 6S.2, because the two groups of data in the PLS-DA model (PLSDA-PLSDA) were well classified, a total of almost 95% variance ratio was selected, respectively explained by the first forty-five LVs obtained from UHPLC-Q-Orbitrap MS, the first twenty-three LVs from HS-GC-MS/MS, the first sixteen LVs from NMR, and the first six LVs from MIR, to get more information.

In DT, the number of variables with high importance was selected according to the classification effect (Table 6S.4) on the model established by DT (DT-DT). Then the first 40 important variables were selected for combination.

In RF, the number of important variables was selected according to the classification effect (Table 6S.5) on the model built by RF (RF-RF), and the first 40 or 120 both offered good results.

For the HLDF model (RF-RF-RF), data from four different sources were separately analyzed by RF, the first 120 features were respectively selected to construct RF models, and then the first 40 important variables were respectively selected for further data processing (according to the results shown in Table 6S.6).

The results of the algorithms modeled by their optimal parameters are shown in Fig. 6S.3. These key parameters were used for further analysis.

**Table 6S.1** SVM results with different kernel functions

| Kernel functions | Accuracy-train | Accuracy-test | Precision-score | f1-score | Recall-score |
| --- | --- | --- | --- | --- | --- |
| linear | 1.0000 | 0.8571 | 0.8947 | 0.9189 | 0.9444 |
| poly | 1.0000 | 0.8571 | 0.8947 | 0.9189 | 0.9444 |
| RBF | 1.0000 | 0.8571 | 0.8571 | 0.9231 | 1.0000 |
| sigmoid | 0.8776 | 0.8571 | 0.8571 | 0.9231 | 1.0000 |

**Table 6S.2** *k*NN results with different *k* values

| *k* values | Accuracy-train | Accuracy-test | Precision-score | f1-score | Recall-score |
| --- | --- | --- | --- | --- | --- |
| 1 | 1.0000 | 0.6190 | 0.8571 | 0.7500 | 0.6667 |
| 2 | 0.8776 | 0.8571 | 0.9412 | 0.9143 | 0.8889 |
| 3 | 0.8980 | 0.9524 | 0.9474 | 0.9730 | 1.0000 |
| 4 | 0.8776 | 0.6667 | 0.8667 | 0.7879 | 0.7222 |
| 5 | 0.8980 | 0.9048 | 0.9000 | 0.9474 | 1.0000 |
| 6 | 0.9184 | 0.7619 | 0.8824 | 0.8571 | 0.8333 |
| 7 | 0.8776 | 0.8571 | 0.8571 | 0.9231 | 1.0000 |
| 8 | 0.8776 | 0.8571 | 0.8571 | 0.9231 | 1.0000 |
| 9 | 0.8776 | 0.8571 | 0.8571 | 0.9231 | 1.0000 |
| 10 | 0.8776 | 0.8571 | 0.8571 | 0.9231 | 1.0000 |

**Table 6S.3** NN results with different hidden layers

| The number of hidden layers and hidden layer neurons | Accuracy-train | Accuracy-test | Precision-score | f1-score | Recall-score |
| --- | --- | --- | --- | --- | --- |
| 5, 2 | 0.8776 | 0.8571 | 0.8571 | 0.9231 | 1.0000 |
| 50, 20 | 1.0000 | 0.8571 | 0.8571 | 0.9231 | 1.0000 |
| 500, 200 | 1.0000 | 0.8095 | 0.9375 | 0.8824 | 0.8333 |
| 500, 200, 10 | 1.0000 | 0.8571 | 0.8571 | 0.9231 | 1.0000 |
| 500, 200, 10, 5 | 1.0000 | 0.8571 | 0.9412 | 0.9143 | 0.8889 |
| 500, 200, 10, 5, 1 | 0.8776 | 0.8571 | 0.8571 | 0.9231 | 1.0000 |


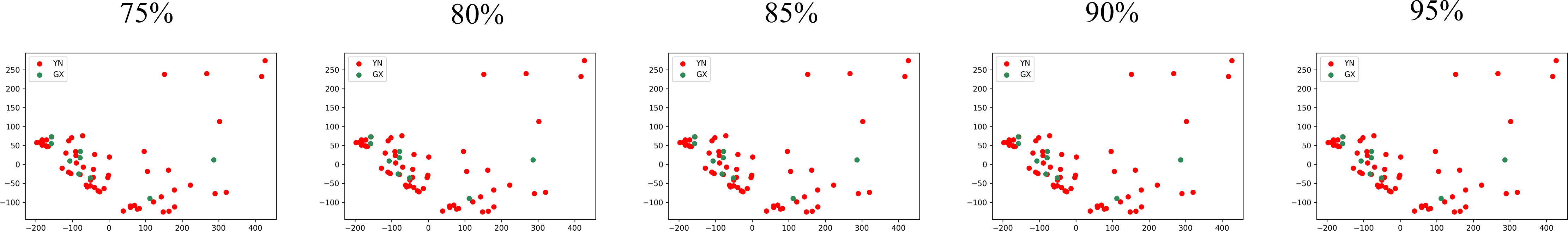


**Fig. 6S.1** Results of classification between YN and GX groups by PCA-PCA with different number of PCs selected according to the cumulative explained variance ratio


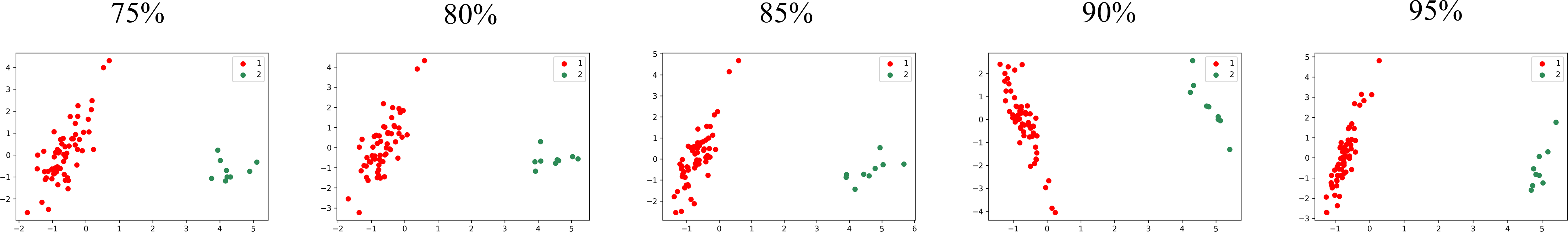


**Fig. 6S.2** Results of classification between YN (1) and GX (2) groups by PLSDA-PLSDA with different number of LVs selected according to the cumulative explained variance ratio

**Table 6S.4** DT-DT results with different number of selected important variables

| The number of selected important variables | Accuracy-train | Accuracy-test | Precision-score | f1-score | Recall-score |
| --- | --- | --- | --- | --- | --- |
| 10 | 1.0000 | 0.9524 | 1.0000 | 0.9714 | 0.9444 |
| 15 | 1.0000 | 0.9048 | 0.9000 | 0.9474 | 1.0000 |
| 20 | 1.0000 | 0.9048 | 0.9444 | 0.9444 | 0.9444 |
| 25 | 1.0000 | 0.8095 | 0.9375 | 0.8824 | 0.8333 |
| 30 | 1.0000 | 0.9048 | 0.9000 | 0.9474 | 1.0000 |
| 35 | 1.0000 | 0.9524 | 0.9474 | 0.9730 | 1.0000 |
| 40 | 1.0000 | 0.9524 | 0.9474 | 0.9730 | 1.0000 |
| 45 | 1.0000 | 0.9048 | 1.0000 | 0.9412 | 0.8889 |
| 50 | 1.0000 | 0.9524 | 0.9474 | 0.9730 | 1.0000 |
| 55 | 1.0000 | 0.8571 | 1.0000 | 0.9091 | 0.8333 |
| 60 | 1.0000 | 0.8095 | 0.9375 | 0.8824 | 0.8333 |
| 70 | 1.0000 | 0.8095 | 0.8889 | 0.8889 | 0.8889 |
| 80 | 1.0000 | 0.8571 | 0.8947 | 0.9189 | 0.9444 |
| 90 | 1.0000 | 0.9048 | 0.9444 | 0.9444 | 0.9444 |

**Table 6S.5** RF-RF results with different number of selected important variables

| The number of selected important variables | OOB | Accuracy-train | Accuracy-test | Precision-score | f1-score | Recall-score |
| --- | --- | --- | --- | --- | --- | --- |
| 10 | 0.8980 | 1.0000 | 0.8571 | 0.8947 | 0.9189 | 0.9444 |
| 15 | 0.9184 | 1.0000 | 0.9048 | 0.9000 | 0.9474 | 1.0000 |
| 20 | 0.8776 | 1.0000 | 0.9048 | 0.9444 | 0.9444 | 0.9444 |
| 25 | 0.8980 | 1.0000 | 0.9048 | 0.9000 | 0.9474 | 1.0000 |
| 30 | 0.9388 | 1.0000 | 0.9048 | 0.9000 | 0.9474 | 1.0000 |
| 35 | 0.9796 | 1.0000 | 0.8571 | 0.8571 | 0.9231 | 1.0000 |
| 40 | 0.9388 | 1.0000 | 0.9048 | 0.9000 | 0.9474 | 1.0000 |
| 45 | 0.9184 | 1.0000 | 0.9048 | 0.9000 | 0.9474 | 1.0000 |
| 50 | 0.8980 | 1.0000 | 0.9048 | 0.9000 | 0.9474 | 1.0000 |
| 55 | 0.8776 | 1.0000 | 0.9524 | 0.9474 | 0.9730 | 1.0000 |
| 60 | 0.9388 | 1.0000 | 0.7619 | 0.8421 | 0.8649 | 0.8889 |
| 70 | 0.9388 | 1.0000 | 0.8571 | 0.8947 | 0.9189 | 0.9444 |
| 80 | 0.9184 | 1.0000 | 0.9048 | 0.9000 | 0.9474 | 1.0000 |
| 90 | 0.8776 | 1.0000 | 0.9524 | 0.9474 | 0.9730 | 1.0000 |
| 100 | 0.8776 | 1.0000 | 0.8095 | 0.8500 | 0.8947 | 0.9444 |
| 110 | 0.8776 | 1.0000 | 0.9524 | 0.9474 | 0.9730 | 1.0000 |
| 120 | 0.8980 | 1.0000 | 0.9524 | 0.9474 | 0.9730 | 1.0000 |
| 130 | 0.8776 | 1.0000 | 0.9048 | 0.9000 | 0.9474 | 1.0000 |
| 140 | 0.8571 | 1.0000 | 0.9048 | 0.9000 | 0.9474 | 1.0000 |
| 150 | 0.8980 | 1.0000 | 0.9048 | 0.9000 | 0.9474 | 1.0000 |

**Table 6S.6** RF-RF-RF results with different number of selected important variables

| The number of selected important variables | OOB | Accuracy-train | Accuracy-test | Precision-score | f1-score | Recall-score |
| --- | --- | --- | --- | --- | --- | --- |
| 120-10 | 0.8980 | 1.0000 | 0.8095 | 0.8500 | 0.8947 | 0.9444 |
| 120-20 | 0.8980 | 1.0000 | 0.8095 | 0.8889 | 0.8889 | 0.8889 |
| 120-30 | 0.8980 | 1.0000 | 0.8095 | 0.8889 | 0.8889 | 0.8889 |
| 120-40 | 0.8776 | 1.0000 | 0.8571 | 0.8571 | 0.9231 | 1.0000 |
| 120-50 | 0.8776 | 1.0000 | 0.8571 | 0.8571 | 0.9231 | 1.0000 |
| 120-60 | 0.8367 | 1.0000 | 0.8571 | 0.8571 | 0.9231 | 1.0000 |
| 120-70 | 0.8776 | 1.0000 | 0.6667 | 0.8235 | 0.8000 | 0.7778 |


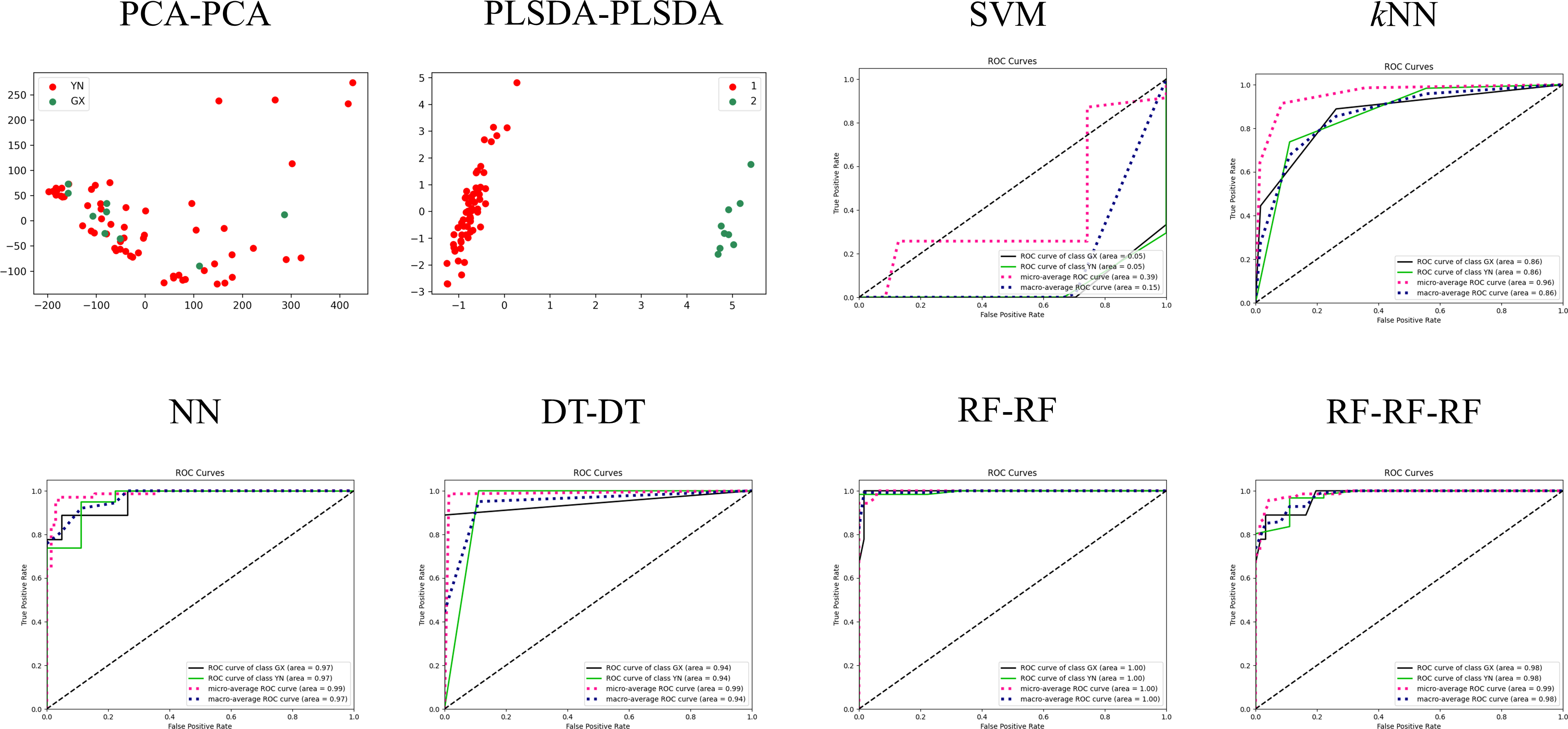


**Fig. 6S.3** The classification results between YN (1) and GX (2) groups of the algorithms (including PCA-PCA, PLSDA-PLSDA, SVM, *k*NN, NN, DT-DT, RF-RF, RF-RF-RF) modeled by their optimal parameters

7S. Comprehensive characterization of multi-components in SR

To comprehensively characterize the compounds in SR, the systematic detection methods was established by UHPLC-Q-Orbitrap MS, to detect non-volatile compounds, HS-GC-MS/MS, to analyze volatile compounds, NMR, to supplement the results of UHPLC-Q-Orbitrap MS, and MIR, to make a fast detection. A total of 286 compounds and functional group information were detected, as shown in Tables 7S.1-7S.3 and Fig. 7S.1.

**Table 7S.1** Tentative characterization of chemical constituents of SR by UHPLC-Q-Orbitrap MS in positive and negative ion mode

| Comp. | *t_R_* (min) | *m/z* | Ion mode | Formula | RDB | Mass error (ppm) | MS/MS  (*m/z*) | Identification |
| --- | --- | --- | --- | --- | --- | --- | --- | --- |
| 1 | 0.74 | 245.0431 | negative | C_6_H_15_O_8_P | 0.0 | 1.855 | 152.9953 | Glycerophosphoglycerol |
| 2 | 0.75 | 110.0713 | positive | C_5_H_7_N_3_ | 2.0 | 2.804 | 81.0453, 67.0280, 59.0609 | 2-amino-4-methylpyrimidine |
| 3 | 0.78 | 154.0625 | negative | C_6_H_9_N_3_O_2_ | 2.5 | 1.067 | 110.0724, 83.0608 | *L*-Histidine* |
| 4 | 0.82 | 341.1089 | negative | C_12_H_22_O_11_ | 2.0 | 0.324 | 179.0564, 161.0458, 149.0457, 143.0356, 131.0355, 101.0246 | Trehalose |
| 5 | 0.83 | 104.0706 | positive | C_4_H_9_NO_2_ | 1.0 | 2.926 | 60.0815, 58.0658 | *γ*-Aminobutyric acid* |
| 6 | 0.84 | 151.0612 | negative | C_5_H_12_O_5_ | 0.0 | 2.754 | 133.0501, 121.0501, 71.0124, 59.0133 | Xylitol* |
| 7 | 0.85 | 248.1133 | positive | C_10_H_17_NO_6_ | 2.5 | 1.758 | 230.1027, 212.0927, 126.0551 | Linamarin |
| 8 | 0.86 | 175.1190 | positive | C_6_H_14_N_4_O_2_ | 2.0 | 2.161 | 116.0708, 112.0873, 70.0658 | Arginine* |
| 9 | 0.86 | 116.0706 | positive | C_5_H_9_NO_2_ | 2.0 | 1.126 | 71.0501, 53.0391 | D-Proline* |
| 10 | 0.87 | 193.0354 | negative | C_6_H_10_O_7_ | 2.0 | 3.617 | 149.0455, 93.0346, 71.0125 | *β*-D-Glucopyranuronic acid* |
| 11 | 0.87 | 131.0350 | negative | C_5_H_8_O_4_ | 2.0 | 1.981 | 113.0239, 87.0446, 59.0133 | Glutaric acid* |
| 12 | 0.87 | 195.0510 | negative | C_6_H_12_O_7_ | 1.0 | 1.314 | 177.0408, 147.0300, 105.0188, 101.0244 | Gluconic acid |
| 13 | 0.92 | 133.0145 | negative | C_4_H_6_O_5_ | 2.0 | 2.411 | 115.0038, 89.0239, 59.0124 | D-Malic acid |
| 14 | 0.93 | 147.0299 | negative | C_5_H_8_O_5_ | 2.0 | 2.912 | 129.0196, 103.0400, 101.0250 | D-2-hydroxyglutaric acid |
| 15 | 0.94 | 175.0248 | negative | C_6_H_8_O_6_ | 3.0 | 2.457 | 99.0082, 61.0290 | Vitamin C* |
| 16 | 1.02 | 191.0198 | negative | C_6_H_8_O_7_ | 3.5 | 1.130 | 173.0090, 129.0195, 111.0088 | Citric acid* |
| 17 | 1.02 | 173.0093 | negative | C_6_H_6_O_6_ | 4.5 | 1.142 | 154.9990, 129.0197, 111.0089 | Dehydroascorbic acid |
| 18 | 1.07 | 120.0655 | positive | C_4_H_9_NO_3_ | 1.0 | 1.764 | 102.0555, 56.0500 | *L*-Threonine* |
| 19 | 1.16 | 118.0864 | positive | C_5_H_11_NO_2_ | 0.5 | 1.227 | 101.0605, 72.0813, 56.0501 | *N*-Methyl-*α*-aminoisobutyric acid |
| 20 | 1.21 | 166.0863 | positive | C_9_H_11_NO_2_ | 5.0 | 2.269 | 120.0810, 79.0540 | Phenylalanine* |
| 21 | 1.21 | 123.0553 | positive | C_6_H_6_N_2_O | 5.0 | 3.666 | 105.0450, 79.0184 | Nicotinamide |
| 22 | 1.22 | 148.0604 | positive | C_5_H_9_NO_4_ | 2.0 | 2.057 | 102.0966, 71.0124 | Glutamic acid* |
| 23 | 1.23 | 133.0495 | positive | C_5_H_8_O_4_ | 2.0 | 2.738 | 59.0124 | Methylsuccinic acid |
| 24 | 1.23 | 133.0506 | negative | C_5_H_10_O_4_ | 1.0 | 2.518 | 71.0125, 115.0038 | 2,3-dihydroxy-3-methylbutanoic acid |
| 25 | 1.25 | 129.0194 | negative | C_5_H_6_O_4_ | 3.5 | 2.028 | 85.0290, 59.0124 | Citraconic acid |
| 26 | 1.26 | 123.0442 | positive | C_7_H_6_O_2_ | 4.5 | 1.170 | 106.0288, 105.0370 | Benzoic acid |
| 27 | 1.26 | 115.0401 | negative | C_5_H_8_O_3_ | 2.0 | 0.931 | 69.0340, 55.0184 | *α*-Ketoisovaleric acid |
| 28 | 1.26 | 130.0863 | positive | C_6_H_11_NO_2_ | 2.0 | 2.514 | 82.0663, 69.0603 | *L*-Pipecolic acid |
| 29 | 1.26 | 130.0501 | positive | C_5_H_7_NO_3_ | 2.5 | 1.771 | 84.0450, 56.0503 | *L*-Pyroglutamic acid |
| 30 | 1.36 | 182.0814 | positive | C_9_H_11_NO_3_ | 4.5 | 1.264 | 123.0443, 119.0494 | *L*-Tyrosine* |
| 31 | 1.36 | 181.0720 | positive | C_7_H_8_N_4_O_2_ | 4.0 | -2.094 | 136.0759, 123.0443 | Theophylline |
| 32 | 1.39 | 152.0706 | positive | C_8_H_9_NO_2_ | 5.0 | 3.442 | 109.0510, 65.0391 | Methyl 2-aminobenzoate |
| 33 | 1.40 | 115.0036 | negative | C_4_H_4_O_4_ | 4.5 | 2.825 | 71.0123, 53.0027 | Fumaric acid |
| 34 | 1.44 | 117.0193 | negative | C_4_H_6_O_4_ | 2.0 | 1.268 | 73.0290, 71.0124 | 4-hydroxy-2-oxobutanoic acid |
| 35 | 1.50 | 132.1022 | positive | C_6_H_13_NO_2_ | 0.5 | 2.231 | 86.0969, 69.0705 | Norleucine* |
| 36 | 1.55 | 161.0455 | negative | C_6_H_10_O_5_ | 2.0 | 1.709 | 71.0139, 59.0124 | 3-hydroxy-3-methylglutaric acid |
| 37 | 1.57 | 173.0455 | negative | C_7_H_10_O_5_ | 3.0 | 0.741 | 111.0089, 108.9636, 55.0180 | Shikimic acid* |
| 38 | 1.62 | 101.0244 | negative | C_4_H_6_O_3_ | 2.0 | 2.394 | 83.0133, 73.0124, 57.0340 | 2-ketobutyric acid |
| 39 | 1.71 | 127.0392 | positive | C_6_H_6_O_3_ | 3.5 | 1.806 | 109.0286, 71.0125 | Maltol |
| 40 | 1.71 | 127.0392 | positive | C_6_H_6_O_3_ | 3.5 | 1.806 | 110.0238, 109.0286 | 1,3,5-trihydroxybenzene |
| 41 | 1.81 | 154.0499 | positive | C_7_H_7_NO_3_ | 5.0 | 0.896 | 136.0396, 111.0443, 108.0447 | 3-amino-4-hydroxybenzoic acid* |
| 42 | 1.81 | 103.0401 | negative | C_4_H_8_O_3_ | 1.0 | 2.770 | 85.0295, 67.0189, 59.0139 | 3-hydroxybutyric acid* |
| 43 | 1.81 | 154.0499 | positive | C_7_H_7_NO_3_ | 2.5 | 2.970 | 136.0396, 108.0447 | 3-aminosalicylic acid |
| 44 | 1.91 | 144.0478 | positive | C_6_H_9_NOS | 3.0 | 0.738 | 126.0369, 114.0373 | 5-(2-hydroxyethyl)-4-methylthiazole |
| 45 | 2.00 | 143.0339 | positive | C_6_H_6_O_4_ | 4.0 | 1.617 | 81.0335, 79.0178 | Muconic acid |
| 46 | 2.03 | 205.0357 | negative | C_7_H_10_O_7_ | 3.5 | 0.930 | 187.0259, 145.0140, 143.0351, 101.0246 | 3-(carboxymethyl)-3-hydroxypentanedioic acid |
| 47 | 2.03 | 111.0087 | negative | C_5_H_4_O_3_ | 4.5 | 3.274 | 83.0139, 67.0189 | 3-furoic acid |
| 48 | 2.08 | 331.0671 | negative | C_13_H_16_O_10_ | 3.0 | 3.921 | 169.0148, 151.0037, 125.0246, 123.0088 | Glucogallin |
| 49 | 2.12 | 179.0826 | negative | C_9_H_12_N_2_O_2_ | 5.0 | 1.391 | 161.0463, 58.0293 | Pyridostigmine |
| 50 | 2.20 | 193.0976 | positive | C_10_H_12_N_2_O_2_ | 5.5 | 2.309 | 139.0026,108.0447 | Cotinine *N*-oxide |
| 51 | 2.41 | 149.0597 | positive | C_9_H_8_O_2_ | 2.0 | 0.882 | 131.0493, 105.0704, 103.0544 | Cinnamic acid* |
| 52 | 2.53 | 111.0452 | negative | C_6_H_8_O_2_ | 3.0 | 1.309 | 95.0493, 67.0544 | Sorbic acid |
| 53 | 2.54 | 142.0863 | positive | C_7_H_11_NO_2_ | 3.0 | 1.583 | 124.0760, 71.0861 | Hypoglycin a |
| 54 | 2.62 | 127.0392 | positive | C_6_H_6_O_3_ | 3.5 | 1.806 | 109.0286, 59.0124 | Pyrogallol |
| 55 | 2.72 | 220.1184 | positive | C_9_H_17_NO_5_ | 1.5 | 2.048 | 103.0757, 72.0450, 57.0705 | Pantothenic acid* |
| 56 | 2.74 | 218.1047 | negative | C_10_H_13_N_5_O | 4.0 | 0.632 | 133.0141, 71.0501 | Zeatin |
| 57 | 2.75 | 133.0495 | positive | C_5_H_8_O_4_ | 2.0 | 0.981 | 71.0491, 69.0335 | Ethylmalonic acid |
| 58 | 2.83 | 329.0882 | negative | C_14_H_18_O_9_ | 2.5 | 1.532 | 209.0456, 167.0351, 123.0453 | Vanilloyl glucose |
| 59 | 3.06 | 139.0390 | positive | C_7_H_6_O_3_ | 5.0 | 1.914 | 121.0290, 91.0184, 79.0185 | 4-hydroxybenzoic acid* |
| 60 | 3.14 | 299.0776 | negative | C_13_H_16_O_8_ | 4.5 | 3.869 | 239.0564, 179.0352, 143.0355, 137.0246, 101.0245 | 1-(4-hydroxybenzoyl) glucose |
| 61 | 3.16 | 153.0195 | negative | C_7_H_6_O_4_ | 5.5 | 2.266 | 109.0296, 108.0218 | Gentisic acid |
| 62 | 3.18 | 127.0754 | positive | C_7_H_10_O_2_ | 3.0 | 1.686 | 109.0649, 93.0340, 57.0340 | 3,5-dimethyl-1,2-cyclopentanedione |
| 63 | 3.22 | 109.0296 | negative | C_6_H_6_O_2_ | 4.0 | 1.989 | 83.0130, 79.0184 | Catechol |
| 64 | 3.31 | 298.0976 | positive | C_11_H_15_N_5_O_3_S | 1.5 | 2.561 | 163.0428, 145.0323, 136.0620 | 5'-methylthioadenosine |
| 65 | 3.32 | 225.0758 | positive | C_11_H_12_O_5_ | 1.0 | 3.905 | 179.0708, 139.0395, 95.0133 | Sinapic acid* |
| 66 | 3.32 | 169.0498 | positive | C_8_H_8_O_4_ | 4.5 | 1.566 | 125.0599, 81.0340 | Isovanillic acid |
| 67 | 3.34 | 229.1547 | positive | C_11_H_20_N_2_O_3_ | 3.0 | 3.067 | 183.1130, 116.0709 | Leucylproline |
| 68 | 3.36 | 125.0599 | positive | C_7_H_8_O_2_ | 3.5 | 1.551 | 65.0388, 79.0542, 107.0494 | 3-hydroxybenzyl alcohol* |
| 69 | 3.39 | 205.0972 | positive | C_11_H_12_N_2_O_2_ | 5.0 | 1.757 | 188.0710, 146.0603, 142.0653, 130.0651, 118.0654 | D-Tryptophan* |
| 70 | 3.40 | 151.0392 | positive | C_8_H_6_O_3_ | 5.5 | 1.519 | 123.0443, 107.0860, 105.0451 | Phenylglyoxylic acid |
| 71 | 3.46 | 159.0917 | positive | C_10_H_10_N_2_ | 3.0 | 1.643 | 142.0655, 132.0810, 130.0653, 118.0653, 115.0543, 105.0701 | 1,5-naphthalenediamine |
| 72 | 3.54 | 317.1231 | positive | C_14_H_20_O_8_ | 5.0 | -1.800 | 137.0603, 135.0445, 123.0442, 107.0499 | Vanilloloside |
| 73 | 3.56 | 188.0917 | positive | C_8_H_13_NO_4_ | 3.0 | 2.760 | 83.0492, 55.0542 | 2-keto-6-acetamidocaproate |
| 74 | 3.57 | 118.0863 | positive | C_5_H_11_NO_2_ | 1.0 | 1.722 | 72.0813, 56.0500 | *L*-Valine* |
| 75 | 3.58 | 579.1505 | positive | C_30_H_26_O_12_ | 4.5 | 1.377 | 561.1343, 289.0712, 287.0558, 271.0605, 269.0454, 257.0448, 245.0446, 231.0665, 203.0340, 147.0443, 137.0231, 127.0392 | Kaempferol 3-*O*-*α*-*L*-(3-*trans*-*p*-coumaroyl-rhamnopyranoside) |
| 76 | 3.62 | 180.0655 | positive | C_9_H_9_NO_3_ | 6.0 | 2.731 | 162.0924, 152.0712, 138.0543, 108.0448 | 4-acetamidobenzoic acid |
| 77 | 3.76 | 287.0557 | positive | C_15_H_10_O_6_ | 4.5 | 2.388 | 231.0657, 213.0550, 137.0233 | Kaempferol* |
| 78 | 3.76 | 449.1086 | positive | C_21_H_20_O_11_ | 2.5 | 1.697 | 287.0555, 91.0390 | Kaempferol 7-*O*-glucoside |
| 79 | 3.84 | 175.0612 | negative | C_7_H_12_O_5_ | 2.0 | 0.538 | 157.0508, 131.0715, 129.0558, 115.0402, 113.0609 | 2-isopropylmalic acid |
| 80 | 3.84 | 191.0561 | negative | C_7_H_12_O_6_ | 2.0 | 2.959 | 173.0094, 147.0298, 146.9386, 129.0194, 101.0247 | Quinic acid |
| 81 | 3.91 | 353.0878 | negative | C_16_H_18_O_9_ | 3.0 | 2.729 | 179.0344, 161.0239, 145.0501 | Chlorogenic acid* |
| 82 | 3.92 | 137.0244 | negative | C_7_H_6_O_3_ | 5.0 | 2.190 | 93.0331, 65.0384 | Salicylic acid* |
| 83 | 3.93 | 145.0506 | negative | C_6_H_10_O_4_ | 2.0 | 0.064 | 101.0608, 59.0139 | Adipic acid* |
| 84 | 3.96 | 163.0401 | negative | C_9_H_8_O_3_ | 1.0 | 1.122 | 93.0347, 65.0396 | *p*-Coumaric acid* |
| 85 | 4.10 | 303.0510 | negative | C_15_H_12_O_7_ | 3.0 | 3.111 | 151.0031, 125.0239, 83.0133 | Taxifolin* |
| 86 | 4.11 | 217.0974 | positive | C_12_H_12_N_2_O_2_ | 2.5 | 1.132 | 200.0703, 173.1080, 171.0923, 156.0809, 155.0716, 154.0656, 146.0970, 145.0762, 144.0811, 143.0728, 130.0656, 129.0702, 118.0655 | *L*-1,2,3,4-tetrahydro-*β*-carboline-3-carboxylic acid |
| 87 | 4.30 | 231.1131 | positive | C_13_H_14_N_2_O_2_ | 3.5 | 1.280 | 185.1080, 170.0969, 168.0811, 158.0967, 156.0811, 154.0654, 144.0811, 142.0655, 132.0811, 130.0654, 118.0653 | (1xi,3xi)-1,2,3,4-tetrahydro-1-methyl-*β*-carboline-3-carboxylic acid |
| 88 | 4.35 | 155.0703 | positive | C_8_H_10_O_3_ | 4.0 | 2.545 | 155.0800, 125.0604, 109.0285 | 2,6-dimethoxyphenol |
| 89 | 4.38 | 210.0774 | negative | C_10_H_13_NO_4_ | 5.5 | 1.263 | 166.0885, 136.0764 | 3-*O*-methyl-*α*-methyldopa |
| 90 | 4.39 | 291.0863 | positive | C_15_H_14_O_6_ | 5.0 | 3.483 | 273.0765, 249.0788, 151.0393, 147.0444, 139.0393, 123.0443 | Catechin* |
| 91 | 4.39 | 181.0495 | positive | C_9_H_8_O_4_ | 1.0 | 2.497 | 89.0427, 79.0536 | Caffeic acid* |
| 92 | 4.39 | 867.2131 | positive | C_45_H_38_O_18_ | 3.5 | 1.352 | 715.1628, 579.1510, 301.0726 | Procyanidin C2 |
| 93 | 4.41 | 123.0442 | positive | C_7_H_6_O_2_ | 4.5 | 1.170 | 91.0195, 69.0340 | 4-hydroxybenzaldehyde* |
| 94 | 4.41 | 139.0391 | positive | C_7_H_6_O_3_ | 4.5 | 0.931 | 121.0283, 91.0184, 73.0290 | 3,4-dihydroxybenzaldehyde* |
| 95 | 4.49 | 132.1019 | positive | C_6_H_13_NO_2_ | 1.0 | 1.728 | 69.0701, 59.0128,55.0542 | *L*-Leucine* |
| 96 | 4.53 | 191.0350 | negative | C_10_H_8_O_4_ | 3.0 | 2.675 | 149.0237, 147.0453 | Scopoletin* |
| 97 | 4.53 | 169.1223 | positive | C_10_H_16_O_2_ | 3.0 | 1.275 | 151.1121, 133.1016, 123.1172, 121.1012, 111.0805, 109.1015, 107.0857 | xi-1,8,8-trimethyl-2-oxabicyclo[3.2.1]octan-3-one |
| 98 | 4.72 | 131.0714 | negative | C_6_H_12_O_3_ | 1.0 | 2.089 | 113.0608, 89.0239, 71.0125 | 2-butoxyacetic acid |
| 99 | 4.76 | 179.0339 | positive | C_9_H_6_O_4_ | 2.0 | 3.279 | 123.0446, 105.0336 | Aesculetin |
| 100 | 4.82 | 169.0498 | positive | C_8_H_8_O_4_ | 4.5 | 1.566 | 151.0391, 125.0599, 109.0286 | Vanillic acid* |
| 101 | 4.90 | 153.0195 | negative | C_7_H_6_O_4_ | 5.5 | 2.266 | 135.0085, 109.0296 | 3,5-dihydroxybenzoic acid |
| 102 | 4.90 | 183.0663 | negative | C_9_H_12_O_4_ | 4.0 | 1.833 | 137.0250, 121.0298 | 1-(3-hydroxy-4-methoxyphenyl)-1,2-ethanediol |
| 103 | 4.90 | 319.0459 | negative | C_15_H_12_O_8_ | 5.0 | 3.847 | 165.0196, 153.0195, 137.0245, 109.0296 | *trans*-3,3',4',5,5',7-hexahydroxyflavanone |
| 104 | 4.92 | 165.0193 | negative | C_8_H_6_O_4_ | 1.5 | 3.453 | 137.0246, 121.0297, 108.9376 | Benzoquinoneacetic acid |
| 105 | 5.00 | 315.1958 | positive | C_20_H_26_O_3_ | 4.5 | 1.043 | 205.1581, 159.1170, 147.1169, 145.1013, 135.0807, 133.0646, 107.0857, 105.0700 | Kahweol |
| 106 | 5.05 | 609.1461 | negative | C_27_H_30_O_16_ | 1.5 | 2.080 | 301.0348, 163.0606, 109.0290 | Rutin* |
| 107 | 5.24 | 163.0403 | negative | C_9_H_8_O_3_ | 4.5 | 2.154 | 119.0503, 117.0355 | 2,3-dihydrobenzofuran-2-carboxylic acid |
| 108 | 5.28 | 153.0546 | positive | C_8_H_8_O_3_ | 5.0 | 1.606 | 111.0443, 97.0290, 69.0340 | Vanillin* |
| 109 | 5.32 | 465.1028 | positive | C_21_H_20_O_12_ | 2.5 | 3.057 | 303.0505, 285.0394 | Quercetin-7-*O*-*β*-D-glucoside* |
| 110 | 5.35 | 463.0887 | negative | C_21_H_20_O_12_ | 5.5 | 3.450 | 301.0357, 299.0185, 283.0249, 271.0251, 245.0450, 151.0039, 107.0142 | Isoquercitrin* |
| 111 | 5.55 | 109.0659 | negative | C_7_H_10_O | 3.0 | 2.026 | 93.0340, 69.0340 | (E,E)-2,4-heptadienal |
| 112 | 5.60 | 195.0652 | positive | C_10_H_10_O_4_ | 4.0 | 3.358 | 149.0603, 119.0497, 77.0391 | Ferulic acid* |
| 113 | 5.63 | 177.0549 | positive | C_10_H_8_O_3_ | 1.5 | 1.578 | 149.0238, 135.0443, 117.0337, 103.0546 | 7-hydroxy-6-methyl-2H-1-benzopyran-2-one |
| 114 | 5.92 | 183.1018 | positive | C_10_H_14_O_3_ | 3.5 | 1.251 | 165.0913, 105.0339 | Dihydroconiferyl alcohol |
| 115 | 6.07 | 197.0808 | positive | C_10_H_12_O_4_ | 5.0 | 2.067 | 155.0692 | Acetosyringone |
| 116 | 6.11 | 147.0441 | positive | C_9_H_6_O_2_ | 1.0 | 3.320 | 103.0548, 75.0235 | Coumarin* |
| 117 | 6.28 | 153.1274 | positive | C_10_H_16_O | 3.0 | 2.793 | 135.1172, 125.0967, 109.1014, 107.0858 | Camphor* |
| 118 | 6.35 | 167.1067 | positive | C_10_H_14_O_2_ | 4.0 | 2.435 | 149.0964, 107.0494 | 6-pentyl-2H-pyran-2-one |
| 119 | 7.01 | 195.1016 | positive | C_11_H_14_O_3_ | 5.0 | 1.946 | 177.0978, 163.0394, 121.0650, 107.0493 | Methoxyeugenol |
| 120 | 7.07 | 195.1381 | positive | C_12_H_18_O_2_ | 3.5 | 0.736 | 177.1276, 79.0548 | Sedanolide |
| 121 | 7.46 | 263.1289 | negative | C_15_H_20_O_4_ | 6.0 | 3.746 | 201.0916, 139.0395, 81.0340 | Abscisic acid* |
| 122 | 7.55 | 201.1135 | negative | C_10_H_18_O_4_ | 2.5 | 2.785 | 183.1029, 157.1235, 139.1130, 137.0971 | 2-ethylsuberic acid |
| 123 | 7.65 | 336.1236 | positive | C_20_H_17_NO_4_ | 1.5 | 1.682 | 321.1002, 320.0924 | Berberine* |
| 124 | 7.71 | 287.0550 | positive | C_15_H_10_O_6_ | 1.0 | 2.232 | 213.0552, 153.0188, 109.0290 | Luteolin* |
| 125 | 7.79 | 301.0354 | negative | C_15_H_10_O_7_ | 1.5 | 3.391 | 119.0501, 107.0136 | Quercetin* |
| 126 | 7.85 | 308.2224 | positive | C_18_H_29_NO_3_ | 4.5 | 1.232 | 122.0600, 79.0184 | Dihydrocapsaicin |
| 127 | 8.66 | 327.2180 | negative | C_18_H_32_O_5_ | 3.5 | 2.277 | 239.1295, 211.1342, 197.1177, 183.1390, 181.1239, 171.1028, 155.1082 | Corchorifatty acid F |
| 128 | 8.80 | 331.1540 | positive | C_19_H_22_O_5_ | 3.0 | 3.710 | 133.1014, 121.1015, 119.0857 | Gibberellin A7 |
| 129 | 8.92 | 293.2111 | positive | C_18_H_28_O_3_ | 5.0 | 3.777 | 205.1603, 133.1012, 131.0866, 119.0857, 117.0708, 105.0704 | 12-oxo phytodienoic acid |
| 130 | 9.08 | 109.0650 | positive | C_7_H_8_O | 3.5 | 1.912 | 93.0340, 79.0540, 57.0340 | Benzyl alcohol |
| 131 | 9.10 | 151.1117 | positive | C_10_H_14_O | 4.0 | 3.217 | 133.1013, 123.0807, 121.1016, 107.0856, 105.0702 | Carvone |
| 132 | 9.90 | 241.1448 | negative | C_13_H_22_O_4_ | 3.5 | 2.658 | 179.1443, 197.1550, 223.1336 | 2-carboxy-4-dodecanolide |
| 133 | 9.96 | 221.1902 | positive | C_15_H_24_O | 3.5 | 0.941 | 163.1485, 151.1485, 149.1339, 137.1328, 135.1172, 123.1174, 121.1012, 109.1014, 107.0858 | Caryophyllene oxide* |
| 134 | 10.09 | 193.0859 | positive | C_11_H_12_O_3_ | 6.0 | 2.749 | 161.0600, 137.0604, 133.0650, 109.0648 | Myristicin |
| 135 | 10.16 | 158.1539 | positive | C_9_H_19_NO | 1.0 | 3.899 | 116.1071, 102.0915 | 2,2,6,6-tetramethyl-4-piperidinol |
| 136 | 10.21 | 318.3003 | positive | C_18_H_39_NO_3_ | 0.0 | 1.584 | 300.2916 | 4-hydroxysphinganine |
| 137 | 10.22 | 135.1171 | positive | C_10_H_14_ | 3.5 | 2.020 | 119.0856, 107.0857, 105.0700 | *p*-Cymene |
| 138 | 10.24 | 329.1758 | negative | C_20_H_26_O_4_ | 1.0 | -1.698 | 271.0256, 127.1128 | Dicyclohexyl phthalate |
| 139 | 10.33 | 267.1605 | negative | C_15_H_24_O_4_ | 4.5 | 2.294 | 249.1495, 223.1706, 221.1552, 179.1440 | Prostaglandin lactone-diol |
| 140 | 10.39 | 194.1542 | positive | C_12_H_19_NO | 3.5 | 1.335 | 109.1013, 107.0855 | *N*-Cyclopropyl-trans-2-cis-6-nonadienamide |
| 141 | 10.56 | 267.1591 | positive | C_15_H_22_O_4_ | 4.5 | 0.054 | 249.1513, 203.1447, 139.0754, 135.1170, 125.0960, 125.0601 | Blennin D |
| 142 | 10.60 | 293.2111 | positive | C_18_H_28_O_3_ | 5.0 | 2.804 | 233.1545, 191.1431, 177.1276, 161.0963, 135.0812, 123.1173 | cis-12-oxophytodienoic acid |
| 143 | 10.67 | 331.2490 | negative | C_18_H_36_O_5_ | 1.0 | 1.738 | 269.1912, 189.1290, 187.0970, 157.0875 | Phloionolic acid |
| 144 | 10.78 | 365.1973 | negative | C_20_H_30_O_6_ | 2.5 | 2.929 | 347.1857, 321.2071, 303.1971, 277.2161, 275.2025, 233.1575 | 20-carboxy-leukotriene B4 |
| 145 | 10.83 | 135.0804 | positive | C_9_H_10_O | 5.0 | 2.336 | 119.0602, 109.0651, 107.0858 | 2,4-dimethylbenzaldehyde |
| 146 | 10.91 | 415.2122 | positive | C_24_H_30_O_6_ | 3.5 | 1.649 | 295.1189, 133.0649, 103.0544 | Clausarinol |
| 147 | 11.02 | 313.2387 | negative | C_18_H_34_O_4_ | 2.5 | 1.355 | 295.2279, 201.1134, 171.1028, 155.1078, 129.0923, 127.1130, 125.0972 | 9,10-dihydroxy-12-octadecenoic acid |
| 148 | 11.08 | 311.2228 | negative | C_18_H_32_O_4_ | 3.0 | 2.847 | 293.2124, 275.2022, 265.2170, 249.2208, 185.1186, 183.1031, 181.1237, 171.1028, 169.1236, 167.1071, 141.0934, 125.0974, 121.1022 | 9-hpode |
| 149 | 11.13 | 333.2075 | negative | C_20_H_30_O_4_ | 1.5 | 2.394 | 315.1959 | 15-keto-13,14-dihydroprostaglandin A2 |
| 150 | 11.16 | 303.2322 | positive | C_20_H_30_O_2_ | 5.5 | 1.132 | 243.2110, 241.1965, 215.1802, 201.1650, 189.1641, 187.1492, 175.1487, 161.1325, 149.1329, 147.1172, 135.1170, 133.1015, 121.1011, 109.1015, 107.0856 | Eicosapentaenoic acid |
| 151 | 11.17 | 269.0455 | negative | C_15_H_10_O_5_ | 4.0 | 3.426 | 181.0659, 169.0659, 133.0295 | Genistein* |
| 152 | 11.19 | 319.2282 | negative | C_20_H_32_O_3_ | 5.5 | 2.475 | 275.2387, 273.2217, 207.1756, 193.1598, 123.0809, | 8,9-epoxyeicosatrienoic acid |
| 153 | 11.24 | 319.2282 | negative | C_20_H_32_O_3_ | 5.5 | 1.475 | 275.2390, 273.2212, 219.1764 | 20-hydroxyeicosatetraenoic acid |
| 154 | 11.41 | 520.3406 | positive | C_26_H_50_NO_7_P | 2.5 | 1.603 | 184.0737, 125.0001, 104.1072 | LysoPC(0:0/18:2(9Z,12Z)) |
| 155 | 11.43 | 315.2541 | negative | C_18_H_36_O_4_ | 1.5 | 3.533 | 201.1133, 171.1027, 157.1236, 155.1078, 143.1078, 141.1290, 127.1130, 125.0970 | 9,10-Dihydroxystearic acid |
| 156 | 11.66 | 317.2111 | positive | C_20_H_28_O_3_ | 3.0 | 1.756 | 281.1904, 165.1277, 147.1167, 145.1017, 133.0650, 107.0857, 105.0703 | Cafestol |
| 157 | 11.95 | 279.1591 | positive | C_16_H_22_O_4_ | 4.0 | 3.377 | 195.1381, 177.1279, 163.1120, 153.0902, 119.0856, 117.0697 | 2,5,7,8-tetramethyl-2-(2'-carboxyethyl)-6-hydroxychroman |
| 158 | 11.95 | 357.2999 | positive | C_21_H_40_O_4_ | 2.0 | 3.740 | 339.2903, 283.2640, 265.2528 | Monoolein |
| 159 | 12.01 | 277.2173 | negative | C_18_H_30_O_2_ | 4.0 | 2.918 | 133.1014, 131.0855, 119.0860, 117.0703, 107.0857, 105.0701 | *α*-Eleostearic acid |
| 160 | 12.04 | 478.2939 | negative | C_23_H_46_NO_7_P | 2.5 | 3.312 | 281.2490, 140.0119, 122.0016 | LysoPE(0:0/18:1(11Z)) |
| 161 | 12.15 | 135.0804 | positive | C_9_H_10_O | 5.0 | 1.336 | 117.0701, 107.0857, 105.0701 | 4-ethylbenzaldehyde |
| 162 | 12.58 | 219.1746 | positive | C_15_H_22_O | 4.5 | 1.178 | 177.1639, 161.1327, 159.1172, 147.1171, 145.1019, 135.1168, 133.1018, 109.1012 | Nootkatone |
| 163 | 12.72 | 203.1797 | positive | C_15_H_22_ | 4.5 | 1.342 | 147.1171, 135.1178, 133.1015, 121.1012, 119.0858 | *α*-Curcumene |
| 164 | 13.05 | 273.2228 | negative | C_16_H_31_FO_2_ | 1.5 | 1.336 | 149.0978 | 2-fluoropalmitic acid |
| 165 | 13.40 | 205.1954 | positive | C_15_H_24_ | 3.5 | 1.572 | 149.1328, 123.1172, 121.1014, 109.1015, 107.0857, 105.0704 | *α*-Farnesene |
| 166 | 13.51 | 317.2125 | negative | C_20_H_30_O_3_ | 1.5 | 2.346 | 273.2227, 271.2075 | 5-KETE |
| 167 | 13.72 | 315.1970 | negative | C_20_H_28_O_3_ | 3.5 | 2.850 | 271.2070, 269.1914, 255.1754 | 15-deoxy-d-12,14-PGJ2 |
| 168 | 14.55 | 279.2324 | positive | C_18_H_30_O_2_ | 3.5 | 1.946 | 163.1484, 161.1330, 149.1326, 147.1173, 137.1327, 135.1166, 123.1171, 121.1013, 109.1014 | Calendic acid |
| 169 | 14.68 | 326.3061 | positive | C_20_H_39_NO_2_ | 1.5 | 2.280 | 265.2548, 69.0703 | Oleoylethanolamide |
| 170 | 15.57 | 282.2791 | positive | C_18_H_35_NO | 2.0 | 1.920 | 165.1637, 151.1480, 137.1329, 125.1330, 123.1170, 111.1171, 109.1012 | Oleamide* |
| 171 | 15.70 | 281.2475 | positive | C_18_H_32_O_2_ | 3.0 | 3.990 | 95.0855, 69.0701 | Linoleic acid* |

*: means the compounds were compared with standards

The other compounds were identified by searching the HMDB and PubChem database

**Table 7S.2** Tentative characterization of chemical constituents of SR by HS-GC-MS/MS

| No. | *t_R_* (min) | Molecular Weight (MW) | Formula | Identification | Matching score |
| --- | --- | --- | --- | --- | --- |
| 1 | 7.579 | 136.12 | C_10_H_16_ | Tricyclene | 95.01 |
| 2 | 7.716 | 136.12 | C_10_H_16_ | *α*-Phellandrene | 95.51 |
| 3 | 7.897 | 136.12 | C_10_H_16_ | *α*-Pinene | 96.75 |
| 4 | 8.307 | 136.12 | C_10_H_16_ | Camphene | 96.04 |
| 5 | 8.604 | 106.04 | C_7_H_6_O | Benzaldehyde | 89.87 |
| 6 | 8.974 | 136.12 | C_10_H_16_ | *β*-Phellandrene | 94.12 |
| 7 | 9.053 | 136.12 | C_10_H_16_ | *β*-Pinene | 94.90 |
| 8 | 9.439 | 136.12 | C_10_H_16_ | *β*-Myrcene | 94.19 |
| 9 | 9.799 | 136.12 | C_10_H_16_ | 3-thujene | 90.65 |
| 10 | 10.133 | 136.12 | C_10_H_16_ | 4-carene | 94.85 |
| 11 | 10.350 | 134.11 | C_10_H_14_ | *p*-Cymene | 94.33 |
| 12 | 10.480 | 136.12 | C_10_H_16_ | D-Limonene | 95.15 |
| 13 | 10.533 | 154.13 | C_10_H_18_O | Eucalyptol | 92.69 |
| 14 | 10.857 | 120.06 | C_8_H_8_O | Benzeneacetaldehyde | 89.68 |
| 15 | 11.264 | 136.12 | C_10_H_16_ | *γ*-Terpinene | 90.90 |
| 16 | 11.500 | 154.13 | C_10_H_18_O | *trans*-Sabinene hydrate | 95.67 |
| 17 | 11.663 | 170.13 | C_10_H_18_O_2_ | *trans*-Linalyl oxide | 96.17 |
| 18 | 12.106 | 154.13 | C_10_H_18_O | *β*-Terpineol | 81.93 |
| 19 | 12.407 | 136.12 | C_10_H_16_ | 3-carene | 95.36 |
| 20 | 12.594 | 154.13 | C_10_H_18_O | Linalool | 92.64 |
| 21 | 13.383 | 152.12 | C_10_H_16_O | *α*-Campholenal | 86.70 |
| 22 | 14.280 | 152.12 | C_10_H_16_O | Camphor | 94.32 |
| 23 | 14.446 | 154.13 | C_10_H_18_O | Isoborneol | 97.17 |
| 24 | 14.620 | 150.10 | C_10_H_14_O | Pinocarvone | 93.76 |
| 25 | 14.794 | 154.13 | C_10_H_18_O | Borneol | 95.51 |
| 26 | 15.072 | 154.13 | C_10_H_18_O | Terpinen-4-ol | 93.04 |
| 27 | 15.260 | 132.09 | C_10_H_12_ | Benzene,1-methyl-4-(1-methylethenyl) | 85.33 |
| 28 | 15.548 | 154.13 | C_10_H_18_O | *α*-Terpineol | 91.95 |
| 29 | 15.671 | 150.10 | C_10_H_14_O | Myrtenal | 93.92 |
| 30 | 16.077 | 150.10 | C_10_H_14_O | 4,6,6-trimethylbicyclo [3.1.1]Hept-3-en-2-one | 91.47 |
| 31 | 16.367 | 154.13 | C_10_H_18_O | *γ*-Terpineol | 90.76 |
| 32 | 16.487 | 152.12 | C_10_H_16_O | *trans*-Carveol | 92.26 |
| 33 | 16.622 | 182.13 | C_11_H_18_O_2_ | Bornyl formate | 94.88 |
| 34 | 16.958 | 148.09 | C_10_H_12_O | 3-isopropylbenzaldehyde | 85.87 |
| 35 | 17.098 | 150.10 | C_10_H_14_O | D-Carvone | 92.74 |
| 36 | 17.364 | 190.17 | C_14_H_22_ | 1,3-bis-(1,1-dimethylethyl)-benzene | 88.19 |
| 37 | 18.586 | 196.14 | C_12_H_20_O_2_ | Bornyl acetate | 97.13 |
| 38 | 19.645 | 204.19 | C_15_H_24_ | *γ*-Elemene | 92.07 |
| 39 | 19.951 | 204.19 | C_15_H_24_ | *α*-Copaene | 95.87 |
| 40 | 20.252 | 196.14 | C_12_H_20_O_2_ | Linalyl acetate | 80.63 |
| 41 | 20.421 | 204.19 | C_15_H_24_ | Ylangene | 89.56 |
| 42 | 20.534 | 204.19 | C_15_H_24_ | *β*-Copaene | 94.95 |
| 43 | 20.670 | 204.19 | C_15_H_24_ | *α*-Cubebene | 94.42 |
| 44 | 20.886 | 204.19 | C_15_H_24_ | *β*-Elemene | 92.34 |
| 45 | 21.091 | 204.19 | C_15_H_24_ | *β*-Cedrene | 96.11 |
| 46 | 21.636 | 204.19 | C_15_H_24_ | Aristolene | 93.90 |
| 47 | 21.748 | 204.19 | C_15_H_24_ | *α*-Cedrene | 90.79 |
| 48 | 21.922 | 204.19 | C_15_H_24_ | Longifolene | 95.33 |
| 49 | 22.046 | 204.19 | C_15_H_24_ | *β*-Gurjunene | 89.00 |
| 50 | 22.227 | 204.19 | C_15_H_24_ | 1-epi-bicyclosesquiphellandrene | 90.64 |
| 51 | 22.386 | 204.19 | C_15_H_24_ | *α*-Bergamotene | 96.93 |
| 52 | 22.487 | 204.19 | C_15_H_24_ | *α*-Guaiene | 86.75 |
| 53 | 22.541 | 204.19 | C_15_H_24_ | *β*-Panasinsene | 83.16 |
| 54 | 22.773 | 204.19 | C_15_H_24_ | *β*-Santalene | 85.24 |
| 55 | 22.992 | 204.19 | C_15_H_24_ | *β*-Farnesene | 92.01 |
| 56 | 23.160 | 204.19 | C_15_H_24_ | Bicyclo[2.2.1]heptane, 2-methyl-3-methylene-2-(4-methyl-3-pentenyl)-, (1*S*-exo)- | 81.61 |
| 57 | 23.233 | 204.19 | C_15_H_24_ | Aromadendrene | 95.69 |
| 58 | 23.600 | 204.19 | C_15_H_24_ | Epizonarene | 86.10 |
| 59 | 23.685 | 204.19 | C_15_H_24_ | *γ*-Muurolene | 93.77 |
| 60 | 23.851 | 204.19 | C_15_H_24_ | Germacrene D | 94.15 |
| 61 | 23.925 | 204.19 | C_15_H_24_ | Caryophyllene | 88.58 |
| 62 | 24.017 | 204.19 | C_15_H_24_ | *β*-Humulene | 93.39 |
| 63 | 24.149 | 204.19 | C_15_H_24_ | *ω*-Cadinene | 91.12 |
| 64 | 24.330 | 204.19 | C_15_H_24_ | *α*-Gurjunene | 96.30 |
| 65 | 24.408 | 204.19 | C_15_H_24_ | *α*-Muurolene | 94.53 |
| 66 | 24.632 | 204.19 | C_15_H_24_ | *α*-Himachalene | 94.61 |
| 67 | 24.722 | 204.19 | C_15_H_24_ | *γ*-Curcumene | 88.03 |
| 68 | 24.864 | 204.19 | C_15_H_24_ | *γ*-Cadinene | 92.04 |
| 69 | 25.107 | 204.19 | C_15_H_24_ | *Δ*-Amorphene | 96.20 |
| 70 | 25.357 | 204.19 | C_15_H_24_ | *β*-Bisabolene | 92.16 |
| 71 | 25.691 | 204.19 | C_15_H_24_ | 2-epi-*α*-cedrene | 84.82 |
| 72 | 26.163 | 204.19 | C_15_H_24_ | Germacrene B | 84.79 |
| 73 | 26.770 | 220.18 | C_15_H_24_O | Spathulenol | 96.37 |
| 74 | 26.948 | 220.18 | C_15_H_24_O | Caryophyllene oxide | 89.71 |
| 75 | 27.060 | 220.18 | C_15_H_24_O | *α*-Cyperol | 83.92 |
| 76 | 29.005 | 204.19 | C_15_H_24_ | 1,4-dimethyl-7-(1-methylethenyl)-octahydroazulene | 87.18 |
| 77 | 29.395 | 222.20 | C_15_H_26_O | Sesquisabinene hydrate | 82.05 |
| 78 | 29.782 | 222.20 | C_15_H_26_O | *α*-Bisabolol | 84.27 |

**Table 7S.3** Tentative characterization of chemical constituents of SR by NMR

| No. | Identification | *δ*_H_ (ppm) | *δ*_C_ (ppm) |
| --- | --- | --- | --- |
| 1 | *L*-Histidine | 7.89 (s), 7.07 (s), 3.92 (s), 3.25 (m), 3.13 (m) | 57.23, 29.55 |
| 2 | Trehalose | 5.19 (d), 3.83 (m), 3.65 (d), 3.48 (t) | 95.81, 75.44, 73.89, 72.45, 63.45 |
| 3 | Xylitol | 3.79 (d), 3.73(s), 3.71 (s), 3.64 (d) | 73.89, 65.51 |
| 4 | Citric acid | 2.68 (d), 2.66 (d), 2.54 (d), 2.41 (s), 2.39 (s) | 74.10 |
| 5 | *L*-Threonine | 4.23 (d), 3.57 (dd), 1.33 (d) | 63.24 |
| 6 | Choline | 4.06 (t), 3.50 (t), 3.20 (s) | 70.29 |
| 7 | Mannose | 5.23 (d), 4.00 (d), 3.82 (m), 3.72 (d), 3.71 (d), 3.46 (t) | 72.76, 70.29, 61.29 |
| 8 | Maltol | 7.74 (d), 7.69 (d), 6.49 (m), 2.39 (s) | 14.43 |
| 9 | Glucogallin | 7.02 (s), 5.01 (s), 4.11 (d), 4.04 (t), 4.01(d), 3.91 (m), 3.73 (m), 3.61 (s) | 78.36, 72.66, 71.01, 62.52 |
| 10 | Theophylline | 8.46 (s), 8.00 (s), 3.51 (s), 3.36 (s) | 101.51 |
| 11 | Pantothenic acid | 4.00 (s), 3.43 (t), 2.41 (s) | 71.01, 23.12 |
| 12 | 2-isopropylmalic acid | 2.54 (d), 2.11 (s), 1.80 (t) | 43.18 |
| 13 | Vanillic acid | 7.54 (s), 7.47 (s), 6.94 (m), 3.90 (m) | 58.61 |
| 14 | Abscisic acid | 7.47 (s), 7.00 (s), 5.90 (m), 5.74 (m), 2.41 (s), 2.07 (s), 1.90 (s), 1.05 (s) | 23.12, 21.06 |
| 15 | Benzyl alcohol | 7.42 (s), 7.07 (s), 4.65 (d) | 66.44 |
| 16 | *p*-Cymene | 7.07 (s), 2.81 (s), 2.30 (s), 1.23 (d) | 33.77, 24.57, 21.06 |
| 17 | Isoquercitrin | 6.94 (s), 6.91 (s), 6.90 (s), 6.89 (s), 6.77 (s), 6.13 (d), 6.08 (d), 3.86 (m), 3.84 (s), 3.76 (s) | 103.63, 98.05, 75.18, 71.00, 62.32 |
| 18 | 9,10-dihydroxy-12-octadecenoic acid | 5.42 (d), 4.21 (d), 3.82 (m), 2.41 (s), 2.30 (s), 1.48 (d), 1.37 (s), 1.23 (d), 0.89 (s) | 73.89, 70.39, 34.18, 29.55 |
| 19 | Eicosapentaenoic acid | 5.43 (d), 2.41 (s), 2.35 (s), 1.95 (s), 0.97 (s) | 29.65, 26.11, 24.57 |
| 20 | Stearic acid | 2.30 (s), 1.33 (d), 1.29 (d), 0.89 (s) | 34.08, 29.65 |
| 21 | Arabinose | 5.23 (d), 4.12 (d), 4.00 (d), 3.87 (m), 3.82 (m), 3.69 (s) | 72.35, 70.39 |
| 22 | Glucose | 5.23 (d), 4.04 (d), 3.82 (m), 3.74 (t), 3.52 (s), 3.39 (s) | 72.76, 70.29, 61.39 |
| 23 | Hyperoside | 6.93 (m), 6.83 (m), 6.71 (m), 6.13 (d), 6.07 (d), 4.97 (d), 4.27 (m), 3.84 (m), 3.75 (s), 3.53 (d), 3.43 (s) | 103.66, 97.99, 78.27, 77.43, 75.16, 70.26, 62.34 |
| 24 | Galactose | 5.23 (d), 4.12 (d), 3.92 (m), 3.81 (m), 3.73 (t), 3.50 (t) | 72.76, 70.39, 61.39 |
| 25 | Malic acid | 4.29 (dd), 2.66 (dd), 2.36 (d) | 45.65 |
| 26 | *p*-Coumaric acid | 7.72 (dd), 7.39 (d), 6.77 (s), 6.53 (s) | 116.68 |
| 27 | Ferulic acid | 7.33 (s), 7.21(m), 7.16 (s), 6.90 (d), 6.38 (m), 3.89 (d) | 55.79 |
| 28 | Syringic acid | 7.07 (s), 6.77 (s), 3.83 (m) | 60.72 |
| 29 | Caffeic acid | 7.54 (d), 7.02 (s), 6.97 (m), 6.82 (m), 6.29 (d) | 127.28, 116.48 |
| 30 | 4-hydroxybenzoic acid | 7.81 (m), 6.91 (d) | 178.18 |
| 31 | Catechin | 6.83 (m), 6.76 (m), 6.71 (m), 5.92 (m), 5.80 (d), 4.51 (m), 3.93 (d), 2.81 (s), 2.53 (d) | 157.31 |
| 32 | Epicatechin | 7.03 (d), 6.95 (m), 6.13 (d), 4.97 (d), 4.32 (dd), 2.94 (m), 2.75 (m) | 98.05, 78.23, 28.53 |
| 33 | Quercetin | 7.69 (d), 7.54 (d), 6.90 (d), 6.43 (m), 6.21 (m) | 176.82 |
| 34 | Rutin | 7.02 (s), 6.91 (s), 6.71 (m), 6.08 (s), 5.19 (d), 4.97 (d), 4.35 (s), 4.12 (d), 4.04 (s), 3.83 (m), 3.71 (s), 3.50 (s), 1.37 (s) | 103.63, 101.44, 75.66, 74.40, 71.46, 69.53, 67.45 |
| 35 | Isorhamnetin | 8.26 (s), 8.24 (s), 6.97 (m), 6.47 (m), 6.21 (m), 3.83 (m) | 103.56, 98.17 |
| 36 | Pulegone | 2.33 (s), 2.11 (s), 1.88 (s), 1.75 (m), 1.48 (d), 1.37 (d), 0.90 (s) | 21.51 |
| 37 | Quercitrin | 12.69 (m), 10.90 (m), 9.76 (m), 9.37 (m), 7.33 (s), 7.28 (s), 6.90 (d), 6.42 (m), 6.29 (m), 5.24 (d), 4.98 (d), 4.81 (s), 4.64 (d), 4.00 (d), 3.51 (s), 3.20 (s), 0.82 (s) | 101.44, 98.07, 71.32 |


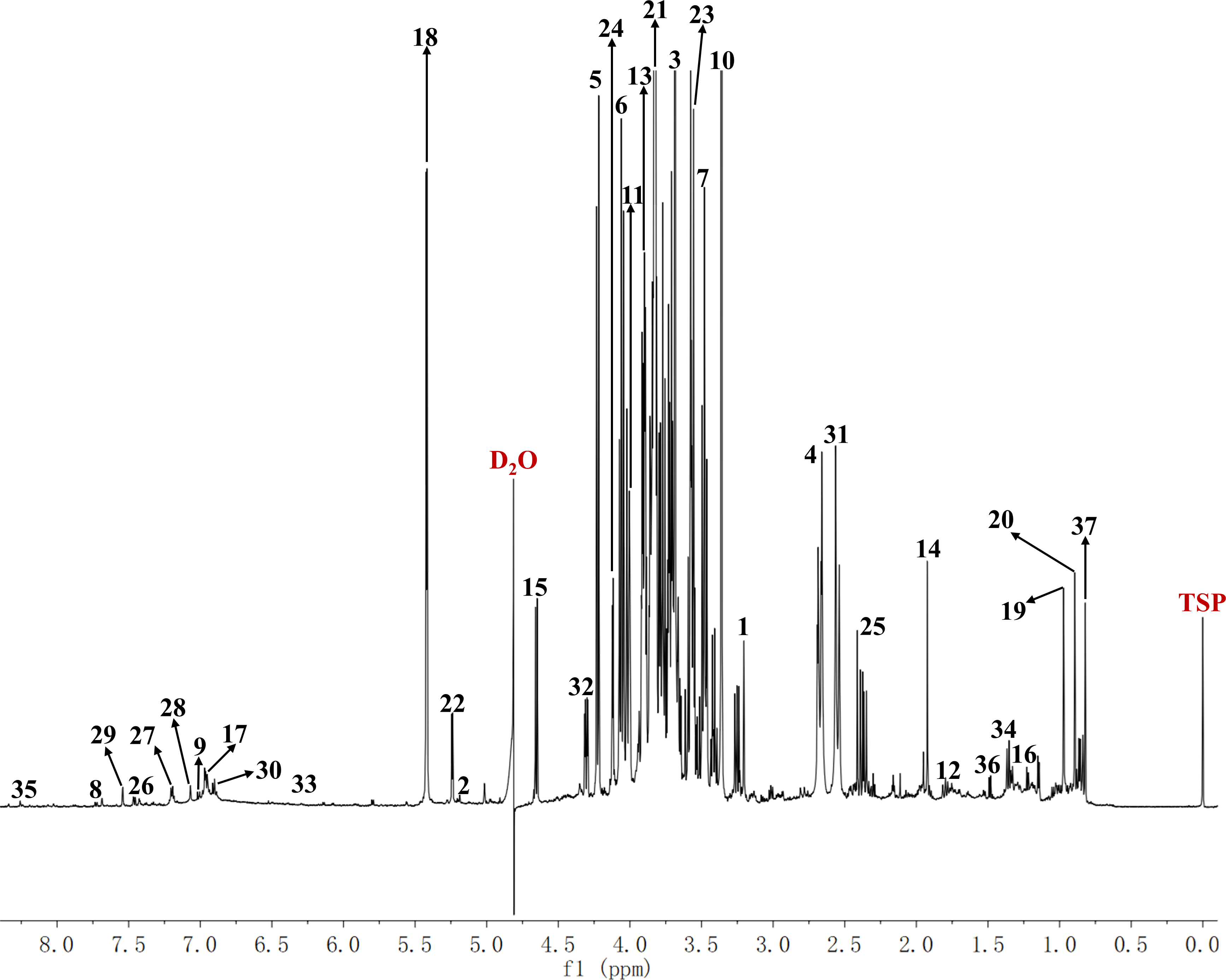


**Fig. 7S.1** Tentative characterization of chemical constituents of SR by NMR

8S. The results of data fusion models

The PLS-DA, SVM, *k*NN, NN, DT, and RF models based on individual model data set and the LLDF, MLDF models were established.


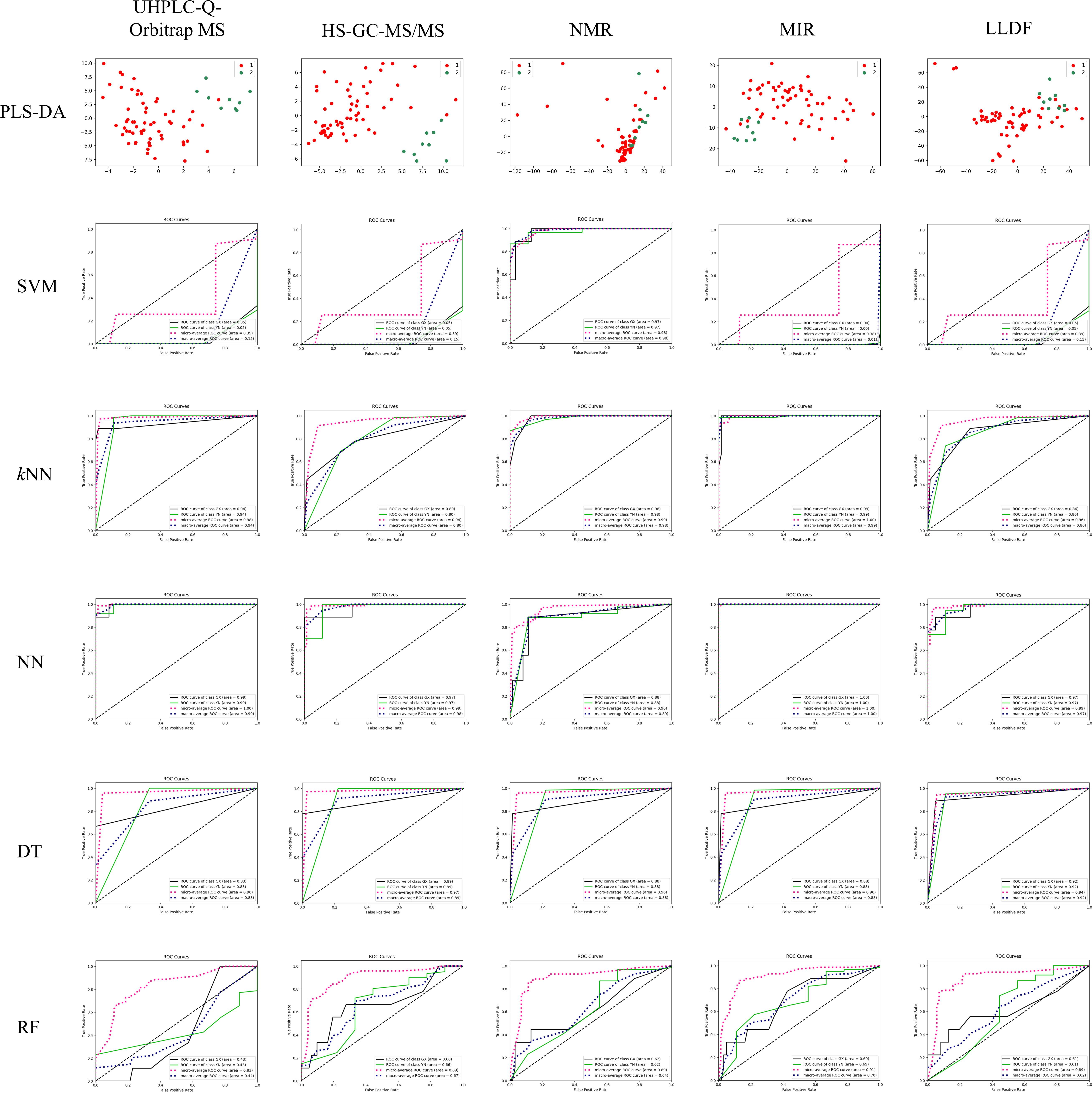


**Fig. 8S.1** The modeling effects of classification between YN (1) and GX (2) groups modeled by individual data matrices and LLDF


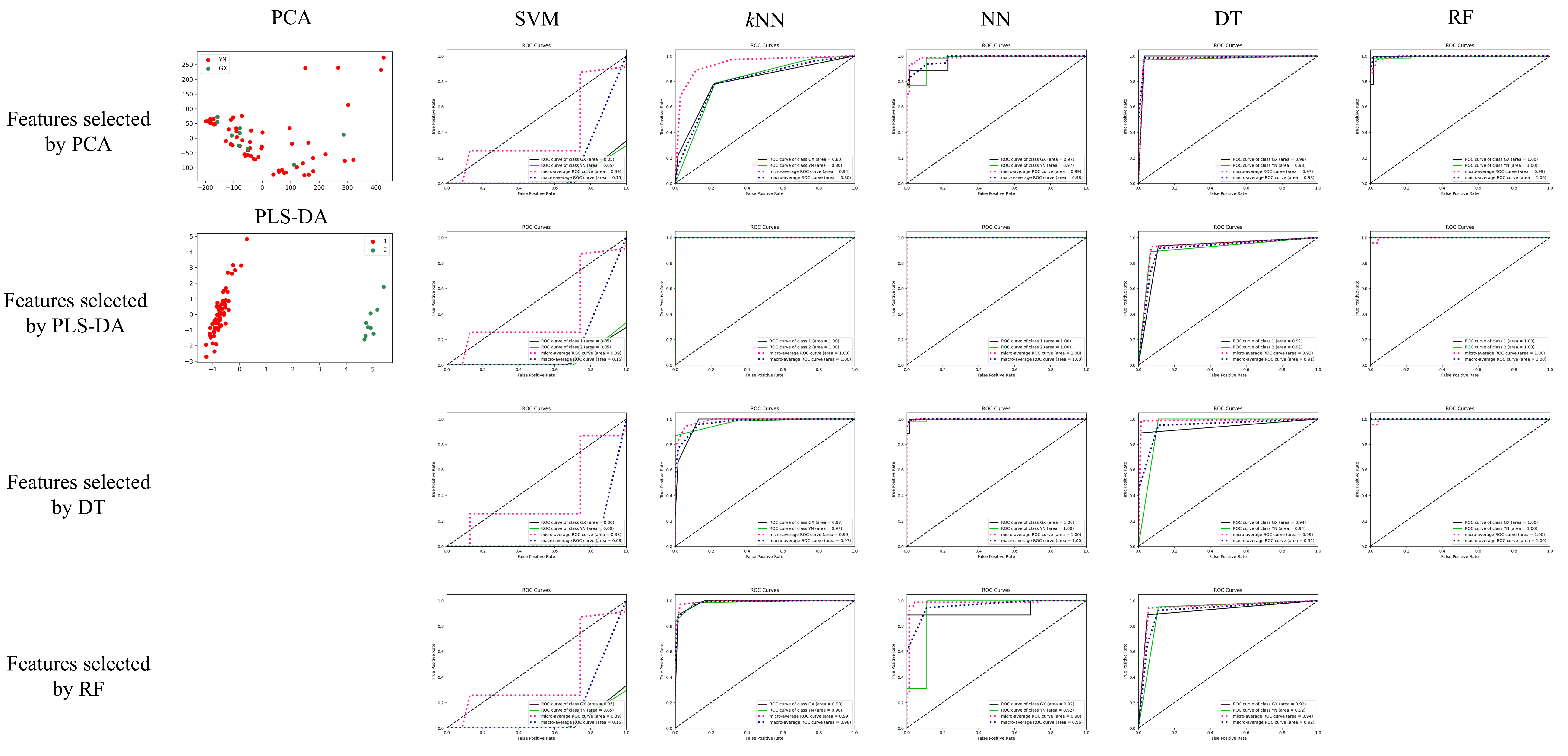


**Fig. 8S.2** The modeling effects of classification between YN (1) and GX (2) groups modeled by MLDF (ROC curves)

9S. The MS/MS spectra of the key differential metabolites screened from RF-RF model

The MS/MS spectra of the key differential metabolites (24 differential metabolites detected by UHPLC-Q-Orbitrap MS and HS-GC-MS/MS) were shown in Fig. 9S.1 for verification.





**Fig. 9S.1** The MS/MS spectra of the key differential metabolites (24 differential metabolites detected by UHPLC-Q-Orbitrap MS and HS-GC-MS/MS, the serial numbers corresponded to those in Table 2)
